# Co-targeting HSP90 alpha and CDK7 overcomes resistance against HSP90 inhibitors in BCR-ABL1+ leukemia cells

**DOI:** 10.1038/s41419-023-06337-3

**Published:** 2023-12-06

**Authors:** Melina Vogt, Niklas Dienstbier, Julian Schliehe-Diecks, Katerina Scharov, Jia-Wey Tu, Philip Gebing, Julian Hogenkamp, Berna-Selin Bilen, Silke Furlan, Daniel Picard, Marc Remke, Layal Yasin, David Bickel, Munishikha Kalia, Alfredo Iacoangeli, Thomas Lenz, Kai Stühler, Aleksandra A. Pandyra, Julia Hauer, Ute Fischer, Rabea Wagener, Arndt Borkhardt, Sanil Bhatia

**Affiliations:** 1 Department of Pediatric Oncology, Hematology and Clinical Immunology, Medical Faculty, Heinrich Heine University Düsseldorf, Düsseldorf, Germany; 2 German Cancer Consortium (DKTK), partner site Essen/Düsseldorf, Düsseldorf, Germany; 3 Interuniversity Institute of Bioinformatics in Brussels, ULB-VUB, Brussels, Belgium; 4 Structural Biology Brussels, Vrije Universiteit Brussel, Brussels, Belgium; 5 Department of Biostatistics and Health Informatics, King’s College London, London, UK; 6 Department of Basic and Clinical Neuroscience, King’s College London, Maurice Wohl Clinical Neuroscience Institute, London, UK; 7 National Institute for Health Research Biomedical Research Centre and Dementia Unit at South London and Maudsley NHS Foundation Trust and King’s College London, London, UK; 8 Molecular Proteomics Laboratory, Biological Medical Research Center, Heinrich-Heine-University Düsseldorf, Düsseldorf, Germany; 9 Institute for Molecular Medicine, Proteome Research, University Hospital and Medical Faculty, Heinrich-Heine-University Düsseldorf, Düsseldorf, Germany; 10 Institute of Clinical Chemistry and Clinical Pharmacology, University Hospital Bonn, Bonn, Germany; 11 German Center for Infection Research (DZIF), Partner Site Bonn-Cologne, Bonn, Germany; 12 Department of Pediatrics and Children’s Cancer Research Center, Children’s Hospital Munich Schwabing, Technical University of Munich, School of Medicine, Munich, Germany

**Keywords:** Acute lymphocytic leukaemia, High-throughput screening, Chaperones

## Abstract

HSP90 has emerged as an appealing anti-cancer target. However, HSP90 inhibitors (HSP90i) are characterized by limited clinical utility, primarily due to the resistance acquisition via heat shock response (HSR) induction. Understanding the roles of abundantly expressed cytosolic HSP90 isoforms (α and β) in sustaining malignant cells’ growth and the mechanisms of resistance to HSP90i is crucial for exploiting their clinical potential. Utilizing multi-omics approaches, we identified that ablation of the HSP90β isoform induces the overexpression of HSP90α and extracellular-secreted HSP90α (eHSP90α). Notably, we found that the absence of HSP90α causes downregulation of PTPRC (or CD45) expression and restricts in vivo growth of BCR-ABL1+ leukemia cells. Subsequently, chronic long-term exposure to the clinically advanced HSP90i PU-H71 (Zelavespib) led to copy number gain and mutation (p.S164F) of the *HSP90AA1* gene, and HSP90α overexpression. In contrast, acquired resistance toward other tested HSP90i (Tanespimycin and Coumermycin A1) was attained by MDR1 efflux pump overexpression. Remarkably, combined CDK7 and HSP90 inhibition display synergistic activity against therapy-resistant BCR-ABL1+ patient leukemia cells via blocking pro-survival HSR and HSP90α overexpression, providing a novel strategy to avoid the emergence of resistance against treatment with HSP90i alone.

## Introduction

Cancer cells are widely known to hijack normal cytoprotective processes mediated by chaperone proteins to promote their survival and growth [1]. Among the chaperone proteins, HSP90 has been extensively studied due to their critical ATP-dependent chaperone activity, required by various oncoproteins implicated in malignant transformation [2, 3]. HSP90 facilitates the correct folding of newly synthesized and denatured oncoproteins, such as BCR-ABL1 [4, 5]. Consistently, in a recent report, inhibition of HSP90 delays the progression of BCR-ABL1+ leukemia in combination with tyrosine kinase inhibitor (TKI) [6]. Of note, HSP90i are effective against TKI-resistant BCR-ABL1+ leukemia stem cells and BCR-ABL1^T315I^ mutant cells [4, 7–9]. Furthermore, HSP90 expression is found enriched in other therapy refractory leukemia subtypes, including acute or chronic myeloid leukemia (AML or CML) [10–13] and BCR-ABL1-like BCP-ALL [14, 15]. The critical involvement of HSP90 in numerous oncogenic pathways and its overexpression in poor prognostic leukemia subgroups positioned it as an important therapeutic target [2]. Numerous pan-HSP90i or isoform-specific HSP90i have been developed over the past few years, exhibiting different binding modes [16, 17]. However, despite the early clinical promise, adverse events, including resistance-acquisition and dose-limiting toxicity in patients, have mostly barred widespread use of HSP90i in the clinic [2]. Recently, the HSP90i PU-H71 (Zelavespib) has been granted orphan drug status by the US Food and Drug Association (FDA) to treat myelofibrosis and was administered for compassionate use to treat AML [18]. Nevertheless, the induction of HSR is acknowledged as one of the most prominent causes of acquired resistance toward using HSP90i [4, 19].

In mammalian cells, there are two cytosolic isoforms of HSP90, i.e., a stress-inducible HSP90α isoform (encoded by the *HSP90AA1* gene located on chromosome 14q32–33) and a constitutively expressed HSP90β isoform (*HSP90AB1* gene; located on chromosome 6p21) [20]. These isoforms share a high degree (86%) of amino acid sequence identity. Although HSP90α and HSP90β isoforms exhibit comparable affinities for their client proteins and can often compensate for each other effectively [21], exceptions have been reported that indicate distinctive binding tendencies [22–24]. This is emphasized by their different roles in development and cell survival [20]. For instance, HSP90α-KO mice develop normally or occasionally with few congenital disabilities [25], while the knockout of HSP90β causes embryonic lethality in mice, which cannot be compensated by HSP90α [26]. In previous ex vivo studies, cultured HSP90α-KO cells exhibited normal cell morphology and growth rates, while the generation of HSP90β-KO cells was not achieved [21].

In this study, we employed genetic KO and knockdown (KD) models of HSP90 isoforms (α and β) for extensive multi-omics-based in vitro and in vivo characterization and identified HSP90α as the primary driver of malignancy of the two isoforms in BCR-ABL1+ leukemia cells. Moreover, acquired resistance toward distinct HSP90i (exhibiting different binding modes) was studied in BCR-ABL1+ leukemia cells, highlighting the involvement of heightened HSP90α or MDR1 levels in mitigating the efficacy of HSP90 inhibition. Importantly, combinatorial ex vivo drug sensitivity screenings identified CDK7 inhibitors (CDK7i) as drugs synergizing with HSP90α inhibition. Thus our findings can augment HSP90i-based therapy and indicate a new therapeutic vulnerability, especially in cases of BCR-ABL1+ leukemia with reduced treatment response.

## Results

### HSP90β loss induces overexpression of the stress-inducible HSP90α isoform in BCR-ABL1+ cell lines (K562 and KCL22)

**Fig. 1:**
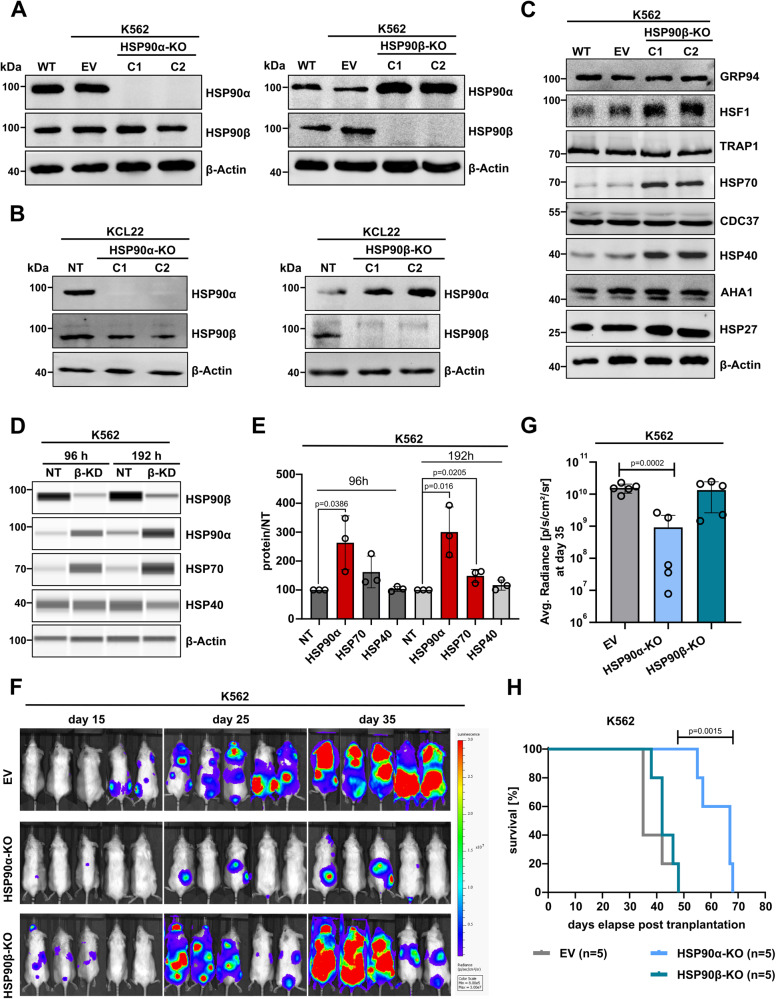
HSP90β ablation causes HSP90α and HSP70 upregulation, whereas HSP90α loss suppresses in vivo growth of BCR-ABL1+ leukemia cells and prolongs survival. Western Blot (WB) analysis of the stable CRISPR-Cas9 mediated knockout (KO) of HSP90α and HSP90β isoforms in K562 (**A**) and in KCL22 (**B**) cells. Clone (C), empty vector (EV), wild type (WT) and non-targeting (NT) control. Beta-actin (β-actin) served as a loading control. Representative immunoblots are shown from three independent repeats. **C** Expression of other non-cytosolic HSP90 paralogues (GRP94 and TRAP1), HSR-related proteins (HSP70, HSP40 and HSP27) and HSP90 co-chaperones (AHA1 and CDC37) in HSP90β-KO K562 cells. β-actin served as a loading control. **D** Expression of HSP70, HSP40 and HSP90α upon siRNA mediated HSP90β knockdown (KD) in K562 cells, analyzed by automated WB (JESS). β-actin served as a loading control. **E** Bars show average protein quantification measurements of HSP90α, HSP70 and HSP40 levels in HSP90β-KD cells compared to NT control cells. Error bars = SD of three independent replicates; *p*-values were calculated by unpaired two-tailed student’s t-test. **F** Images of NSG mice (*n* = 5 mice/group) transplanted with luciferase-GFP + HSP90α/β-KO or EV K562 cells on the days depicted outside image panel. **G** Graph show mean ± SD (*n* = 5 mice/group) of the bioluminescence measurements of region of interest (radiance; p/s/cm2/sr) at day 35. Significant reduction in the transplantation of HSP90α-KO as compared to EV control K562 cells, determined by unpaired two-tailed student’s t-test, *p* = 0.0002. **H** Kaplan–Meier survival curves showing significantly prolonged overall survival of NSG mice transplanted with HSP90α-KO compared to HSP90β-KO or EV (K562) control cells (*n* = 5 mice/group, *p* = 0.0015, Log-rank Mantel-Cox test).

To understand the precise role of HSP90 cytosolic isoforms (α and β), we generated CRISPR-Cas9 mediated knockout (KO), si- or inducible shRNA mediated knockdown (KD) models. Strikingly, the loss of HSP90β isoform in BCR-ABL1+ leukemia cell lines (K562 and KCL22) resulted in the upregulation of the HSP90α isoform both at protein and mRNA levels (Fig. 1A, B, Supplemental Fig. 1A–D). We next asked whether the observed high HSP90α expression upon loss of the HSP90β isoform was caused by genetic alterations of the *HSP90AA1* gene, as a long-term compensatory adaption carried out by HSP90β-KO cells. For that we performed SNP array analysis on the HSP90β-KO (K562) cells; however, no alterations were observed in the *HSP90AA1* locus (Supplemental Fig. 1E). Of note, HSP90α-KO did not induce the expression of HSR-related proteins (e.g., HSP70, HSP40, or HSP40), while HSP90β-KO resulted in the upregulation HSP70 and HSP40 (Fig. 1C, Supplemental Fig. 1F). Furthermore, analyzing changes in other non-cytosolic HSP90 paralogues, such as HSP75/TRAP1 (mitochondria), GRP94 (endoplasmic reticulum) and co-chaperones of HSP90 (AHA1 and CDC37) revealed no significant changes in their expression upon HSP90α/β loss (Fig. 1C, Supplemental Fig. 1F).

To corroborate the observed HSP90α overexpression in HSP90β-KO cells and to eliminate any potential off-target effects attributed to CRISPR-mediated targeting, we proceeded to employ a siRNA-mediated knockdown (KD) strategy using K562 cells. We observed that even a short-term KD of HSP90β, lasting either 96 or 192 h, induces a significant (*p* < 0.05) increase in HSP90α expression (Fig. 1D, E). However, a significant (*p* < 0.05) elevation in HSP70 expression following HSP90β-KD was only evident at a later time point (192 h). Conversely, HSP90β-KD did not affect the expression of HSP40 as seen in case of HSP90β-KO (Fig. 1D, E). In alternative models utilizing doxycycline-inducible shRNA to induce HSP90β-KD in K562 cells, we again validated increase in the HSP90α and HSP70 mRNA transcripts upon targeting of the HSP90β isoform (Supplemental Fig. 1G, H).

Following previous reports [22, 27–30], the binding preferences of specific client proteins (CDK4, CDK6, and SURVIVIN) toward distinct HSP90α/β isoforms were next analyzed. However, no changes were observed in the expression of these client proteins upon ablation of HSP90α/β isoforms (Supplemental Fig. 1I, J). On the other hand, the levels of other HSP90 client proteins, such as pan-AKT and FKBP5 [31, 32], were found enriched in both HSP90α- and HSP90β-KO cells (Supplemental Fig. 1I–K). Our subsequent focus was directed towards exploring the influence on the BCR-ABL1 oncoprotein following the depletion of HSP90α/β isoforms, considering the involvement of HSP90 in ensuring the accurate folding and subcellular positioning of the BCR-ABL1 protein [4, 5]. Relatively higher BCR-ABL1 activity (p-BCR-ABL^Y412^) along with heightened downstream pro-survival signaling (p-STAT5a^Y694^ and p-CRKL^Y207^) were noticed in HSP90α-KO cells compared to respective controls (Supplemental Fig. 1K, L). Moreover, immunofluorescence imaging of the HSP90α- and HSP90β-KO cells identified a comparatively higher abundance of BCR-ABL1-foci (in the cytoplasmic/nucleocytoplasmic region) in HSP90α-KO cells compared to HSP90β-KO and control cells (Supplemental Fig. 1M). These results are in agreement with a previous study [5], where HSP90β was shown to interact and stabilize BCR-ABL1 kinase with comparatively better potency than HSP90α. As in the case of our HSP90α-KO model, the exclusive expression of the HSP90β isoform resulted in the hyperactivation of BCR-ABL1 and the subsequent activation of downstream pro-survival signaling pathways.

Taken together, the loss of HSP90β isoform in BCR-ABL1 + CML cell lines (K562 and KCL22) leads to an increase in the HSP90α isoform, while loss of HSP90α doesn’t induce changes in HSP90β levels.

### Loss of HSP90α represses in vivo growth of BCR-ABL1 + (K562) leukemia cells

To test possible functional implications on the BCR-ABL1+ leukemia cells’ growth upon loss of either HSP90α/β, we next performed in vitro and in vivo functional assays. CDK4 and CDK6 are well-recognized clients of HSP90β [27] and play a crucial role in cell cycle progression. In line with unchanged CDK4/6 expression (Supplemental Fig. 1I, J), no changes in the cell cycle progression was determined upon HSP90α/β-KO K562 cells (Supplemental Fig. 1N). Next, we performed colony forming unit (CFU) assays in the semi-solid medium (Supplemental Fig. 1O, P). HSP90β-KO (K562 and KCL22) cells formed fewer (*p* < 0.05) and morphologically smaller colonies in comparison to control and HSP90α-KO cells. In comparison, the colonies of HSP90α-KO (K562 and KCL22) cells had a dispersed and atypical phenotype, with slightly higher (not significant) total colony numbers than the respective control cells. Further, the in vivo transplantation efficiency of HSP90α/β-KO (K562) cells in an immunodeficient NSG mouse model was examined. Interestingly, the engraftment capacity of HSP90α-KO cells was significantly (*p* = 0.0002) reduced in comparison to HSP90β-KO or the control group, which was corroborated by the significant (*p* = 0.0015) increase in the overall survival (19 days) of the animals (Fig. 1F–H). The differences between in vitro CFU assay and in vivo growth of HSP90α-KO cells likely appeared due to the absence of eHSP90α upon HSP90α-KO, which is known for promoting invasiveness and metastasis of the malignant cells [33–37], a function not relevant for ex vivo growth.

### HSP90α loss causes downregulation of PTPRC (or CD45) expression in BCR-ABL1 + (K562 and KCL22) cells

**Fig. 2:**
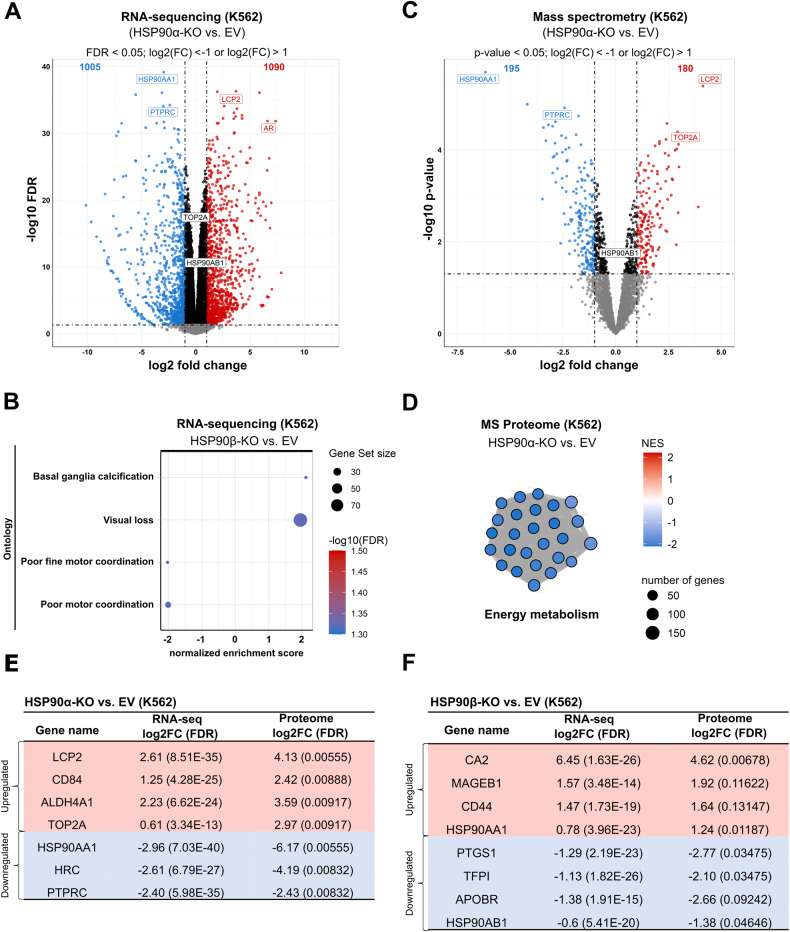
Transcriptomic (RNA-sequencing) and quantitative mass spectrometry (MS)-based proteomic analysis of HSP90α/β-KO cells. **A** Volcano plot showing significantly (FDR <0.05; log2(FC) < −1 or log2(FC) > 1, calculated using edgeR (F-Test & Benjamini-Hochberg correction) up- or down-regulated genes from RNA-sequencing data (obtained from three independent replicates) of HSP90α-KO compared to empty vector (EV) control K562 cells. Black dots represent genes that are not significantly regulated, while gray dots represent significantly regulated genes, but below log2(FC) threshold. Blue and red dots represent significantly downregulated and upregulated genes, respectively. **B** fGSEA on the RNA-seq data of HSP90β-KO cells, displaying significantly (FDR = 0.05) regulated ontology gene set signatures in comparison to EV control. **C** Volcano plot obtained from five independent replicates of HSP90α-KO compared to EV control K562 cells showing up- or down-regulated proteins based on MS-based proteomics data applying *p*-value < 0.05 and log2(FC) < −1 or log2(FC) > 1 as the specificity cutoff criteria. **D** Gene clusters obtained using clusterProfiler on the MS data of HSP90α-KO cells revealed significant downregulation (FDR = 0.05) of energy metabolism signature. Normalized enrichment scores (NES). Tables showing consistently up- or down-regulated genes in HSP90α-KO (**E**) or in HSP90β-KO (**F**) K562 cells from the RNA-seq and MS-based proteomics analysis.

We next utilized multi-omics approaches (including Transcriptomics, Proteomics and Secretomics) to evaluate the potential implications on the distinctive signaling pathways upon loss of HSP90α/β isoforms in K562 cells. Firstly, differential RNA expression analysis using RNA-sequencing (RNA-seq) was performed, which revealed 2095 genes (1090 up- and 1005 down-regulated) with consistent and significant (FDR < 0.05; log2(FC) < −1 or log2(FC) > 1) altered expression in HSP90α-KO cells in comparison to control cells (Fig. 2A, Supplemental Fig. 2A). In contrast, 903 genes (368 up- and 535 down-regulated) were altered in HSP90β-KO cells in comparison to control cells (Supplemental Fig. 2A, B). Fast gene set enrichment analysis (fGSEA) and clusterProfiler revealed significant enrichment in gene sets associated with the leukemic stem cell downregulation, MAPK/ERK signaling and immune cell development and activation in HSP90α-KO cells (Supplemental Fig. 2C, D). Strikingly, enrichment of a gene signature related to visual loss was found enriched in HSP90β-KO cells (Fig. 2B), which can be seen in line with ocular toxicity, a common side effect reported during clinical use of HSP90i [2, 38, 39].

Next, differential protein expression analysis using quantitative mass spectrometry (MS) based proteomic data revealed 375 proteins (180 up- and 195 down-regulated) with consistent and significant (*p*-value < 0.05; log2(FC) < −1 or log2(FC) > 1) altered expression in HSP90α-KO cells (Fig. 2C, Supplemental Fig. 2E), whereas 213 proteins (103 up- and 110 down-regulated) were found in HSP90β-KO cells in comparison to control (Supplemental Fig. 2E, F). Aligned with the previous WB results (Fig. 1C), higher levels of HSP90AA1 (HSP90α) and HSPA1A (HSP70) were identified in the MS data of HSP90β-KO cells (Supplemental Fig. 2F). fGSEA from the MS data identified enrichment in the gene sets involved in the cell cycle, chromosomal and cytoskeleton organization in HSP90α-KO cells (Supplemental Fig. 2G). Notably, both fGSEA and clusterProfiler identified significant downregulation in the oxidative phosphorylation-, cellular respiration- and energy metabolism-related gene signature in the HSP90α-KO cells (Fig. 2D, Supplemental Fig. 2G). The importance of HSP90 in coordinating and supporting a multitude of metabolic pathways necessary for energy generation and efficient cellular respiration has been shown in a previous study [40]. In contrast, there was no enrichment in the gene sets identified (in any of the biological processes) in HSP90β-KO cells compared to control. We then compared the overlap of genes between HSP90α- and HSP90β-KO cells on the mRNA and protein level and found 210 genes (100 up- and 110 down-regulated) shared between HSP90α- and HSP90β-KO cells (Supplemental Fig. 2H). Subsequently, we examined the overlap of some top differentially up- or down-regulated genes from RNA-seq and MS data in the KO cells (Fig. 2E, F).

**Fig. 3:**
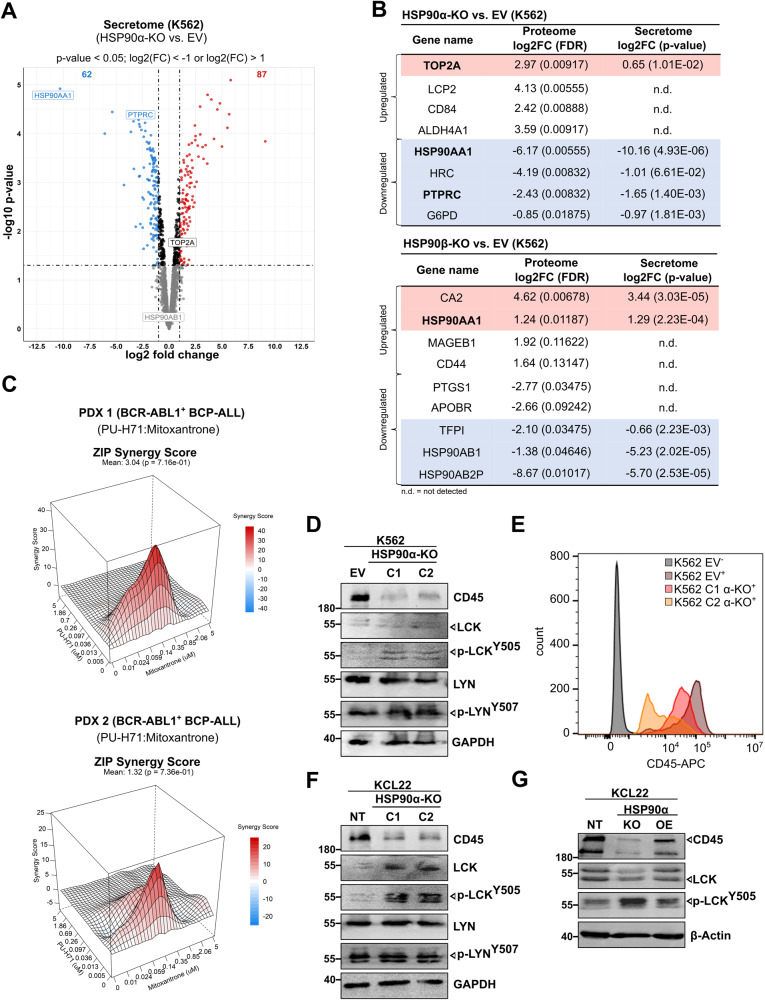
Quantitative MS-based secretome analysis validated PTPRC (CD45) as differentially regulated hit in HSP90α-knockout cells. **A** Volcano plot obtained from five independent replicates of HSP90α-KO compared to EV control K562 cells showing up- or down-regulated proteins based on MS-based secretomics data applying *p*-value < 0.05 and log2(FC) < −1 or log2(FC) > 1 as the specificity cutoff criteria. The third replicate of the EV control in the secretome data was omitted from the statistical analysis due to its significant deviation from the other four replicates. **B** Table showing consistently up- or down-regulated proteins from the MS-based proteomic and secretomic analysis in HSP90α-KO cells (upper panel) and HSP90β-KO K562 cells (lower panel). **C** Synergy maps of PU-H71 and Mitoxantrone combination matrix for two BCR-ABL1 + BCP-ALL patient derived xenograft (PDX) cells, using zero interaction potency (ZIP) method [79]. Visualization was done using SynergyFinder package. WB analysis of CD45 (intracellular domain) and respective inactivating phosphorylation levels of downstream effectors (p-LCK^Y505^ and p-LYN^Y507^) in HSP90α-KO K562 (**D**) and KCL22 (**E**) cells. Glyceraldehyde 3-phosphate dehydrogenase (GAPDH) served as a loading control. **F** Fluorescence antibody staining (using FACS) validated downregulation of PTPRC (CD45) on the surface of K562 HSP90α-KO cells. **G** Rescue experiment was performed, in which HSP90α-KO KCL22 cells were transiently transfected with HSP90α-overexpression (OE) construct to re-express the HSP90α isoform.

HSP90α is secreted extracellularly (eHSP90α), which acts as mediator of tumor cell invasion and metastasis [33–36]. Therefore, we next performed MS-based secretome analysis to evaluate changes in the secreted protein profile in the extracellular space upon loss of HSP90α/β isoform. To identify and quantify the peptides/proteins, the human sequence database from Uni-ProtKB was used, and a total of 2051 protein groups were identified in the K562 cells (FDR = 0.01). Differential protein expression analysis revealed 149 proteins (87 up- and 62 down-regulated) with altered expression in the HSP90α-KO cells compared to the control cells (Fig. 3A). In contrast, 124 proteins (57 up- and 49 down-regulated) were found to be altered in the HSP90β-KO cells in comparison to the control cells (Supplemental Fig. 3A). As expected, secretion of HSP90α was found most significantly downregulated in HSP90α-KO cells, while eHSP90α expression went significantly up in HSP90β-KO cells (Fig. 3B).

Further, we validated some of the top hits shared in the proteogenomic characterization, such as TOP2A, LCP2 (SLP76) and PTPRC (CD45). A consistent upregulation of LCP2 and TOP2A upon HSP90α loss was validated in BCR-ABL1 + CML cell lines (K562 and KLC22) and in BCR-ABL1 + BCP-ALL cell line (SUP-B15) (Supplemental Fig. 3B). Next, as a functional validation step based on high TOP2A levels detected (at proteome and secretome levels) upon HSP90α loss, pharmacological drug screenings were performed by combining TOP2A and HSP90 inhibitors [41, 42]. Indeed, the combination of PU-H71 (HSP90i) along with Mitoxantrone (TOP2i) displayed a significant (ZIP Synergy score ≥ 20) synergistic interaction against two BCR-ABL1 + BCP-ALL (relapsed) patient derived xenograft (PDX) cells and BCR-ABL1 + CML leukemia cell lines (K562 and KCL22) (Fig. 3C, Supplemental Fig. 3C). Notably, in all multi-omics approaches we detected a consistent downregulation of PTPRC (or CD45) expression and secretion upon HSP90α loss. These results were corroborated in HSP90-KO K562 or KCL22 models (Fig. 3D–F). In addition, we evaluated the expression of downstream signaling partners of CD45, specifically LCK and LYN. Notably, there were no discernible changes in LYN and p-LYN^Y507^ expression, whereas the expression of LCK and p-LCK^Y505^ (phosphorylation site known to negatively influence LCK catalytic activity) [43], exhibited an increase following HSP90α-KO. However, no statistical difference in the p-LCK^Y505^/LCK ratio was determined (Supplemental Fig. 3D), suggesting that the elevation in p-LCK^Y505^ expression is due to an overall increase in the LCK expression upon HSP90α-KO. We next performed recovery experiments, where transient re-expression of HSP90α in the HSP90α-KO cells restored CD45 expression (Fig. 3G and Supplemental Fig. 3E).

Enhanced CD45 expression is associated with the increased risk of relapse in B- or T-ALL [44], and the dependency of CD45 expression on HSP90α could serve as a predictive biomarker for evaluating the effectiveness of HSP90α inhibition [45].

### Chronic exposure to HSP90 inhibitor PU-H71 promotes HSP90α overexpression in K562 cells

**Fig. 4:**
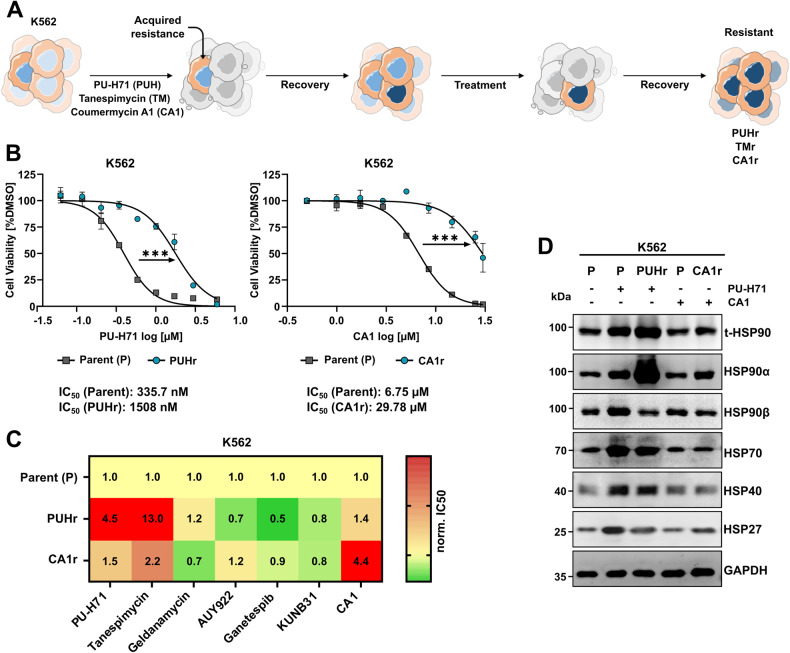
Resistance against HSP90i PU-H71 is attained by HSP90α overexpression. **A** Schematic depiction of the workflow of generating HSP90 inhibitor (HSP90i) resistant cells, through chronic exposure of HSP90-N-terminal domain- (PU-H71 and Tanespimycin or TM) or HSP90-C-terminal domain-targeting (Coumermycin A1 or CA1) inhibitors in K562 cells. **B** Dose–response curves from three independent experiments showing significant (***p ≤ 0.001, unpaired two-tailed student’s t-test) increase in IC_50_ values for PU-H71-resistant (PUHr) and CA1-resistant (CA1r) cells in comparison to their parental (P) counterparts. **C** Cross-resistance of PUHr and CA1r cells to other HSP90i with similar or different modes of action (MoA). The numbers in the heat map indicate the normalized fold-change of the IC_50_ values of the resistant cell lines to the parental counterpart. The red color depicts an increase in IC_50_ value whereas green indicates a decrease in IC_50_ value in comparison to the parental control. **D** WB analysis of PUHr, CA1r and control parental (P) cells after re-treatment with CA1 (2 µM), PU-H71 (500 nM) or vehicle (-) for 24 h. GAPDH served as a loading control.

To better understand resistance mechanisms evoked during pharmacological inhibition of HSP90, we generated HSP90i resistant (K562) cells against the HSP90-N-terminal domain (NTD) targeting inhibitor (PU-H71 and 17-AAG or Tanespimycin) or against HSP90-C-terminal domain (CTD) targeting inhibitor (Coumermycin A1 or CA1). Briefly, the clonal selection was carried out by repetitive treatment cycles using increasing inhibitor concentrations (Fig. 4A). Dose–response curves of PU-H71 resistant (PUHr), Tanespimycin (TM) resistant (TMr) and CA1 resistant (CA1r) clones displayed significant shifts in IC_50_ (4.5, 17.6, and 4.4 fold, respectively) as compared to the parental cells (Fig. 4B, Supplemental Fig. 4A). PUHr, TMr, or CA1r cells also displayed cross-resistance toward other HSP90i with different modes of action (Fig. 4C, Supplemental Fig. 4B). Of note, upon re-treatment with the respective inhibitors, only PUHr cells displayed a strong upregulation of HSP90α in comparison to the parental counterpart (Fig. 4D, Supplemental Fig. 4C). Consequently, the total-HSP90 levels were also found higher in PUHr cells, whereas no changes in the HSR induction was noticed in the PUHr cells compared to respective parental cells. The resistance escape mechanisms against HSP90i are additionally facilitated through the activation of numerous kinases [46]. HSP90 inhibition can lead to the destabilization of the SRC-AKT-ERK kinase axis [47, 48]. In agreement, we also observed upregulation in the total-AKT or -SRC levels, implying an overall stabilization of these proteins by high HSP90α levels (Supplemental Fig. 4D). The elevated total-AKT levels later protected mTOR signaling from PU-H71 re-treatment, confirmed by recovery of the hallmark phosphorylation at the T389 site of the p70S6 kinase (p70S6K), maintaining the phosphorylation of ribosomal protein S6 (RPS6) at position S235/236 (Supplemental Fig. 4D). However, the reported dependency on the p90 RSK and ERK signaling cascade to confer resistance against the HSP90-NTD targeting inhibitor was not verified in our PUHr cells [46]. In contrast, CA1r cells exhibited a notable increase in the phosphorylation of RPS6 at the S240/244 site upon CA1 re-treatment (Supplemental Fig. 4D).

In general, these findings indicate that leukemia cells resistant to HSP90i employ specific adaptations to develop resistance against NTD- or CTD-targeting HSP90i (Supplemental Fig. 4E).

### Prolonged treatment with PUH71 causes genetic alterations in the *HSP90AA1* (HSP90α) gene in K562 cells

**Fig. 5:**
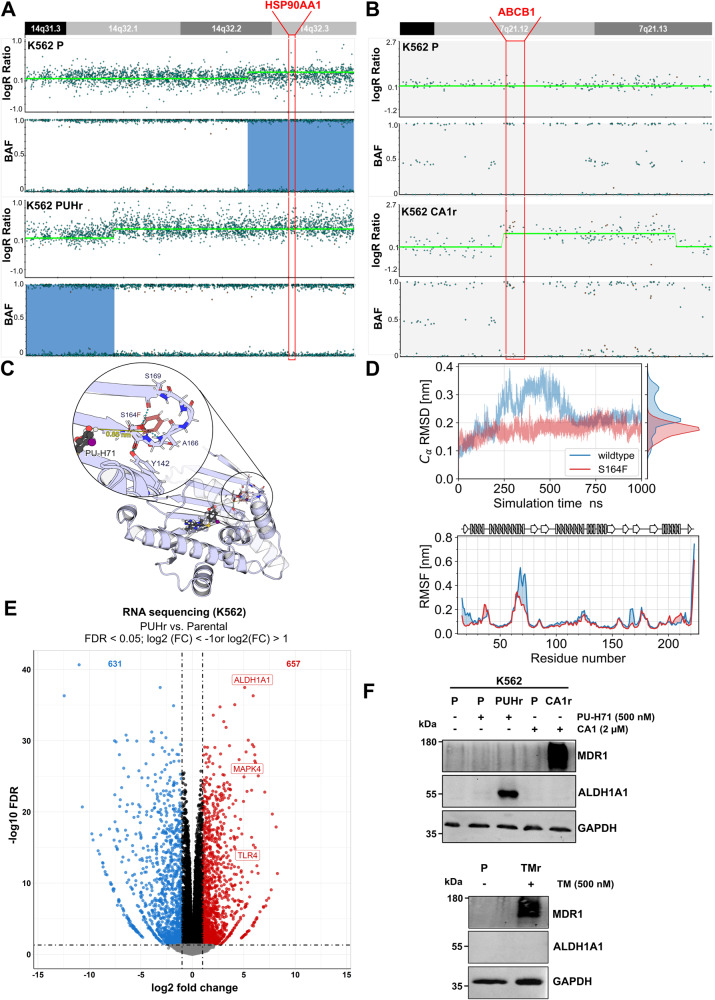
PU-H71 resistant cells acquire copy number gain and mutation (S164F) in the *HSP90AA1* gene and overexpress ALDH1A1. **A** SNP array results of PUHr cells in comparison to the parental (P) counterpart revealed an acquired 15 Mb copy number gain in 14q32.1q32.3 encompassing the *HSP90AA1* gene locus (highlighted in red). The upper panel depicts the log2 ratio and the B-allele frequency (BAF) of the parental cells (K562 P), whereas the lower panel depicts the log2 ratio and the BAF of the resistant PUHr cells. **B** SNP array analysis of CA1r cells revealed an acquired copy number gain in 7q21.12q21.13 encompassing the *ABCB1* gene locus (highlighted in red), which is not present in the K562 parental (P) cells. **C** Structural model of the N-terminal domain of HSP90α bound to PU-H71 (based on PDB ID 2fwz). The substitution site S164F is highlighted in red. **D** Upper panel: Alpha carbon-root mean square deviation (C_α_-RMSD) of wild type and variant (S164F) HSP90α over the course of the simulation. The wildtype HSP90α structure exhibits stronger conformational changes than the variant S164F, and these changes are reversible. Bottom panel: Alpha carbon-root-mean-square fluctuation (C_α_-RMSF) of HSP90α identifies two major regions, which lead to the stronger fluctuations in RMSD observed in the wildtype HSP90α structure. One of these regions is directly adjacent to the mutation site S164F. Above the plot, a schematic representation of the secondary structure is given. **E** Volcano plot of the significantly (FDR<0.05; −1 > log2(FC) > 1, calculated using edgeR (F-Test & Benjamini-Hochberg correction) up- or down-regulated genes in the mRNA expression profile (from RNA-sequencing data) of PUHr vs. parental K562 cells (from three independent replicates). **F** WB analysis of PUHr and CA1r cells (upper panel), Tanespimycin resistant (TMr) cells (lower panel) in comparison to control parental (or P) cells after re-treatment with respective inhibitors or vehicle (-) for 24 h. GAPDH served as a loading control.

To identify whether these changes mentioned above were solely short-term adaptive changes or if there was any underlying genetic cause, we subjected PUHr and CA1r cells to SNP array analysis (Fig. 5A, B). In accordance with elevated HSP90α level, PUHr cells harbored a 15 Mb copy number gain on 14q32.12q32.3, in which the *HSP90AA1* gene is located (Fig. 5A). Moreover, to identify single nucleotide variations (SNVs) and insertion/deletions (indels) during resistance acquirement toward HSP90i (PU-H71 and CA1), whole exome sequencing (WES) of PUHr and CA1r cells was next performed. WES identified 100 and 59 acquired variants in PUHr and CA1r cells, respectively, of which 6 were shared between each. Strikingly, we identified two distinct SNVs in the *HSP90AA1* gene. PUHr cells acquired the missense variant p.(S164F) (chr14:102085796 G > A), whereas the CA1r cells harbored the p.(L29F) (chr14:102086292 C > A) (Supplemental Table 1). Interestingly, both resistant cell lines also acquired distinct variants in the *CLMN* gene, with PUHr harboring a p.(S217N) and CA1r a p.(E588Q) (ENST00000298912.9) missense variant (Supplemental Table 1). Moreover, in CA1r cells SNP array identified 1.9 Mb copy number gain on 7q21.12q21.13, in which the *ABCB1* gene (encoding MDR1) is located (Fig. 5B).

To understand how the S164F substitution affects PU-H71 binding and HSP90α in general, we next modeled the S164F variant in silico and compared it to the wildtype structure (Fig. 5C). The substitution site is located in a solvent-exposed loop which is ~0.9 nm away from the PU-H71 binding site. Thus, the substitution is unlikely to interfere with the binding of the inhibitor directly. However, S164 forms multiple hydrogen bonds including to Y142, such that the substitution may lead to conformational changes in the NTD. To explore this possibility, we performed unbiased molecular dynamics (MD) simulations (1 µs length each) of the wildtype and the S164F protein to which PU-H71 is bound. We observed no difference in the binding mode of PU-H71, i.e., the ligand remained stably bound in both simulations (Supplemental Fig. 5A). Moreover, we did not observe major conformational changes in the variant with respect to the crystal structure. In fact, the S164F variant remained more stable throughout the simulation than the wildtype structure (Fig. 5D). This is likely due to the phenylalanine sidechain forming a hydrophobic core, thereby stabilizing the adjacent loop. While these changes do not affect the stability of bound PU-H71, they supposedly interfere with the binding pathway by introducing additional conformational constraints.

To determine the changes at a transcriptomic level in PUHr cells compared to the parental cells, we next performed RNA-seq analysis. A significant increase in the ALDH1A at mRNA and at protein level was determined in the PUHr cells as compared to CA1r and TMr cells (Fig. 5E, F). Further, fGSEA revealed enrichment in the WNT signaling and growth factor response related gene signatures and downregulation of immune related signatures in the PUHr cells (Supplemental Fig. 5B). In contrast, consistent with earlier studies [49–52] and supported by our SNP array findings (in CA1r cells), both CA1r and TMr cells exhibited elevated MDR1 expression (Fig. 5F).

These findings demonstrate that the HSP90α isoform is a prominent cause of resistance against clinically advanced HSP90i PU-H71. Therefore targeting HSP90α and identifying therapeutic combinations can effectively avert the development of resistance to treatment with HSP90 inhibitors alone.

### CDK7 and HSP90 inhibitors act synergistically against BCR-ABL1+ leukemia cells by reducing heat shock response induction and HSP90α overexpression

**Fig. 6:**
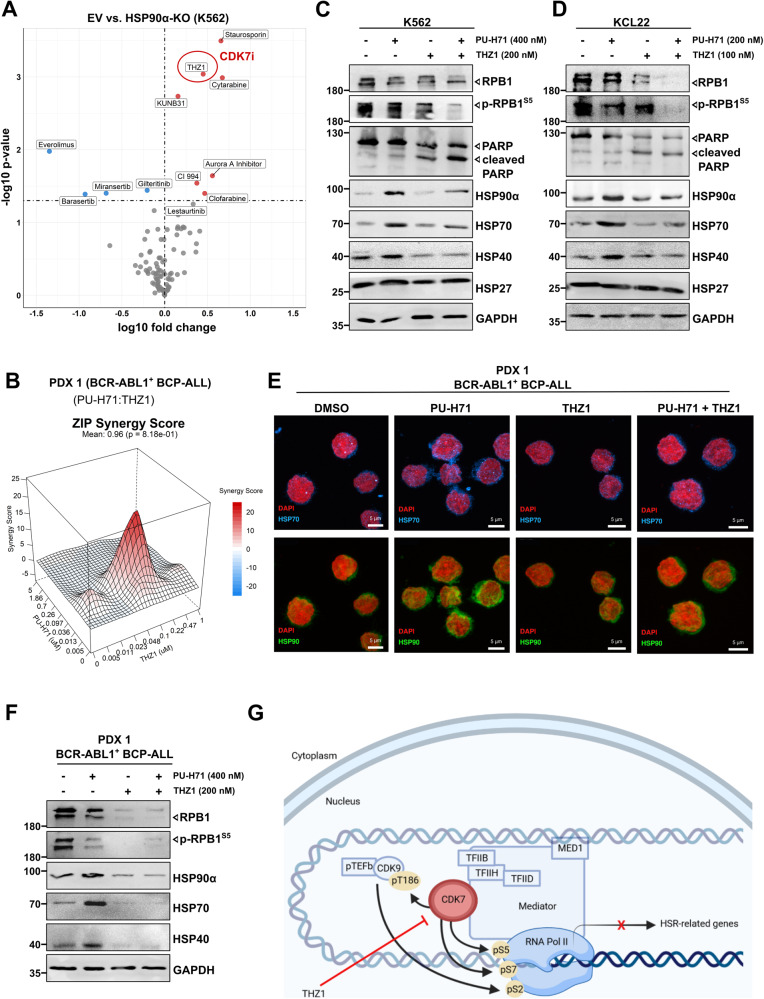
Combinatorial targeting of CDK7 and HSP90α acts synergistically against BCR-ABL1+ leukemia cells *via* blocking heat shock response induction. **A** Comparative cell viability was measured by luminescent-based ATP-Glo assay after screening HSP90α-KO K562 cells on an ex vivo high throughput drug screening platform, including 93 inhibitors. Average IC_50_ values from three independent replicates are depicted in the volcano plots compared to the empty vector (EV) control. Significance was calculated using unpaired t-test. **B** Synergy map of PU-H71 and THZ1 (CDK7i) combination matrix in a BCR-ABL1 + BCP-ALL PDX sample was generated using Zero Interaction Potency (ZIP) method. Visualization was performed using SynergyFinder package. The experiments were reproduced three times and representative synergy map is shown. WB analysis of K562 (**C**) and KCL22 (**D**) cells following (24 h) treatment with PU-H71, THZ1 alone, or in combination (at depicted concentrations). GAPDH served as a loading control. **E** Immunofluorescence imaging also confirmed a reduction in the HSP90 and HSP70 levels (in PDX1 cells) upon treatment with a combination of PU-H71 (200 nM) and THZ1 (100 nM), in contrast to the effects of PU-H71 (200 nM) treatment alone. **F** WB analysis of BCR-ABL1 + BCP-ALL PDX cells after treatment with PU-H71 and THZ1 alone or in combination (at depicted concentrations). GAPDH was used as a loading control. **G** Schematic depiction of CDK7 inhibition *via* THZ1 on the expression of HSR-related genes.

In order to elucidate the actionable therapeutic targets in BCR-ABL1+ leukemia, we next screened HSP90α/β-KO (K562) models on the ex vivo high throughput drug screening platform, consisting of conventional chemotherapeutics and targeted inhibitors (Supplemental Table 2). Of note, HSP90α-KO cells were found hypersensitive toward CDK7i (THZ-1) and standard chemotherapeutics (Cytarabine and Clofarabine) (Fig. 6A). In line, a high CDK7 expression was observed upon loss of HSP90α isoform (Supplemental Fig. 6A). Moreover, in agreement, HSP90β isoform specific inhibitor (KUNB31) [27] was found differentially potent against HSP90α-KO cells. Conversely, HSP90β-KO cells (expressing high levels of HSP90α) displayed hypersensitivity toward several HSP90i (PU-H71, AUY922, BIIB021, and Ganetespib) (Supplemental Fig. 6B). Based on these differential vulnerabilities noticed in HSP90α-KO cells, we next performed combinatorial drug screenings using clinically advanced HSP90i (PU-H71) along with a CDK7i (THZ1). These screenings were carried out utilizing BCR-ABL1 + CML leukemia cell lines (K562 and KCL22) and their TKI-resistant counterparts [4], referred to as K562r, KCL22r, respectively (Supplemental Fig. 6D, E). Furthermore, TKI-resistant BCR-ABL1 + BCP-ALL cells designated as SUPB15r and three (relapsed) PDX cells were included, along with a murine BA/F3 cell line model expressing TKI-resistant BCR-ABL1^T315I^ mutant cells (made resistant to third generation TKI Ponatinib or PN) [4], referred to as BA/F3 BCR-ABL1^T315I-PNr^ cells (Fig. 6B, Supplemental Fig. 6F–H). Notably, in all cases, the combination of PU-H71 along with THZ1 exhibited significant (Synergy score ≥ 15) synergistic interaction. Next, we tested the combination of PUH71 and THZ1 on peripheral blood derived mononuclear cells (PBMCs) obtained from three healthy individuals. Strikingly, we found out that healthy PBMCs are significantly less sensitive to the PUH71 + THZ1 combination in comparison leukemia K562 cells (Supplemental Fig. 6I).

The inhibition of CDK7 (TFIIH subunit of RNA polymerase II or RNAPII) can initiate a series of defects in the initiation, proximal pausing and elongation of RNAPII [53]. Consequently, we noticed a strong synergistic interaction between HSP90- and CDK7-inhibitors, which acted via impeding RNAPII-mediated transcription of pro-survival heat shock response (HSR)-related genes (Fig. 6C–G and Supplemental Fig. 6J). Of note, combined inhibition of both HSP90 and CDK7 also led to the restoration of HSP90α levels to their basal state, in contrast to the use of HSP90i (PU-H71) alone, demonstrating a promising approach for augmenting HSP90i-based therapy in the future.

## Discussion

The clinical response in BCR-ABL1 + BCP-ALL is only short lived, with relapses being driven by mutations in the BCR-ABL1 kinase or activation of independent circuitries [54]. TKIs are unable to eradicate persisting leukemic stem cells and the frequent development of reduced sensitivity to TKIs is also prevalent, thereby amplifying the risk of relapse [55–57]. Novel treatment approaches are therefore needed with the potential to increase treatment-free remission. One attractive strategy is via destabilization of BCR-ABL1 kinase and its related downstream circuitries by targeting HSP90 [4, 7–9]. However, the adverse events such as acquired resistance (through HSR induction) and toxicity associated with the clinical use of HSP90i have thus far halted their widespread clinical approval [2, 16, 58]. To our knowledge, we have shown here for the first time that the loss of HSP90β isoform induces high expression and secretion of HSP90α isoform (eHSP90a) and pro-survival HSR related protein (HSP70), while the converse outcome was not observed upon loss of HSP90α. Interestingly, HSP90α/β-KO cells displayed no changes in the expression of previously reported HSP90-isoform specific client proteins [22, 27–30], which is however in line with a recent study [21], suggesting a compensatory behavior among these cytosolic isoforms. However, our multi-omics profiling of HSP90α- vs. HSP90β-KO cells revealed overall prominent differences in the regulated signaling pathways, likely due to the diverse adaptions acquired by the cells to compensate for the loss [20, 24]. The tendency of HSP90α isoform to dimerize more frequently than HSP90β, which is required for the proper functioning of HSP90 [59], additionally outlines the differences observed in the regulated pathways. The HSP90β isoform is generally linked with long-term cellular adaptation and early embryonic development, whereas HSP90α is a fast-reactive and stress-inducible isoform [20]. In line, we observed fewer genes and respective pathways altered upon HSP90β-KO than HSP90α-KO [20, 24]. Our multi-omics analysis and rescue experiments consistently identified a strong downregulation of PTPRC (CD45) expression upon loss of HSP90α isoform, affecting downstream p-LCK^Y505^ and LCK expression [43, 48]. Interestingly, CD45 expression correlates positively with BCR-ABL1-induced malignant transformation and negatively with the efficacy of TKI treatment in individuals with CML [60]. In agreement, we noticed a reduction in the in vivo engraftment of BCR-ABL1+ leukemia cells upon HSP90α-KO. Elevated levels of HSP90 have been linked to a dismal prognosis in AML [12], whereas notably, heightened expression of HSP90α isoform is reported in acute leukemia cells and among untreated samples from leukemia patients [61–63]. Our secretome analysis revealed a significant decrease in eHSP90a expression in the HSP90α-KO cells, potentially leading to the suppression of in vivo migration and invasion capabilities of these leukemia cells [33–37].

As previously shown [51], we also observed that the resistance toward PU-H71 conferred cross-resistance only toward TM but not against other tested HSP90i. In our MD simulation analysis, the S164F substitution did not directly affect bound PU-H71; but changed the conformational dynamics of the HSP90-NTD, which may hinder the binding pathway. Interestingly, a missense variant at Y142, which is the hydrogen-bond interaction partner of S164, as well as a CN gain of *HSP90AA1* has been reported in PU-H71-resistant lung cancer cells [51]. Moreover, upregulation of ALDH1A1 was also detected in PUHr cells. Elevated ALDH1A1 levels are often associated with reduced responsiveness to therapy in other malignancies [64, 65]. In contrast, resistance acquired against HSP90i TM and CA1 is primarily mediated through amplification of the *ABCB1* locus and MDR1 efflux pump overexpression [49–52].

Heat shock causes an overall reduction of RNA polymerase II (RNAPII) occupancy across several genes, whereas its occupancy increases at specific pro-survival genes to minimize cellular stress during heat shock [66]. Of these pro-survival genes, HSP70 genes are actively transcribed utilizing a transcriptional mechanism called RNAPII promoter-proximal pausing [67]. In most cases, the exposure of pan- or HSP90-NTD targeting HSP90 inhibitors induces the expression of HSR related proteins (e.g., HSP70), which eventually weakens their cytotoxic effects [2, 4, 8, 16]. Employing high throughput and combinatorial drug screenings, we observed a strong synergism between HSP90 and CDK7 inhibitors, which acts via impeding RNAPII-assisted transcription [53] of pro-survival HSR-related genes and HSP90α. Interestingly, inhibitors of RNAPII has been shown to specifically target dormant leukemia cells [68]. What’s more, we observed a robust synergistic effect between PU-H71 (HSP90 inhibitor) and Mitoxantrone (TOP2 inhibitor) (TOP2i), presumably also operated by inhibition of RNAPII-assisted transcription of pro-survival HSR-related genes and HSP90α by Mitoxantrone [69]. Altogether, combining HSP90 and CDK7 targeting inhibitors can serve as a promising therapeutic combination by mitigating HSP90i-related resistance against therapy refractory leukemia.

## Materials and methods

### Cell culture

BCR-ABL1+ chronic myeloid leukemia (CML) cell lines K562, KCL22 and B-cell precursor acute lymphoblastic leukemia (BCP-ALL) cell line SUPB15 (DSMZ, Braunschweig, Germany) were cultured in RPMI1640 GlutaMAX (Gibco, Thermo Fisher Scientific, Waltham, MA, USA) supplemented with 10–15% FCS and 1% penicillin/streptomycin (Sigma-Aldrich, St. Louis, MO, USA). A regular cell line authentication by short tandem repeat (STR) profiling and mycoplasma testing was performed.

### si- or shRNA-mediated knockdown (KD) and CRISPR-Cas9 mediated knockout (KO) of HSP90α/β isoforms

siRNA pools (Accell SMARTPOOL) or tetracycline (Tet)-inducible microRNA-based lentiviral shRNA vectors (Horizon Discovery, Waterbeach, UK) were used for conditional knockdown of HSP90α/β isoforms. Guide RNAs (gRNAs) targeting *HSP90AA1* (HSP90α) or *HSP90AB1* (HSP90β) were either cloned into the lentiviral expression plasmid (in case of K562) or transfected using Alt-R CRISPR-Cas9 nuclease based system in case of KCL22 and SUP-B15 cells (IDT, Coralville, IA, USA). See supplemental methods for sequences and more details.

### Immunofluorescence (IF) staining

IF stainings were performed as described earlier [70]. Briefly, the Lab-Tek II chamber slides (Thermo Fisher Scientific) were coated with a 50 µg/ml solution of Poly-D-Lysine or PDL (Thermo Fisher Scientific) and incubated for 24 h at 37 °C. For permeabilization, 0.1% Triton X-100 was used, followed by blocking with 10% goat serum (Sigma-Aldrich). Primary antibodies including, anti-BCR-ABL1 (#ab187831, 1:200, Abcam), anti-HSP70 (#4872, 1:200, Cell Signaling Technology Danvers, MA, USA) or anti-total HSP90 (#sc-69703, 1:100, Santa Cruz Biotechnology, Dallas, TX, USA) were used, followed by labeling with Alexa Flour 488 or 594 conjugated secondary antibody (Thermo Fisher Scientific). Antibody stained cells were embedded in ProLong Gold Antifade Mountant (Thermo Fisher Scientific) with DAPI (hydrochloride) (StemCell Technologies, Vancouver, Canada). Confocal laser scanning microscope (Fluoview3000, Olympus) with super apochromatic UPLSAPO 60X objective (Olympus) was used for imaging (at room temperature). FV31S-SW (Ver. 2.6.1.243) viewer software, along with the Omero image server were used to process and develop confocal images. Maximum intensity projections of data were generated directly within the Omero-figure plugin. Signal quantification was performed using Fiji software. The process involved loading images, splitting composite images into individual channels, and creating a Z-projection from all slices with maximum intensity. Subsequently, the images were converted to grayscale by changing the current look-up table (LUT) to grayscale. Square regions were then placed around the cells, and the mean gray values were measured. To ensure accuracy, background values were subtracted from all measurements.

### Murine xenograft transplantation

Luciferase-GFP-positive control, HSP90α- or HSP90β-KO K562 (2.5 × 10^6^ cells) cells were transplanted via intravenous (i.v.) tail injection in 8–12-week-old female *NOD.Cg-Prkdc*^*scid*^ *Il2rg*^*tm1Wjl*^*/SzJ* (NSG) mice (*n* = 5 mice/group) (The Jackson Laboratory) [4]. Mice were be housed in sterile conditions using high-efficiency particulate arrestance filtered micro-isolators and fed with irradiated food and acidified water. The engraftment of the leukemia cells in the animals was monitored by measuring luminescence after i.p. injection of 150 µg per 100 µl D-Luciferin firefly sodium salt monohydrate (Biosynth, Staad, Switzerland), using the Caliper IVIS Lumina II Multispectral Imaging System and the Living Image Software (Perkin Elmer, Waltham, MA, USA). No blinding or randomization was performed.

In house BCR-ABL1 + BCP-ALL cells derived from the peripheral blood (PB) or bone marrow (BM) of three relapsed (TKI-resistant) patients after obtaining informed consent in accordance with the Declaration of Helsinki. The experiments were approved by the ethics committee of the medical faculty of the Heinrich Heine University (Study Nr.: 2019-566). Patient samples were transplanted intravenously in 8–12-week-old female NSG mice. The transplanted (≥90% human) leukemia cells obtained from BM and spleen of the mice were used to perform short-term ex vivo drug sensitivity assay. All animal experiments were conducted in accordance with the regulatory guidelines of the official committee at LANUV (Akt. 81-02.04.2017.A441), under the authorization of the animal research institute (ZETT) at the Heinrich Heine University Düsseldorf.

### RNA-sequencing (RNA-seq)

RNA-seq was performed as described previously [71]. Briefly, RNA was isolated utilizing the Maxwell® RSC simplyRNA cells kit (Promega, Madison, WI, USA, #AS1390). Library preparation was carried out following supplier’s guidelines, using the VAHTs Stranded mRNA-Seq Library Prep Kit (Illumina, San Diego, CA, USA). Total RNA (500 ng) was used for capturing of mRNA, fragmentation, cDNA synthesis, ligation of the adapters and library amplification. Purified libraries were normalized and sequenced on the NextSeq550 (Illumina) with 1 × 76 bp read setup. Followed by using bcl2fastq2 tool to convert the bcl files to fastq files.The raw sequencing data was uploaded to galaxy and an initial quality control was performed by FastQC and aggregated via MultiQC. After cutting the adapters with FASTQ Trimmer, the reads were aligned to the reference genome GRCh38 with RNA STAR. FastQC determined that at least 85% of all reads were uniquely mapped. In order to quantify the gene expression featureCounts was used, followed by edgeR to normalize the data to the sequencing depth. Differentially expressed genes were determined by an absolute log2 fold change of >1/ < −1 and a FDR < 0.05. Differentially expressed genes with a low log2CPM (normalized log2CPM < −1) were treated preferentially.

### Mass spectrometry (MS) based proteome and secretome analysis

Quantitative MS based proteome analysis was essentially performed as described previously [70]. For secretome analysis, K562 cells (EV, HSP90α-KO (C1), or HSP90β-KO (C1); five biological replicates) were washed three times with PBS and FCS-free medium. Later the cells were incubated for 24 h in FCS-free medium at a density of 1 million cells/mL. The conditioned medium was collected by centrifugation (5 min, 800 × *g*, 4 °C) and filtering through a 0.2 µm membrane (Acrodisc 32 mm Syringe Filter with 0.2 µm Supor Membrane; Pall, #4652). Aliquots were shock frozen in liquid nitrogen and stored at −80 °C. See supplemental methods for more details.

### Gene set enrichment analysis (GSEA)

Volcano plots were generated with ggplot2. GSEA was performed with the fGSEA package and all nine major gene set collections of the molecular signature database. The gene ontology GSEA and the enrichment maps were generated by clusterProfiler.

### Cell cycle

Nicoletti method (with Propidium Iodide staining) was used to measure the cell cycle of HSP90α/β-KO K562 cells. To this end, cells (500,000 cells/mL) were seeded onto a 6-well plate and treated with Vorinostat (3 µM) or DMSO. Cells were incubated for 48 h, counted and centrifuged. The pellet was resolved in Nicoletti buffer and cells were transferred to a 96-well plate, incubated for 15 min at room temperature and the DNA content was measure *via* flow cytometry.

### Colony forming unit (CFU) assay

HSP90α/β-KO cells (K562 or KCL22) were seeded (50 cells/mL) in methylcellulose based medium (#H4100, STEMCELL Technologies). After 8 days, the colonies (*n* = 5) were counted and pictures were taken, as described previously [4].

### Caspase 3/7 Glo assay

To measure the Caspase 3/7 activity, the luminescent Caspase-Glo® assay system (Promega, #G8090) was used. Peripheral blood derived mononuclear cells (PBMCs) (100.000 cells/mL) were seeded into a white 96-well plate and treated with PU-H71, THZ1 and with both inhibitors together with the indicated concentrations. Cells were incubated for 24 h and diluted with Caspase-Glo® 3/7 Reagent 1:1 (Caspase-Glo® 3/7 Substrate + Caspase-Glo® buffer was previously mixed according to manufacturer’s instructions). The plate was incubated for 30 min at room temperature and luminescence was measured with the Tecan Spark.

### Generation of HSP90 inhibitor-resistant cells

K562 cells were long-term treated with half of the IC_50_ concentration of PU-H71, Coumermycin A1 (CA1) and Tanespimycin (TM). The clonal evolution was reiterated with 10% increased inhibitor concentration over the course of 12–14 months (Fig. 4A). The individual resistant clones were picked using methylcellulose based medium (#H4100, STEMCELL Technologies). To account for the effect of long-term culture and solvent (DMSO) exposure, parental (P) clone was also treated with same concentration of DMSO and grown in parallel.

### Single nucleotide polymorphism (SNP) array

Copy number analyses were performed using DNA from PUHr and CA1r cells using the CytoSNP-12 v2.1 array (Illumina) encompassing 299,140 SNP markers and were compared to the parental (P) K562 cells. Beeline 2.0.3.3 software was used to convert idat to gct files. Data were processed and analyzed using the BlueFuse Multi 4.5 software from Illumina. Partek Flow was used to identify chromosomal imbalances in resistant cells compared to parental cells. The human reference genome was GRCh38/hg38.

### Whole exome sequencing (WES)

WES of PUHr and CA1r cells along with parental K562 cells was carried out as described before [72], with some modifications. Next-generation WES was performed using the Sure Select Human All Exon V7 kit (Agilent, Santa Clara, CA, USA). The library was paired-end sequenced on an Illumina NextSeq550 (2 × 150 bp) sequencer to yield an average on-target coverage of a minimum 100x. Sanger sequencing was performed to validate the herein-reported variants. See supplemental methods for more details.

### Molecular dynamics (MD) simulation

The protein structure of human HSP90α bound to PU-H71 (2FWZ) was obtained from Protein Data Bank (PDB ID: 2fwz) [73, 74]. Missing atoms were added using the ‘build’ function in the PyMOL Molecular Graphics System (Version 2.1.0: Schrödinger, LLC) [75]. To generate the variant structure, the S164F substitution was introduced with the mutagenesis wizard in PyMOL, selecting the highest probability rotamer. See supplemental methods for more details.

### Western blotting (WB)

Conventional WB and capillary based immunoassay (JESS, Bio-Techne, Minneaspolis, MN, USA) was performed as previously described [4, 70]. Refer supplemental methods for more details, including list of the antibodies and their concentrations used in conventional WB or during JESS. See supplementary material for uncropped western blot images.

### Ex vivo high throughput drug screening (HTDS)

A library containing 93 compounds was created for ex vivo HTDS of leukemic cell lines and patient samples [76]. DMSO dissolved compound library was purchased from Selleck Chemicals and MedChem Express. The compound selection involved the majority of FDA/EMA-approved routinely used chemotherapeutics and targeted drugs involved in the leukemia treatment protocols and inhibitors in the early to late clinical trial phase (see Supplemental Table S2 for the detailed list of drugs). Briefly, the DMSO dissolved compound library was dispensed with increasing concentrations of the inhibitors in 6 dilution steps (0.008–25 μM) on a white 384-well plate (Corning, New York, USA) using digital dispenser (D300e, Tecan, Maennedorf, Switzerland), ensuring precise and robotic compound application in randomized fashion. The cells (≥90% viability) were seeded on the thawed pre-dispensed inhibitor plates using an automated Multidrop Combi Reagent Dispenser (Thermo Fisher Scientific). Differential responses were monitored with ATP-dependent CellTiter-Glo Luminescent viability assay (Promega) after 72 h of inhibitor exposure using a microplate reader Spark 10 M (Tecan). Dose–response curves for the inhibitors were determined by plotting raw data (normalized to controls) with non-linear regression (log(inhibitor) vs. normalized response) variable slope function (*n* = 3 replicates). For combinatorial drug screening, respective inhibitors were printed on white 384-well plates with increasing concentrations in dose–response 8 × 8 matrices. The synergy score calculations were based on the ZIP reference model [77].

### Replicates and statistical analysis

The experiments were reproduced a minimum of three times and representative data are shown. Error bar represent standard deviation (SD). Statistical analyses were conducted using Prism v8.0.2 (GraphPad Software, La Jolla, CA, USA) or using R. Statistical significance was considered for *p* values < 0.05 (*p < 0.05), < 0.01 (**p < 0.01), and <0.001 (***p < 0.001).

### Supplementary information

Supplemental Information

Uncropped western blots

Reproducibility Checklist

## Data Availability

RNA-seq data have been deposited in the NCBI GEO database with the accession ID: GSE208005. The mass spectrometry based proteomics or secretomics raw and expression data have been deposited to the ProteomeXchange Consortium via the PRIDE [78] partner repository with the dataset identifier PXD041871. SNP array and WES data have been deposited in the EGA database with accession ID EGAS00001006385 and EGAS00001006381, respectively.
