# Supplementary material for: Co-targeting HSP90 alpha and CDK7 overcomes resistance against HSP90 inhibitors in BCR-ABL1+ leukemia cells
Source: Cell Death Dis. 2023 Dec 6;14(12):799. doi: 10.1038/s41419-023-06337-3 (PMC10700369; doi:10.1038/s41419-023-06337-3)
Supplement: Supplementary file 1 — Supplemental Information [file 41419_2023_6337_MOESM1_ESM.pdf]

Supplemental Information

Co-targeting HSP90 alpha and CDK7 overcomes resistance against HSP90 inhibitors in BCR-ABL1+ leukemia cells

Melina Vogt,[1†] Niklas Dienstbier,[1†] Julian Schliehe-Diecks,[1] Katerina Scharov,[1] Jia-Wey Tu,[1] Philip Gebing,[1] Julian Hogenkamp,[1] Silke Furlan,[1] Daniel Picard,[1,2] Marc Remke,[1,2] Layal Yasin,[1] David Bickel,[3,4] Munishikha Kalia,[5,6] Alfredo Iacoangeli,[5,6,7] Thomas Lenz,[8] Kai Stühler,[9] Aleksandra A. Pandyra,[1,2] Julia Hauer,[10] Ute Fischer,[1,2] Rabea Wagener,[1,2] Arndt Borkhardt[1,2], Sanil Bhatia[1,2*]

[1]Department of Pediatric Oncology, Hematology and Clinical Immunology, Medical Faculty, Heinrich Heine University Düsseldorf, Düsseldorf, Germany. [2]German Cancer Consortium (DKTK), partner site Essen/Düsseldorf, Düsseldorf, Germany. [3]Interuniversity Institute of Bioinformatics in Brussels, ULB-VUB, Brussels, Belgium. [4]Structural Biology Brussels, Vrije Universiteit Brussel, Brussels, Belgium. [5]Department of Biostatistics and Health Informatics, King's College London, London, UK. [6]Department of Basic and Clinical Neuroscience, King's College London, Maurice Wohl Clinical Neuroscience Institute, London, UK. [7]National Institute for Health Research Biomedical Research Centre and Dementia Unit at South London and Maudsley NHS Foundation Trust and King's College London, London, UK. [8]Molecular Proteomics Laboratory, Biological Medical Research Center, Heinrich-Heine-University Düsseldorf, Düsseldorf, Germany. [9]Institute for Molecular Medicine, Proteome Research, University Hospital and Medical Faculty, Heinrich-Heine-University Düsseldorf, Düsseldorf, Germany. [10]Department of Pediatrics, Children's Cancer Research Center, Children's Clinic Munich Schwabing, School of Medicine, Technical University of Munich, Munich, Germany.

[†] contributed equally to this work

Running Title: Co-targeting HSP90α and CDK7 overcomes resistance

***Corresponding author**: Sanil Bhatia, Department of Pediatric Oncology, Hematology and Clinical Immunology, Heinrich Heine University Düsseldorf, Germany, Moorenstraße 5, Düsseldorf, 40225, Germany. Phone (+49) 211 81 04896; Fax; (+49) 211 81 16436 Email: sanil.bhatia@med.uni-duesseldorf.de

Table of Contents

1. **Supplemental Figures (1-6) and Tables (1-2) 3-18**

2. **Supplemental Materials and Methods 19-26**

3. **Supplemental References 27-28**

1. Supplemental Figures:

Fig. S1

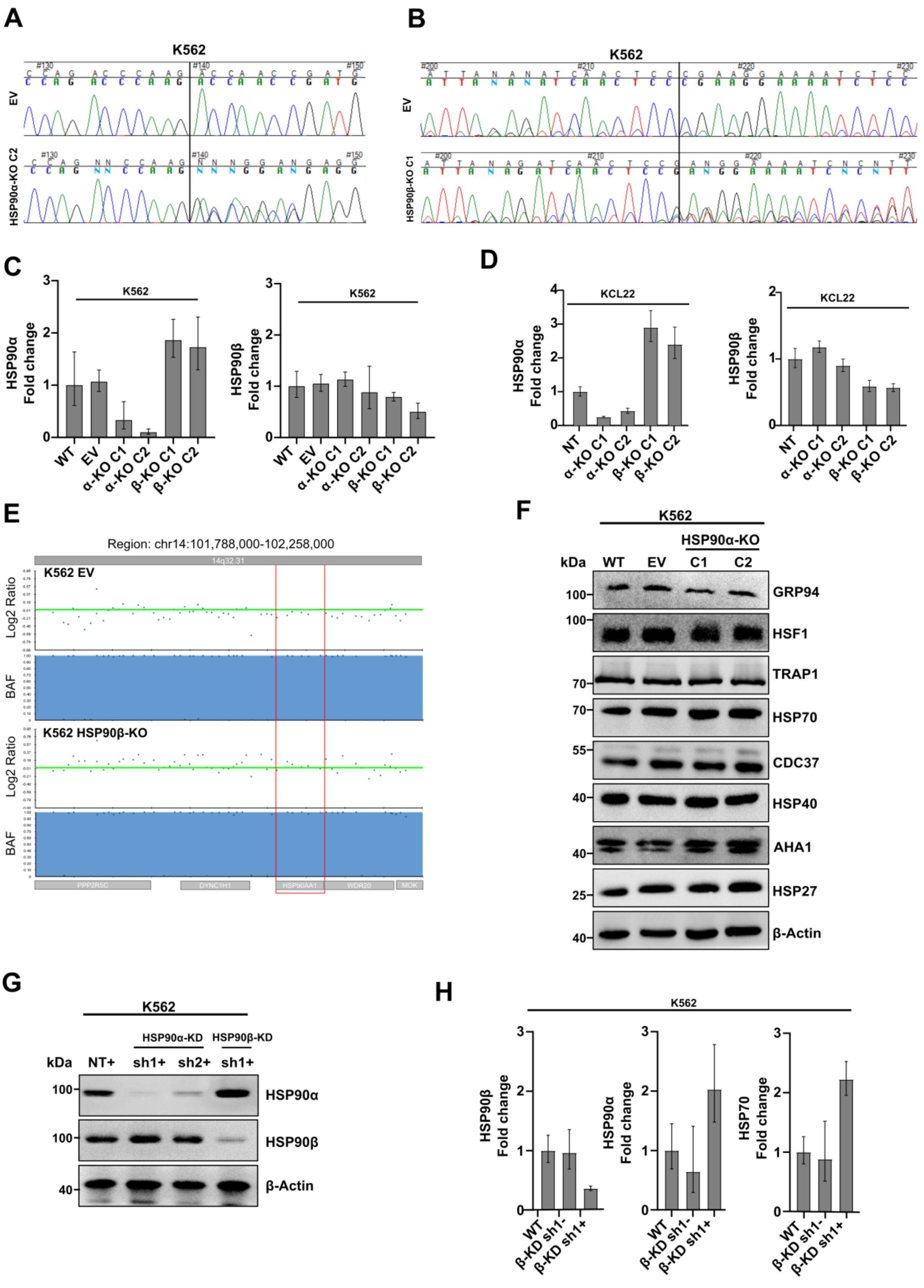

Fig. S1 continued

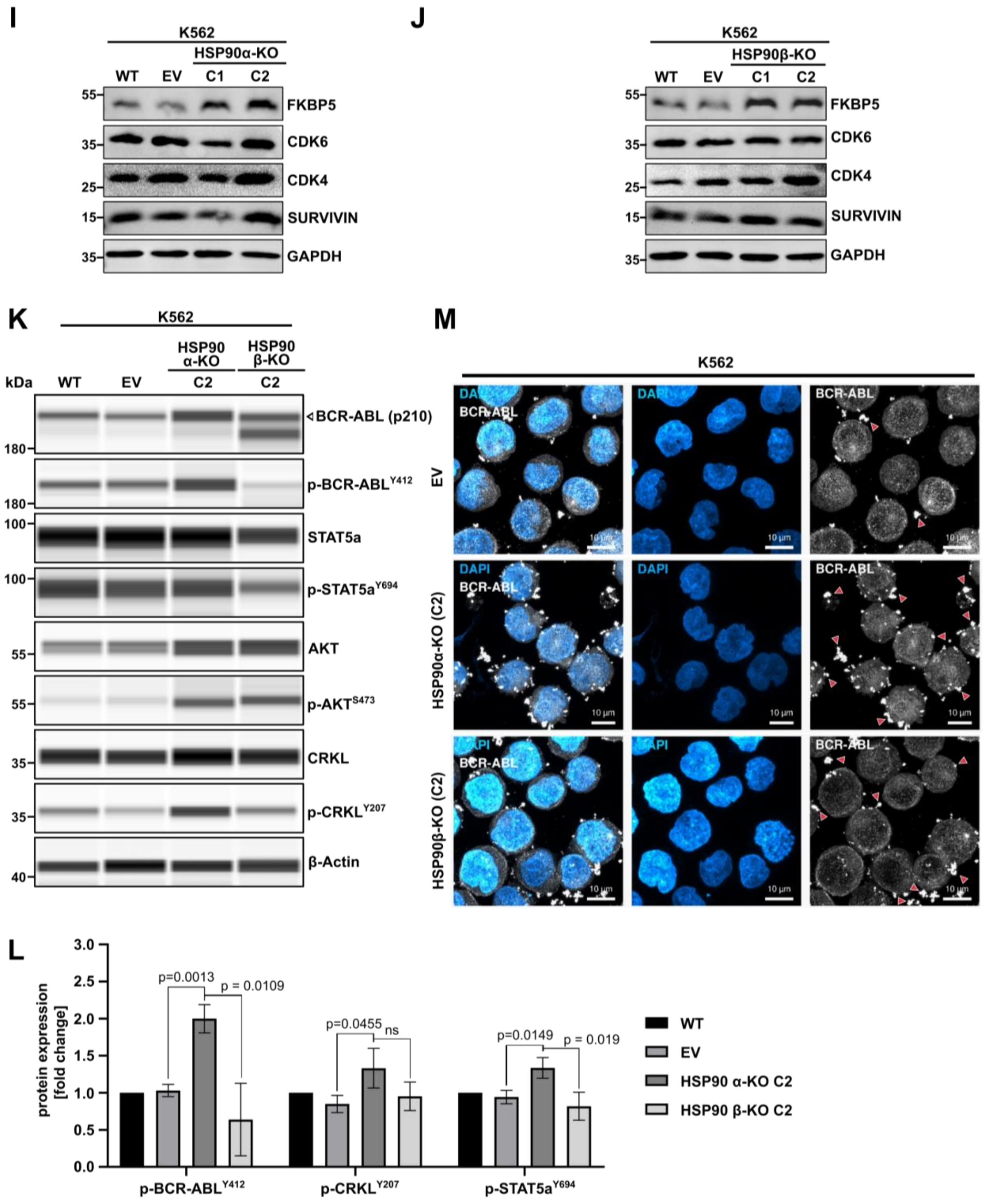

Fig. S1 continued

N

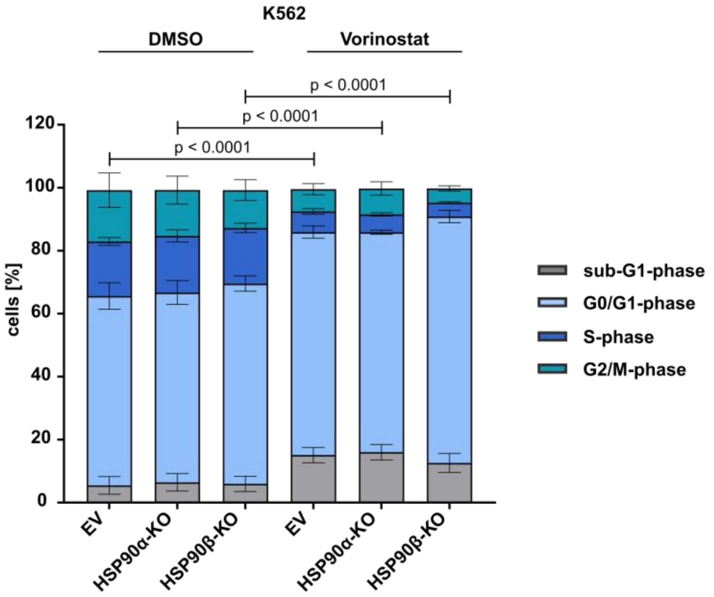

O

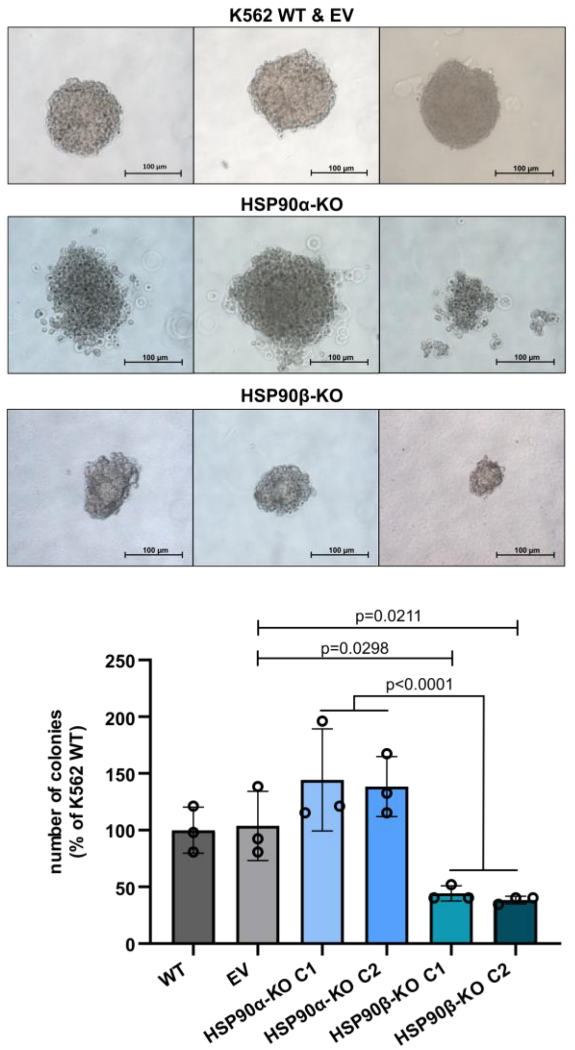

P

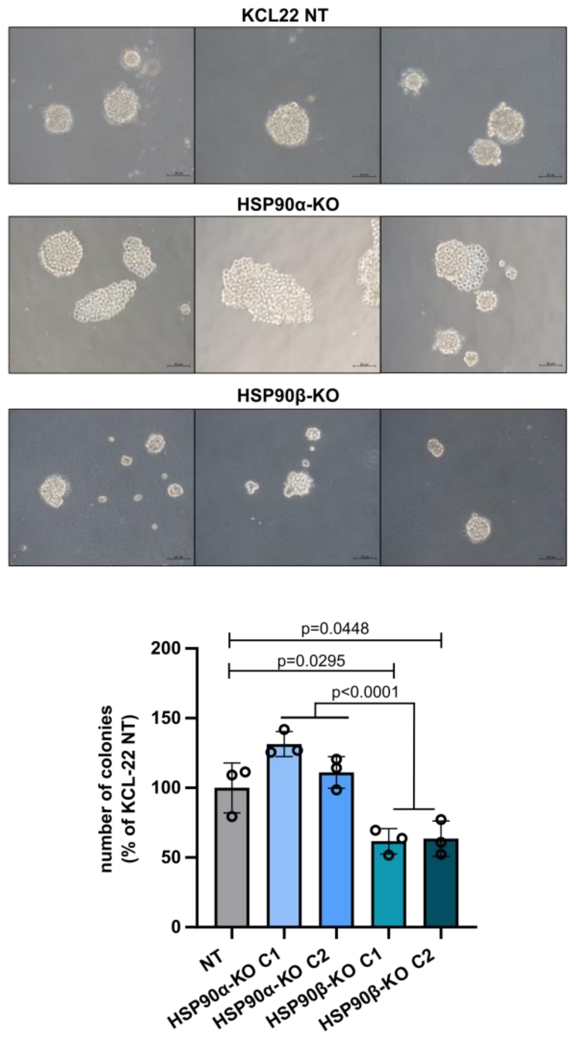

Supplemental Figure 1: Knockdown (KD) or knockout (KO) models of HSP90α/β isoform and testing HSP90α- and HSP90β-KO (K562 and KCL22) cells in functional *ex vivo* assays.

Representative images of Sanger sequencing results of CRISPR-Cas9 mediated HSP90α- (**A**) and HSP90β-KO (**B**) clones. The vertical line indicates the mutation start site. Clone (C), empty vector (EV), wild type (WT) and non-targeting (NT) control. RT-PCR data confirming a notable increase in HSP90α mRNA transcripts upon HSP90β-KO in the K562 (**C**) or KCL22 (**D**) cell line models. (**E**) SNP-array comparing EV control and HSP90β-KO K562 cells. (**F**) Western blotting (WB) analysis of other non-cytosolic HSP90 paralogues (GRP94 and TRAP1), HSR-related proteins (HSP70, HSP40 and HSP27) and HSP90 co-chaperones (AHA1 and CDC37) revealed no apparent change in HSP90α-KO cells. β-actin served as a loading control. (**G**) WB results of the conditional short hairpin RNA (sh) mediated KD of HSP90α (α-KD) and HSP90β (β-KD) isoforms using doxycycline inducible Tet-Off system in K562 cells. Non-targeting control (NT), with (+) or without (-) Doxycycline. β-actin served as a loading control. (**H**) RT-PCR data validating the conditional HSP90β-KD (left panel) at mRNA level, and showing a notable increase in the HSP90α (middle panel) and HSP70 (right panel) mRNA transcripts upon conditional HSP90β-KD. WB analysis of HSP90α-KO (**I**) and HSP90β-KO (**J**) models to show effect on the binding preferences of specific client proteins on distinct HSP90 isoforms (with SURVIVIN as an HSP90α-dependent client and CDK4 and CDK6 as HSP90β- dependent client proteins). FKBP5 expression was found upregulated in both HSP90α/β-KO cells. GAPDH served as a loading control. (**K**) Expression of BCR-ABL and p-BCR-ABL^{Y412} and their related downstream pro-survival effectors (p-CRKL^{Y207} and p-STAT5a^{Y694}) in HSP90α/β-KO cells, analysed by automated JESS WB. β-actin served as a loading control. (**L**) Bars show average protein quantification measurements of p-BCR-ABL^{Y412}, p-CRKL^{Y207} and p-STAT5a^{Y694} levels in HSP90α/β-KO cells compared to EV control (K562) cells. Error bars = SD of three independent replicates; p-values were calculated by unpaired two-tailed student's t-test. (**M**) Immunofluorescence imaging demonstrated a higher abundance of BCR-ABL foci (cytoplasmic/nucleocytoplasmic region) in HSP90α-KO cells than in HSP90β-KO or EV control cells. (**N**) Cell cycle analysis of HSP90α/β-KO cells revealed no significant changes in sub-G1, S, G0/G1 and G2/M phases when treated with vehicle (DMSO) or the positive control vorinostat (SAHA). As anticipated, the administration of the positive control, vorinostat, induced significant changes (determined by two way ANOVA test, n=3) in all cell cycle phases, when compared to the vehicle (DMSO) treatment group. Displayed are representative images of colonies taken from the colony forming unit (CFU) assay after plating K562- (**O**, upper panel) or KCL22- (**P**, upper panel) HSP90α/β-KO cells. A reduced colony sizes compared to the respective controls were observed by HSP90β-KO cells, whereas colonies of the HSP90α-KO cells were loosely packed with atypical morphology. Bar graphs illustrating the results of the CFU assay indicate a growth disadvantage to HSP90β-KO K562 (**O,** lower panel) and to KCL22 (**P,** lower panel) cells in comparison to HSP90α-KO counterparts or respective controls, evidenced by the significant decrease in total colony numbers (n=3, unpaired two-tailed student's t-test).

Fig. S2

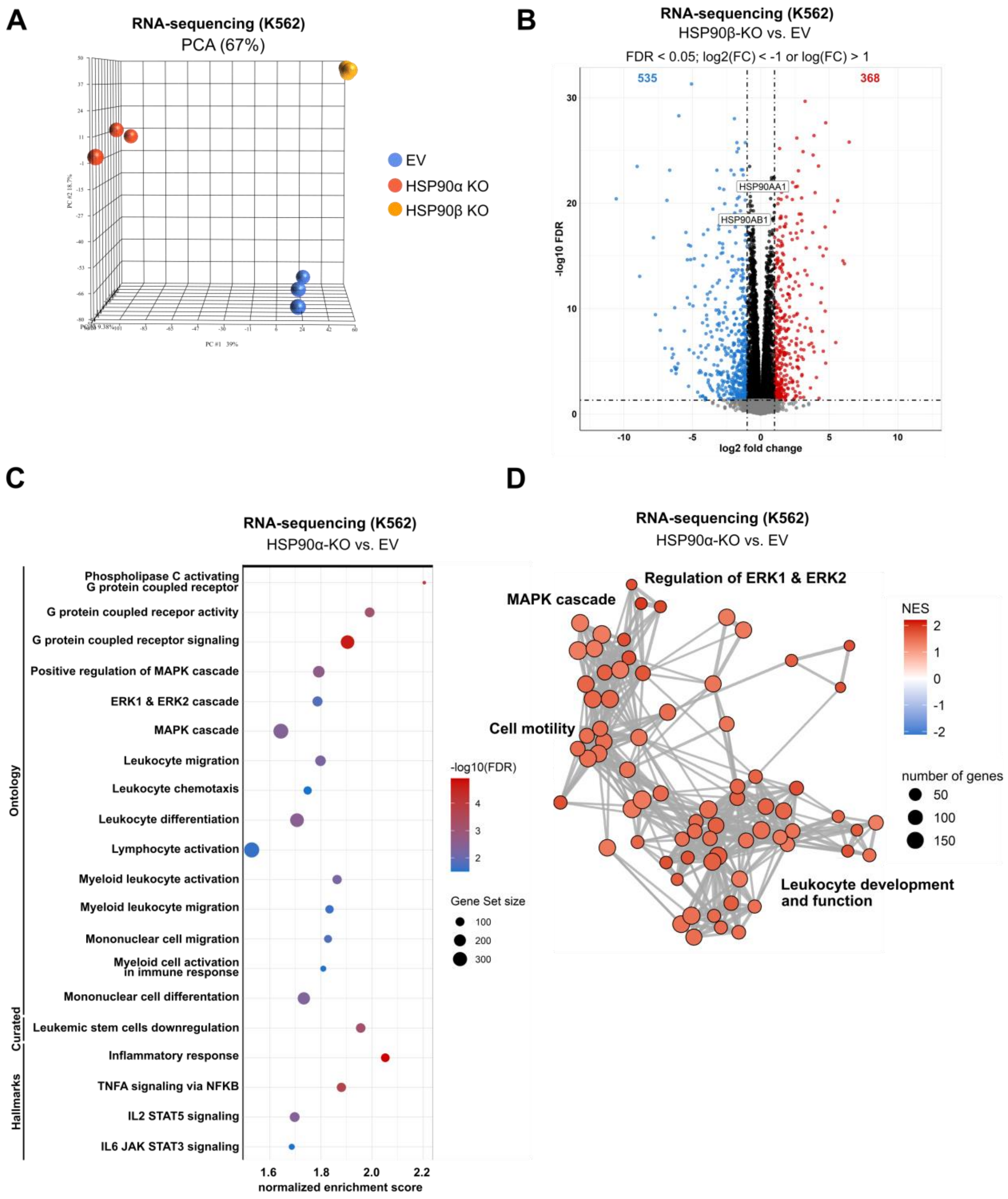
A
RNA-sequencing (K562)
PCA (67%)
EV
HSP90α KO
HSP90β KO
B
RNA-sequencing (K562)
HSP90β-KO vs. EV
FDR < 0.05; log2(FC) < -1 or log(FC) > 1
535
368
HSP90AA1
HSP90AB1
-log10 FDR
log2 fold change
C
RNA-sequencing (K562)
HSP90α-KO vs. EV
Ontology
Curated
Hallmarks
Phospholipase C activating G protein coupled receptor
G protein coupled recepor activity
G protein coupled receptor signaling
Positive regulation of MAPK cascade
ERK1 & ERK2 cascade
MAPK cascade
Leukocyte migration
Leukocyte chemotaxis
Leukocyte differentiation
Lymphocyte activation
Myeloid leukocyte activation
Myeloid leukocyte migration
Mononuclear cell migration
Myeloid cell activation in immune response
Mononuclear cell differentiation
Leukemic stem cells downregulation
Inflammatory response
TNFA signaling via NFKB
IL2 STAT5 signaling
IL6 JAK STAT3 signaling
normalized enrichment score
-log10(FDR)
Gene Set size
D
RNA-sequencing (K562)
HSP90α-KO vs. EV
Regulation of ERK1 & ERK2
MAPK cascade
Cell motility
Leukocyte development and function
NES
number of genes

Fig. S2 continued

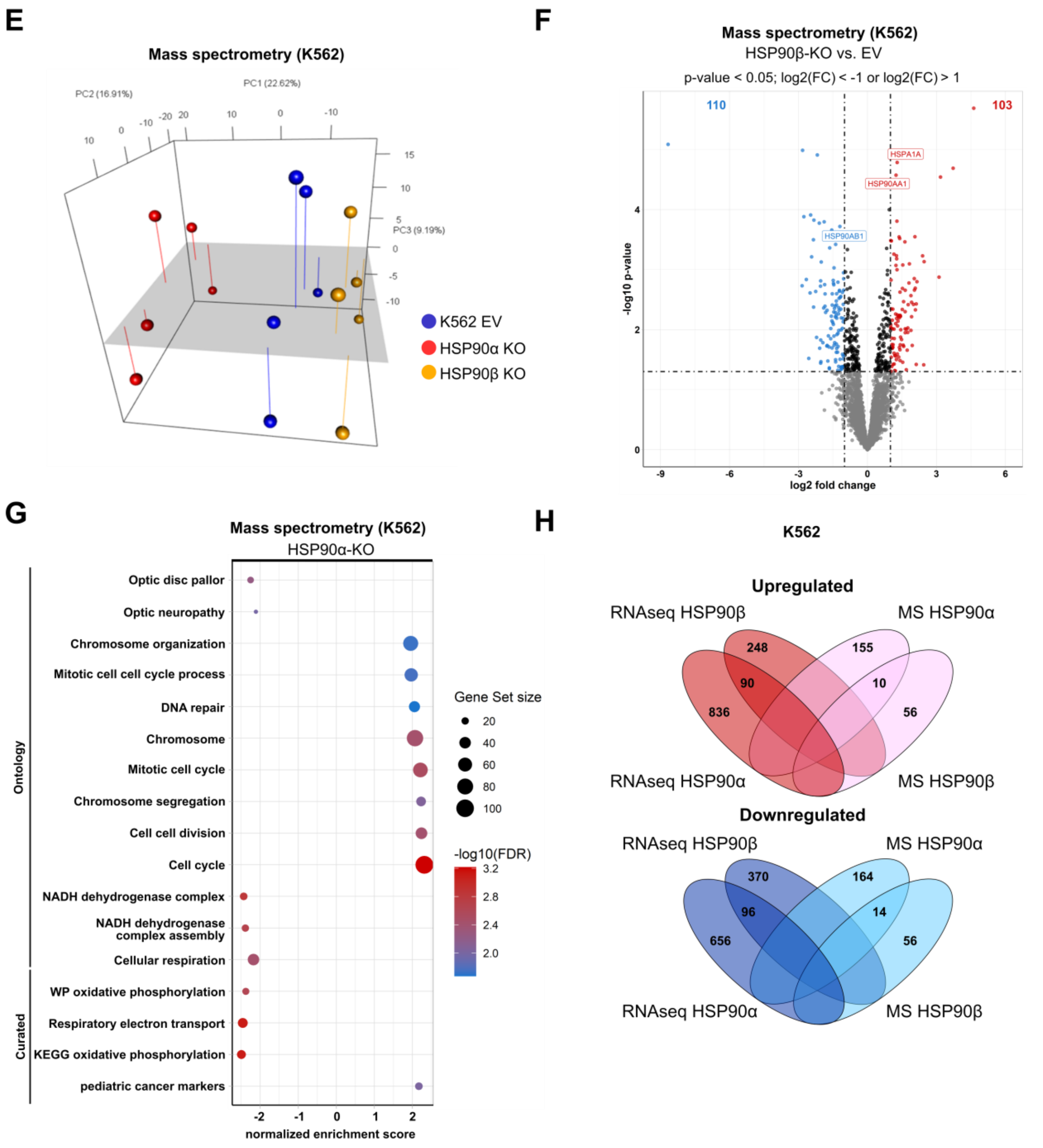

Supplemental Figure 2: Transcriptomic (RNA-sequencing) and quantitative mass spectrometry (MS) based proteomic analysis of HSP90α/β-KO K562 cells. (**A**) Principal component analysis (PCA) plot of RNA-seq data of HSP90α/β-KO and empty vector (EV) cells showing data conformity in the triplicates. (**B**) Volcano plot of the significantly (FDR < 0.05; log2(FC) < -1 or log2(FC) > 1) up- or down-regulated genes of HSP90β-KO cells in the mRNA expression profile (RNA-seq). Black dots represent genes that are not significantly regulated, while grey dots represent significantly regulated genes, but below log2(FC) threshold. Blue and red dots represent significantly downregulated and upregulated genes, respectively. (**C**) fGSEA showing significantly (FDR = 0.05) differentially regulated gene set signatures in HSP90α-KO cells compared to EV control cells from RNA-seq analysis. (**D**) Gene clusters

obtained using clusterProfiler on the RNA-seq data of HSP90α-KO cells. Normalized enrichment scores (NES). (**E**) PCA plot of HSP90α/β-KO and empty vector (EV) cells showing data conformity in five replicates of the MS measurement. (**F**) Volcano plot obtained from five independent replicates of HSP90β-KO compared to EV control K562 cells showing up- or down-regulated proteins based on MS-based proteomics data applying p-value < 0.05 and log2(FC) < -1 or log2(FC) > 1 as the specificity cutoff criteria. (**G**) fGSEA showing significantly (FDR = 0.05) differentially regulated gene set signatures in HSP90α-KO cells in comparison to EV control cells from MS measurement. (**H**) Venn diagrams showing overlapping genes between RNA sequencing and MS data of HSP90α-KO and HSP90β-KO cells, (upper) upregulated and (lower) downregulated genes.

Fig. S3

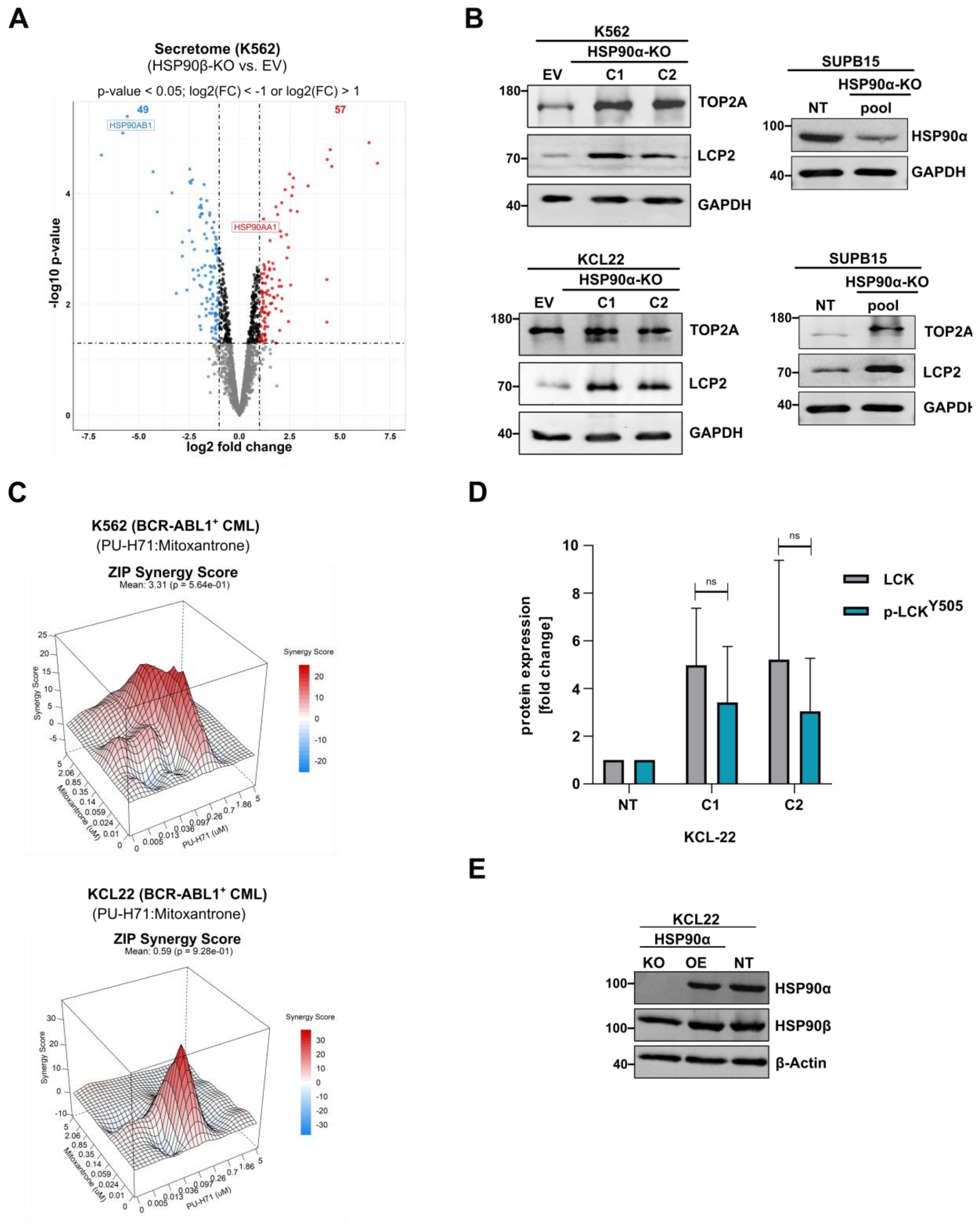

Supplemental Figure 3: MS based secretome analysis of HSP90β-KO cells and validation of overlapping hits found in different multi-omics approaches. (A) Volcano plot obtained from five independent replicates of HSP90β-KO compared to EV control K562 cells showing up- or down-regulated proteins based on MS-based proteomics data applying p-value < 0.05 and log2(FC) < -1 or log2(FC) > 1 as the specificity cutoff criteria. Black dots represent secreted proteins that are not significantly regulated,

while grey dots represent significantly regulated secreted proteins, but below log2(FC) threshold. Blue and red dots represent significantly downregulated and upregulated secreted proteins, respectively. The third replicate of the EV control in the secretome data was omitted from the statistical analysis due to its significant deviation from the other four replicates. (**B**) WB analysis of LCP2 and TOP2A expression in K562 and KCL22 cells (left panel). WB analysis of HSP90α-KO in BCR-ABL1+ BCP-ALL cell line SUPB15 (right top panel). As monoclonal section of HSP90α-KO SUPB15 cells was not possible in several attempts, therefore HSP90α-KO pool cells were used to determine the LCP2 and TOP2A expression (right bottom panel). Glyceraldehyde 3-phosphate dehydrogenase (GAPDH) served as a loading control. (**C**) Synergy maps of PU-H71 and Mitoxantrone (TOP2i) combination matrix for K562 (upper panel) and KCL22 (lower panel) cells, using Zero Interaction Potency (ZIP) method (*1*). Visualization was done using SynergyFinder package. (**D**) Bars show average protein quantification measurements of LCK and p-LCK^{Y505} levels in HSP90α-KO cells compared to non-targeting control (KCL22) cells. Error bars = SD of three independent replicates; significance was calculated by unpaired two-tailed student's t-test. (**E**) WB analysis showing rescue experiment. KCL22 HSP90α-KO cells were transiently transfected with HSP90α overexpression (OE) construct. NT stands for non-targeting control. β-actin served as a loading control.

Fig. S4

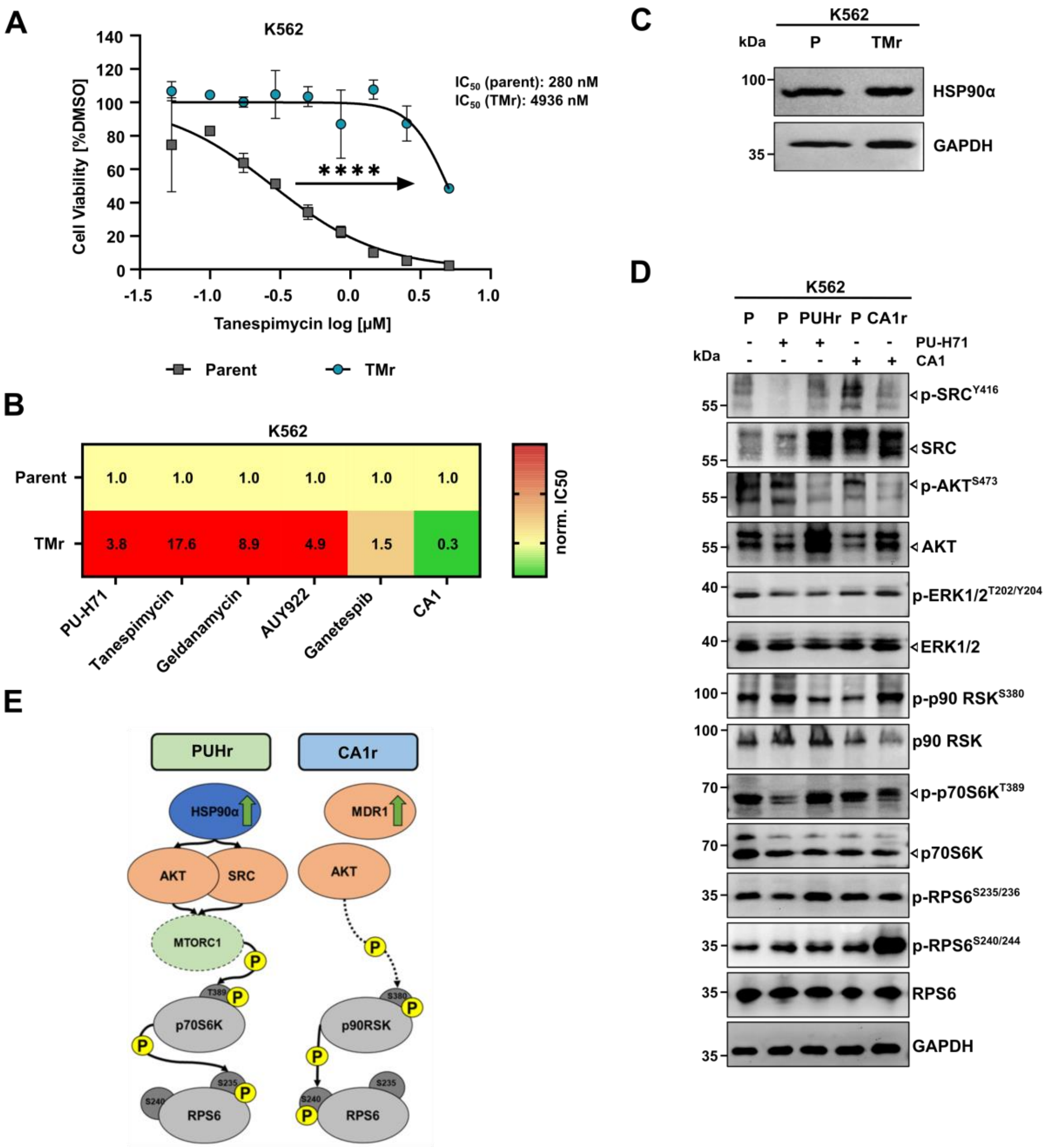

Supplemental Figure 4: Generation of HSP90i Tanespimycin (TM) resistant K562 cells. Analysis of kinase signaling in PU-H71 resistant (PUHr) and Coumermycin A1 resistant (CA1r) K562 cells. (**A**) Dose response curves showing significant (*** p≤0.001, paired two-tailed student's t-test) increase in IC_{50} values for TM-resistant (TMr) cells in comparison to the parental counterparts. (**B**) Cross resistance of TMr cells to other HSP90i with similar or different mode of action (MOA). The numbers in the heat map indicate the normalized fold-change of the IC_{50} values of the resistant cell lines to the parental counterpart. The red colour depicts an increase in IC_{50} value whereas green indicates a decrease in IC_{50} value in comparison to the parental control. (**C**) WB analysis revealed no change in the expression of HSP90α in TMr cells. GAPDH served as a loading control. (**D**) WB analysis revealed differences between PUHr, CA1r and respective parent (P) cells in kinase signaling-related proteins upon re-treatment with CA1 (2 μM), PU-H71 (500 nM) or vehicle (-) for 24h. GAPDH served as a loading

control. (**E**) Schematic depiction of the different routes and kinase signaling cascade implicated in conferring resistance against HSP90i with different (MoA).

Fig. S5

A

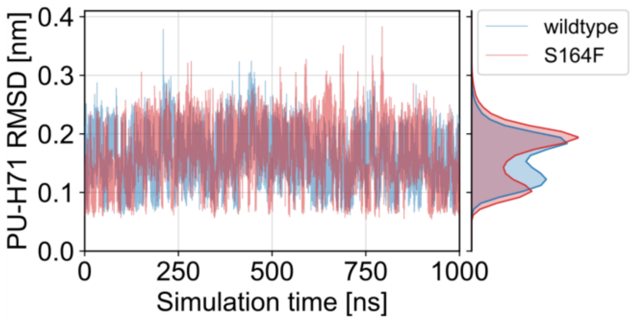

B

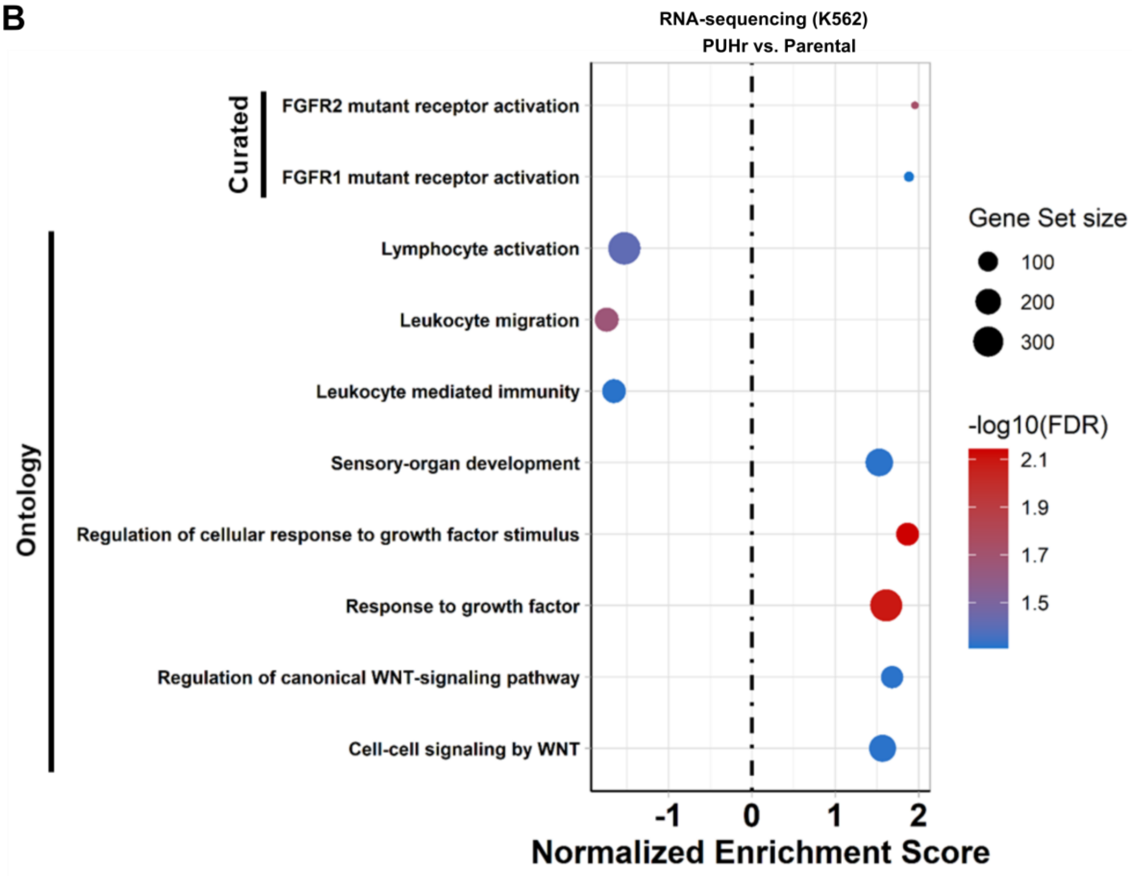

Supplemental Figure 5: Molecular dynamics (MD) simulations of PU-H71 bound to HSP90α and the gene sets enriched in PU-H71-resistant (PUHr) cells compared to parental (P) cells. (**A**) Shown is the RMSD of PU-H71 bound to wildtype-HSP90α (blue) and the S164F variant (red). In both systems PU-H71 remained stably bound over the course of the simulation. RMSD values are generally < 0.3 nm in both simulations and the overall distributions of values are very similar. (**B**) fGSEA showing significantly (FDR = 0.05) differentially regulated gene set signatures in PUHr cells in comparison to parental cells.

Fig.S6

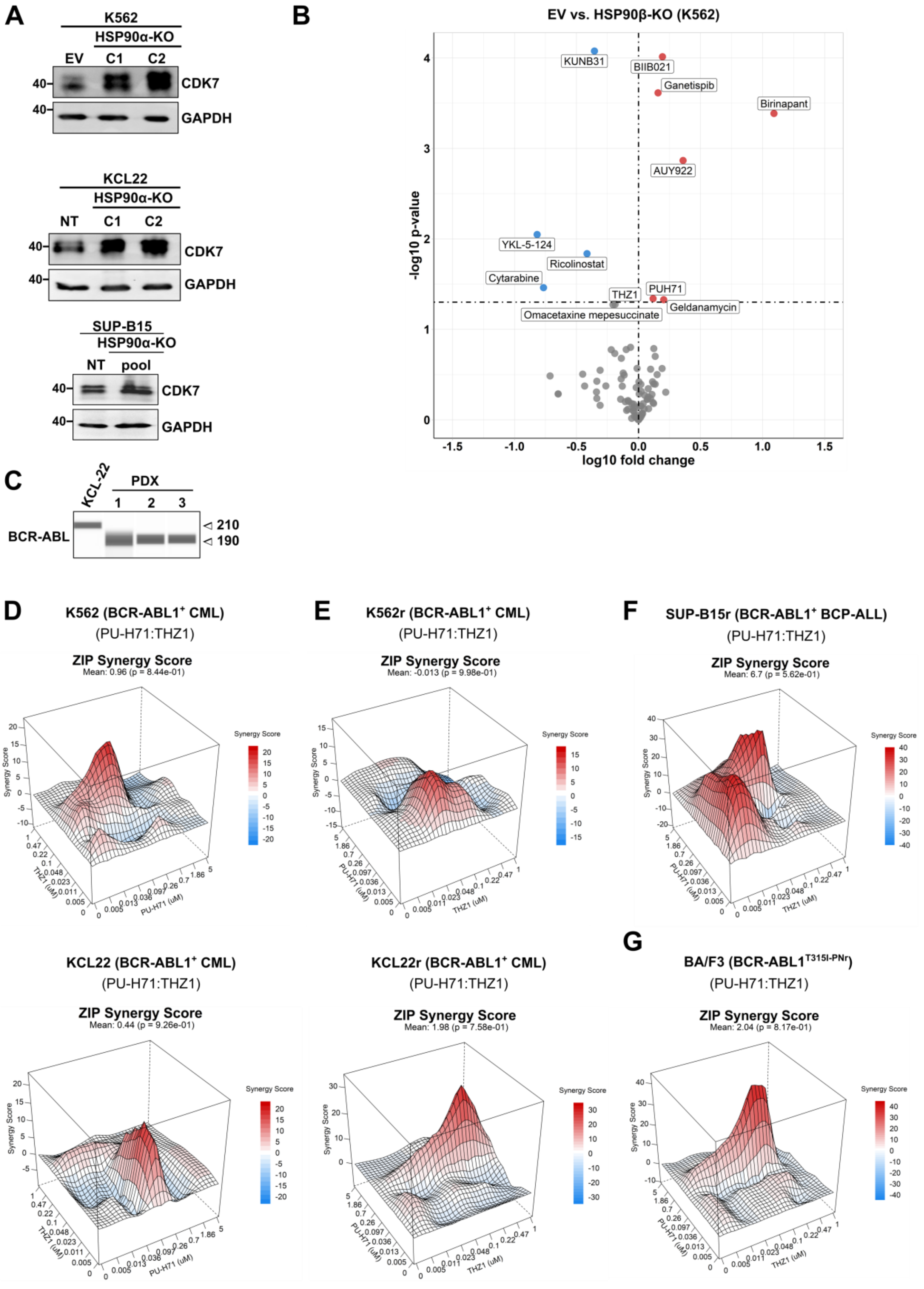
A
K562
HSP90α-KO
EV
C1
C2
40
CDK7
GAPDH
KCL22
HSP90α-KO
NT
C1
C2
CDK7
GAPDH
SUP-B15
HSP90α-KO
NT
pool
CDK7
GAPDH
B
EV vs. HSP90β-KO (K562)
KUNB31
BIIB021
Ganetespib
Birinapant
AUY922
YKL-5-124
Ricolinostat
Cytarabine
THZ1
PUH71
Geldanamycin
Omacetaxine mepesuccinate
-log10 p-value
log10 fold change
C
KCL-22
PDX
1
2
3
BCR-ABL
210
190
D
K562 (BCR-ABL1+ CML)
(PU-H71:THZ1)
ZIP Synergy Score
Mean: 0.96 (p = 8.44e-01)
E
K562r (BCR-ABL1+ CML)
(PU-H71:THZ1)
ZIP Synergy Score
Mean: -0.013 (p = 9.98e-01)
F
SUP-B15r (BCR-ABL1+ BCP-ALL)
(PU-H71:THZ1)
ZIP Synergy Score
Mean: 6.7 (p = 5.62e-01)
KCL22 (BCR-ABL1+ CML)
(PU-H71:THZ1)
ZIP Synergy Score
Mean: 0.44 (p = 9.26e-01)
KCL22r (BCR-ABL1+ CML)
(PU-H71:THZ1)
ZIP Synergy Score
Mean: 1.98 (p = 7.58e-01)
G
BA/F3 (BCR-ABL1T315I-PNr)
(PU-H71:THZ1)
ZIP Synergy Score
Mean: 2.04 (p = 8.17e-01)
Synergy Score
THZ1 (uM)
PU-H71 (uM)

Fig.S6 continued

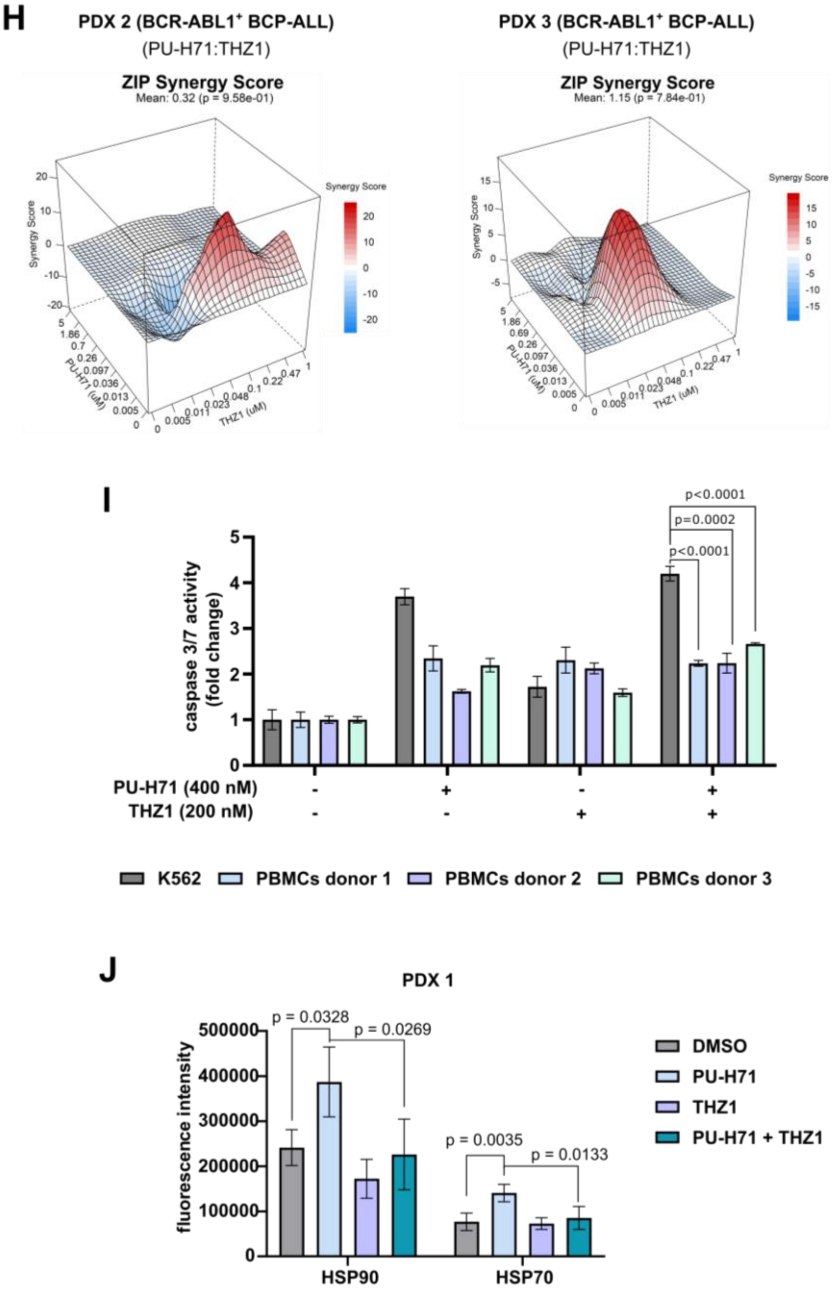

Supplemental Figure 6: *Ex vivo* high throughput drug screening of HSP90β-KO K562 cells. Combinatorial targeting of CDK7 and HSP90α acts synergistically against BCR-ABL1+ leukemia cells. (A) WB analysis of the expression level of CDK7 in K562, KCL22, and SUP-B15 HSP90α-KO clones or pool cells revealed an upregulation of CDK7. GAPDH served as a loading control. (**B**) Comparative cell viability was measured by luminescent-based ATP-Glo assay after screening HSP90β-KO K562 cells on an *ex vivo* HTDS platform, including 93 inhibitors. Average IC_{50} values from three independent replicates are depicted in the volcano plots compared to the empty vector (EV) control. Significance was calculated using unpaired t-test. (**C**) WB (JESS) analysis of the BCR-ABL1 expression in three BCR-ABL1+ BCP-ALL PDX samples. Synergy maps (generated using ZIP method (*1*) of PU-H71 and THZ1 (CDK7i) combination matrix using K562 and KCL22 cells (**D**) and multi tyrosine kinase inhibitor (TKI)-resistant counterparts of K562 and KCL22 cell lines, referred to as K562r and KCL22r (**E**). Synergy maps of the PU-H71 and THZ1 combination using multi tyrosine kinase inhibitor resistant

SUPB15 cells, referred to as SUPB15r (**F**) and murine pro B cell line model BA/F3, expressing multi TKI-resistant BCR-ABL1^{T315I} mutant made resistant to the third generation TKI Ponatinib, referred to as BA/F3 BCR-ABL1$^{T315I\text{-}PNr}$ (**G**). (**H**) Synergy maps of the PU-H71 and THZ1 combination using two relapsed (TKI-resitant) BCR-ABL1+ BCP-ALL patient derived xenograft (PDX) cells. The visualization of the synergy data was performed using SynergyFinder package. (**I**) Caspase 3/7 glo assay (which assesses apoptosis induction) was conducted following the treatment of peripheral blood-derived mononuclear cells (PBMCs) obtained from three healthy individuals. These cells were treated with PU-H71, THZ1, either alone or in combination, and their response was compared to that of leukemia K562 cells. Error bars = SD of three independent replicates; p-values were calculated by unpaired two-tailed student's t-test. (**J**) Quantification of the immunofluorescence imaging data (shown in main Figure 6E) to assess the levels of HSP90 and HSP70 proteins, after treatment of BCR-ABL1+ BCP-ALL PDX cells with PU-H71, THZ1, either alone or in combination. Error bars = SD (n=4); p-values were calculated by unpaired two-tailed student's t-test.

Supplemental Table 1: Whole Exome Sequencing (WES) of Parental cells vs. PUHr or CAr (K562) cells

Related to Figure 5

Sample	chr	MAPINFO	REF	ALT	SYMBOL	HGVSc	CADD_PHRED	SIFT	PolyPhen	Allele frequency in resistant cells
CAr	14	95203587	C	G	CLMN	ENST00000298912.9:c.1762G>C	12,45	deleterious	benign	0.312775
PUHr	14	95210838	C	T	CLMN	ENST00000298912.9:c.650G>A	23,6	tolerated	probably damaging	0.465753
PUHr	14	102085796	G	A	HSP90AA1	ENST00000216281.13:c.491C>T	32	deleterious	probably damaging	0.553459
CAr	14	102086292	C	A	HSP90AA1	ENST00000216281.13:c.87G>T	23,5	deleterious	probably damaging	0.276074

Supplemental Table 2: Compounds List in the Drug screening Library

Related to Figure 6

No.	Name	Target
1	Coumermycin A1	HSP90
2	EC144	HSP90
3	Ganetespib	HSP90
4	Geldanamycin	HSP90
5	Tanespimycin	HSP90
6	PU-H71	HSP90
7	KUNB31	HSP90
8	BIIB021	HSP90
9	AUY922 (LUMINESPIB)	HSP90
10	Panobinostat	HDAC
11	Ricolinostat	HDAC6
12	Romidepsin	HDAC

13	Belinostat	HDAC
14	Entinostat	HDAC
15	Givinostat (ITF2357)	HDAC
16	CI-994 (Tacedinaline)	HDAC
17	Dasatinib (Hydrochlorid)	BCR-ABL; Src;
18	Imatinib (Mesylate)	BCR-ABL inhibitor
19	Ponatinib	BCR-ABL; FGFR; FLT3; VEGFR;
20	Bosutinib	BCR-ABL; Src;
21	Dinaciclib	CDK
22	LY2835219 (Abemaciclib)	CDK
23	Palbociclib	CDK
24	YKL-5-124	CDK7
25	SY-1365-THZ1	CDK7
26	Volasertib	Polo-like Kinase (PLK)
27	BI2536	PLK
28	6-Mercaptopurine (Monohydrate)	Antimetabolites
29	6-Thioguanine	Antimetabolites
30	Clofarabine	Antimetabolites
31	Cyclocytidine HCL	Antimetabolites
32	Methotrexate	Antimetabolites
33	5-Azacytidine	Antimetabolites
34	Cytarabine (Hydrochlorid)	Antimetabolites
35	Vinblastine (sulfate)	Antimitotics
36	Vincristine (sulfate)	Antimitotics
37	Alisertib	Aurora Kinase A
38	Barasertib	Aurora kinase B
39	Aurora A Inhibitor I	Aurora Kinase A inhibitor
40	Birabresib	BRD2/3/4
41	Carfilzomib	Proteasome inhibitor
42	Bortezomib	Proteasome inhibitor
43	MLN-9708 (Citrate)	Proteasome inhibitor
44	Cobimetinib	MEK
45	MEK162 (Binimetinib)	MEK
46	Lonafarnib	RAS
47	Regorafenib (Monohydrate)	RAF
48	Tipifarnib	Farnesyl Transferase; RAS
49	Sorafenib (Tosylate)	RAF
50	Trametinib	MEK
51	AT9283	JAK
52	CYT387 (Momelotinib)	JAK
53	Fedratinib (TG101348)	JAK; FLT3
54	BSI-201 (Iniparib)	PARP

55	Olaparib	PARP
56	GSK343	EZH2i/HMTasei
57	Ibrutinib (Ibruvica)	BTK
58	Tirabrutinib	BTK
59	Amsacrine (Hydochlorid)	Topoisomerase inhibitors
60	Daunorubicin (Hydrochloride)	Topoisomerase inhibitors
61	Mitoxantrone (dihydrochloride)	Topoisomerase inhibitors
62	Dovitinib	FLT3; PDGFR; VEGFR; c-Kit;
63	Gilteritinib	FLT3/AXL
64	Lestaurtinib	FLT3
65	Midostaurin	PKC, vegfr2, pdgfr, FLT3
66	Pacritinib	FLT3; JAK;
67	Pexidartinib	FLT3, KIT, CSF1R
68	Quizartinib	FLT3;
69	Pacritinib	FLT3; JAK;
70	Pexidartinib	FLT3, KIT, CSF1R
71	Quizartinib	FLT3;
72	Staurosporin	Multiple non-selective inhibitor of protein kinases
73	Omaveloxolone	NF-kB
74	QNZ (EVP4593)	NF-kB
75	Birinapant	XIAP and cIAP1
76	AZD6738	ATM/ATR
77	Omacetaxine Mepesuccinate (Homoharringtonine)	Ribosome Inhibtor
78	Selinexor	CRM1 inhibitor
79	Nintedanib (BIBF1120)	LCK inhibitor
80	Ro 08-2750	NGF inhibitor
81	Bexarotene	Retinoid Inhibitor
82	ABT-199 (Venetoclax)	Bcl-2 Family;
83	Obatoclax (Mesylate)	Bcl-2 Family;
84	Temsirolimus	mTOR;
85	PF-04691502	mTOR; PI3K;
86	ARQ-092 (Miransertib)	AKT
87	BAY 80-6946 (Copanlisib)	PI3K
88	Dactolisib (BEZ235)	PI3K, mTOR
89	Idelalisib	PI3K;
90	Everolimus	mTOR;
91	5-nonyloxy-tryptamine	5-HT1B receptor agonist
92	Tegaserod	5-HT4/serotonin agonist
93	Axitinib	VEGFR

2. Supplemental Material and Methods:

Conditional knockdown (KD) and CRISPR-Cas9 mediated knockout (KO): Following lentivirus transduction and antibiotic (puromycin, 0.5 µg/ml) selection (*2*), GFP or RFP expression was induced by doxycycline, followed by sorting positive cells via FACS (MoFlo XDP, Beckman-Coulter). Pellets were recovered after incubating the cells with 2 µg/ml doxycycline for 72 h.

Target	Mature antisense sequence (5' – 3')
**sh1 (HSP90α)**	TTAATATGCAGCTCTTTCC
**sh2 (HSP90α)**	TAATATGCAGCTCTTTCCC
**sh1 (HSP90β)**	GAATGCTTCTTCACTACTT

Generation of HSP90α/β-KO K562 cells: For generation of KO cells, the following guide RNA (gRNA) sequences were utilized:

Construct	Sequence (5' – 3')
**gRNA_HSP90AA1**	GACCCAAGACCAACCGATGG
**gRNA_HSP90AB1**	CATTAGAGATCAACTCCCGA

gRNAs were cloned into the lentiviral expression plasmid with GFP or mCherry (Addgene ID #108098 and #108099). Virus production was done in Lenti-X HEK293T cells using Lenti-X Packaging Single Shots (Takara) according to manufacturer's instructions. After 72 h in total, the lentiviral supernatant was harvested, filtered through a 0.45 µm filter and added to constitutively expressing Cas9 K562 cells (0.4 x 10^6 cells; LentiV-Cas9-puro; Addgene ID #108100) that were previously generated by viral transduction and puromycin selection (puromycin, 0.5 µg/ml). After transduction, positive cells were selected by the fluorescent marker, GFP or mCherry.

Generation of KCL-22 and SUP-B15 KO cells: The transfection was carried out using the Amaxa Nucleofection system (SF Cell Line Kit, #V4XC-2032). For 2 x 10^5 cells, 100 pmol of Cas9-GFP protein (IDT, Alt-R S.p. HiFi Cas9-GFP V3, #10008100) was mixed with 120 pmol of gRNA (crRNA:tracrRNA 1:1) and assembled for 20 min at RT. Afterwards, the labelled ssODN was added and the mixture was combined with the cell suspension (resuspended cells in Nucleofector solution SF) and the electroporation

enhancer (IDT). The complete volume was transferred to the Nucleocuvette module, placed in the 4D-Nucleofector system (Lonza) and electroporated with the program CA-137 (KCL-22) or CV-104 (SUP-B15). After 72 h, the cells were sorted for GFP and monoclonal selected via semi-solid cloning.

Quantitative real-time PCR: RNA extraction was performed with the Maxwell RSC viral total nucleic acid purification kit (Promega, Madison, WI, USA) with the Maxwell RSC 48, following manufacturer's instructions. 2 µg of total RNA was used for cDNA synthesis (QuantiTect Reverse Transcription kit, Qiagen, Hilden, Germany). Quantitative real-time PCR was carried out using BioRad cycler (BioRad). Reactions were carried out in triplicates in three independent experiments. The mean Ct values of the housekeeping gene B2M and GAPDH was used to normalize the variability in expression levels. Primer sequences:

Target	Forward primer sequence (5'-3')	Reverse primer sequence (5'-3')
**HSP90AA1**	TCTGCCTCTGGTGATGAGATGG	CGTTCCACAAAGGCTGAGTTAGC
**HSP90AB1**	CTCTGTCAGAGTATGTTTCTCGC	GTTTCCGCACTCGCTCCACAAA
**HSPA1A (HSP70)**	ACCTTCGACGTGTCCATCCTGA	TCCTCCACGAAGTGGTTCACCA

Mass Spectrometery (MS) based proteome anaylsis:

a) *Sample preparation*

   i) *Proteomes*

   K562 cells (EV, HSP90α-KO (C1), or HSP90β-KO (C1); five biological replicates) were washed three times with PBS and shock frozen in liquid nitrogen. Proteins were extracted from frozen cell pellets as described elsewhere(*3*). Briefly, cells were lysed and homogenized in urea buffer using a TissueLyser (Qiagen) and, after centrifugation (15 min, 16000 rcf, 4°C), supernatants were collected. After determination of protein concentration (Pierce 660 nm Protein Assay, Thermo Fischer Scientific), samples were adjusted to 0.5 mg/ml total protein concentration with SDS buffer (final 7.5% glycerol, 3% SDS, 37.5 mM Tris/HCl pH 7.0) and 10 µl were reduced (final 20 mM dithiothreitol, 20 min, 56°C), alkylated (final 80 mM iodoacetamide, 15 min, r.t., protected from light) and finally underwent tryptic digestion (200

ng trypsin in 50 mM triethylammonium bicarbonate) after applying a slightly modified sp3 protocol(*4*) using 50 µg 1:1 mix Sera-Mag SpeedBeads. Peptides were reconstituted in 0.1% trifluoracetic acid and subjected to LC-MS analysis.

ii) *Secretomes*

K562 cells (EV, HSP90α-KO (C1), or HSP90β-KO (C1); five biological replicates) were washed three times with PBS and three times with FCS-free medium and incubated for 24 h in FCS-free medium at a density of 0.8 million cells / mL. The conditioned medium was collected by centrifugation (5 min, 800 x g, 4°C) and filtering through a 0.2 µm membrane (Acrodisc 32 mm Syringe Filter with 0.2 µm Supor Membrane; Pall, #4652). Aliquots were shock frozen in liquid nitrogen and stored at -80°C. An aliquot (400 µl) per cell type and replicate was thawed on ice in the presence of protease inhibitor cocktail (added 50 µl of a solution of 1 cOmplete ULTRA tablet, mini, EDTA-free in 2 mL water; Roche, #05892791001), supplemented with SDS buffer (added 50 µl of 30% glycerin, 12% SDS, 150 mM Tris base), reduced (added 40.5 µL of 100 mM dithiothreitol; 20 min at 56 °C under shaking), alkylated (added 54 µL of 300 mM iodacetamide; 15 min at r.t. protected from light), and quenched (added 40.5 µL of 100 mM dithiothreitol; 20 min at r.t.). Applying a slightly modified sp3 protocol (*4*), proteins were precipitated (added 10 µL of 20 mg/mL 1:1 bead-mix of pre-washed Sera-Mag SpeedBeads GE #45152105050250 and #65152105050250 in water; added 645 µL ethanol abs. p.a.; 15 min at 24 °C under shaking), washed (3x 80% ethanol, 1x acetonitrile) and digested (100 ng trypsin in 20 µL 50 mM triethylammonium bicarbonate). Peptides were reconstituted in 0.1% trifluoracetic acid and subjected to LC-MS analysis.

b) *LC-MS analysis:*

For the LC-MS analysis, a Q Exactive Plus Hybrid Quadrupole-Orbitrap (for proteomes) or a Orbitrap Fusion Lumos Tribrid (for secretomes) mass spectrometer (Thermo Fisher Scientific), operated in positive mode and coupled with a nano electrospray ionization source connected with an Ultimate 3000 Rapid Separation liquid chromatography system (Dionex / Thermo Fisher Scientific, Idstein, Germany) equipped with an Acclaim PepMap 100 C18 column (75 µm inner diameter, 25 cm length, 2 mm particle size from Thermo Fisher Scientific) was applied using a 120 min

LC gradient. Capillary temperature was set to 250°C or 275°C and source voltage to 1.4 kV or 1.5 kV for the Q Exactive or Lumos mass spectrometer, respectively. MS survey scans had a mass range from 350 (Q Exactive) or 200 (Lumos) to 2000 m/z at a resolution of 140,000 (Q Exactive) or 120,000 (Lumos). The automatic gain control (AGC) was set to 3,000,000 (Q Exactive) or the normalized AGC target was set to 62.5% (Lumos) and the maximum fill time was 80 ms (Q Exactive) or 60 ms (Lumos). The ten most intensive peptide ions per survey scan were isolated and fragmented by high-energy collision dissociation (HCD) (Q Exactive) or a cycle time of 2 s was employed (Lumos).

c) *Data analysis*:

MaxQuant (version 2.0.3.0 for proteomes or 2.2.0.0 for secretomes, Max Planck Institute for Biochemistry, Planegg, Germany) was used for peptide / protein identification and quantification employing a human sequence database (UniProtKB, downloaded on 01/27/2021, 75777 entries for proteomes or on 18/01/2022, 79038 entries for secretomes). Methionine oxidation and N-terminal acetylation as well as a carbamidomethylation at cysteine residues were considered as variable and fixed modifications, respectively. A false discovery rate of 1% on protein and peptide level was set as identification threshold. A total of 4676 protein groups were identified for proteomes and 2051 for secretomes after removing potential contaminants, reverse hits, proteins only identified by modified peptides, and proteins without valid intensity or MS/MS count values. Protein intensities for HSP90 isoforms were examined and, if necessary, manually re-assigned from the sum of corresponding peptide intensities.

Statistical analysis was performed based on experiment-pairwise median $\log_2$(fold change) normalized and $\log_2$ transformed MaxQuant protein group intensities and LFQ intensities using the “R” (v4.0.4 for proteomes and v4.2.1 for secretomes) programming language. Principal component analysis (PCA) was performed using the prcomp() function with centering and without scaling on protein groups with a complete set of valid values over all samples. Differential analyses (HSP90α-KO vs. EV or HSP90β-KO vs. EV) were performed using the siggenes package for evaluating p-values. For this approach, a minimum of four valid values had to be present in at least one group (EV or KO) and missing values were filled in with random values from sample wise downshifted normal distributions of the $\log_2$ transformed data (0.3 s.d. width, 1.8 s.d. downshift). Replicate three of the EV samples of the secretomes was

excluded from statistical analysis because it differed substantially from the other four replicates.

The mass spectrometry based proteomics or secretomics raw and expression data have been deposited to the ProteomeXchange Consortium via the PRIDE (*5*) partner repository with the dataset identifier PXD041871 (Username: reviewer_pxd041871@ebi.ac.uk, Password: uXMhlijm).

Whole-exome sequencing (WES): After demultiplexing and adapter trimming using bcl2fastq and Trimmommatic (*6*), we aligned the reads to the hg38 reference genome using BWA mem algorithm (*7*). Variant calling was performed applying Varscan v.2.3.8 (*8*), Muse v1.0 (*9*), Strelka v.2.9.1.0 (*10*) , Mutect2 (*11*) and LoFreq v.2 (*12*) in the somatic mode using the parental K562 cell line as a control, enabling identification of variants acquired during treatment. The identified variants were combined and filtered using SomaticCombiner v1.02 (*13*) and annotated using Ensembl Variant Effect Predictor v104 (*14*).

Sanger sequencing was performed to validate the herein reported variants using the following primers:

Name	Sequence (5-3')
HSP90AA1_S164F_F	CCCCAATCACCTACAGACAGA
HSP90AA1_S164F_R	CCAGACCCAAGACCAACCGATG
HSP90AA1_L29F_F	AGCATACAGCACCCCCAAGA
HSP90AA1_L29R_R	GGAAACCCAGACCCAAGACC
CLMN_E588Q_F	TGGGCTCCCTTCAAAGCAAA
CLMN_E588Q_R	AAGGAATTCCGCCTGGATGG
CLMN_S217N_F	GGACCAACTGTATAGACCAT
CLMN_S217N_R	AGTAGCTGTCATTAGTGAGT

Molecular dynamics (MD) simulation: The atomistic MD-simulations of wildtype-HSP90α and the S164F variant were performed in GROMACS v.2020.1 (*15-17*) using the AMBER99SB-ILDN forcefield (*18*) for the protein and GAFF (*19*) for PU-H71. The partial charges of the ligand were derived via the RESP method (*20*) from *ab inito* calculations to Gaussian 16 A.03 (*21*) at the HF level of theory. The 6-31G(d) basis set was used for all atoms except iodine for which aug-cc-pVDZ-PP was used.

To model sigma-hole interactions of the iodine, an additional pseudo atom was introduced and charge fitted as described by Rendine *et al.* (*22*). The starting structure was solvated in TIP3P water model (*23*) in a rhombic dodecahedral box; the protein was placed at least 1.4 nm from the box edges. Na^+ ions were added to neutralize the system. An initial relaxation of the system was performed over 50,000 steps of steepest descent algorithm, followed by 100 ps NVT simulationunder at 300 K using the Berendsen thermostat(*24*), and 100 ps NPT simulation at 300 K and 1 atm using the Parrinello-Rahman barostat (*25*).
The final production was performed for a total of 1 μs for the wildtype and S164F HSP-90α. Covalent bonds were constrained using the LINCS algorithm (*26*) and Particle Mesh Ewald (PME) algorithm (*27*) was used to calculate long range interaction. Leap frog integrator was used to calculate the equation of motion with a timestep of 2 fs (*28*). Visual Molecular Dynamics (VMD) program (*29*) was used to perform the visual analysis of the final trajectories. The RMSD and RMSF were calculated using the GROMACS modules *gmx rmsf* and *gmx rms*.

Western Blotting: Cells were harvested by centrifugation at 400 x g for 5 min at 4°C and washed three times with ice-cold PBS and then snap-frozen in liquid nitrogen. 50 µL RIPA lysis buffer (Thermo Fisher Scientific, #89900) supplemented with cOmplete^{tm} (Roche, #11697498001) and PhosSTOP (Sigma-Aldrich, #4906845001) was used per 1 million cells. Cells were lysed on ice for 1h with periodically vortexing, centrifuged two times (10000 x g for 20 min at 4 °C) and protein quantification was performed by BCA-Assay (Thermo Fisher Scientific, #23227). 10 - 20 µg lysate were separated by SDS-PAGE at 50 V for 30 mins during stacking phase with subsequent 100 V for 2h during seperation phase and blotted onto 0.45 µm nitrocellulose membrane (Cytiva, #10600002) at 100 V for 1h or overnight (30 V, 16 h). Membranes were washed twice with TBS and analyzed for their protein content by ponceau staining (Sigma-Aldrich #P7170) staining. After three washes with TBS-T, membranes were blocked in 5% BSA (Sigma-Aldrich, #A3294) TBS-T solution, washed three times with TBS-T and incubated with primary antibody solution in 5% BSA solution overnight at 4°C. Membranes were washed three times with TBS-T, incubated for 1h with secondary HRP-conjugate (Cell Signaling Technologies, #7074 and #7076) at 1:2000 in TBS-T solution, washed again three times in TBS-T and lastly one time in TBS. For

visualization ECL-Solution (Cytiva, #GERPN2106) was used as per manufacturer's instruction and image was captured using JESS.

Simple western immunoassay (JESS): Reagents of the EZ standard pack were prepared as per manufacturer's instruction. After lysate denaturation for 5 min at 95°C in a PCR cycler (GeneAMP PCR System2700, Applied Biosystems) samples were loaded onto the assay plate at a final concentration of 0.40 µg/µL in 0.1x sample buffer supplemented with fluorescence master mix. Following standard assay plate loading and centrifugation for 5 min at 1000 x g at RT, a 12-230 kDA separation module with 25 cartridges (BioTechne, #SM-W004) was utilized. Lysates were separated 25 min at 375 volt, blocked for 5 min with antibody diluent 2, 90 min incubated with primary antibody and 30 min incubated with secondary antibody, subsequently. Signals were detected using an anti-rabbit detection module of JESS (BioTechne, #DM-001), multiplexed with an anti-mouse secondary NIR antibody (BioTechne, #043-821).

List of antibodies used in conventional or in JESS immunoblots:

Cell Signaling Technology (CST) Danvers, MA, USA
Santa Cruz (SC) Biotechnology, Dallas, TX, USA

Target	Species	Dilution (conventional)	Dilution (JESS)	Cat. No.
β-actin	Mouse	1:2000	1:50	MAB8929 (Biotechne)
GAPDH	Mouse	1:2000	-	CST#97166
GAPDH	Rabbit	1:2000	-	CST#5174
Nucleolin	Rabbit	-	1:50	CST#14574
HSP90α	Rabbit	1:2000	1:100	CST#8165
HSP90β	Rabbit	1:1000	1:100	CST#5087
HSP90	Rabbit	1:2000	1:100	CST#4877
HSP70	Rabbit	1:1000	-	CST#4872
HSP27	Mouse	1:1000	-	CST#2402
HSP40	Rabbit	1:2000	-	CST#4871
HSF-1	Rabbit	1:1000	-	CST#4356
GRP94	Rabbit	1:1000	-	CST#2104
TRAP1	Mouse	1:1000	-	SC-13557
CDC37	Mouse	1:1000	-	SC-13129
AHA1	Mouse	1:1000	-	SC-166065

FKBP5	Rabbit	1:1000	-	CST#12210
p-AKT (S473)	Rabbit	1:750	1:30	CST#4060
AKT (pan)	Mouse	1:1000	1:50	CST#2920
p-STAT5 (Y694)	Rabbit	1:1000	1:50	CST#9351
STAT5	Rabbit	1:1000	1:50	CST#9363
Survivin	Rabbit	1:1000	-	CST#2808
CDK4	Rabbit	1:1000	-	CST#12790
CDK6	Rabbit	1:1000	-	CST#13331
CDK7	Mouse	1:1000	1:30	CST#2916
BCR-ABL	Rabbit	1:1000	1:50	CST#2862
p-BCR-ABL (Y412)	Rabbit	-	1:10	CST#2865
CRKL	Mouse	1:1000	1:30	CST#3182
p-CRKL (Y207)	Rabbit	-	1:30	CST#3181
SLP-76 (LCP2)	Rabbit	1:1000	-	CST#4958
TOP2A	Rabbit	1:1000	-	CST#12286
RPB1	Mouse	1:1000	-	CST#2629
p-RPB1 (S5)	Rabbit	1:1000	-	CST#13523
PARP	Rabbit	1:1000	-	CST#9532
LCK	Rabbit	1:1000	-	CST#2752
p-LCK (Y505)	Rabbit	1:1000	-	CST#2751
CD45 (intracellular domain)	Rabbit	1:1000	-	CST#13917
LYN	Rabbit	1:1000	-	CST#2796
p-LYN (Y507)	Rabbit	1:1000	-	CST#2731
ADLH1A1	Rabbit	1:1000	-	CST#54135
SRC	Rabbit	1:1000	-	CST#2109
p-SRC	Rabbit	1:1000	-	CST#2101
p-p90RSK(S380)	Rabbit	1:1000	-	CST#9335
p90 RSK	Rabbit	1:1000	-	CST#9355
MDR1/ABCB1	Rabbit	1:1000	-	CST#13342
RPS6	Rabbit	1:1000	-	CST#2217
p-RPS6 (S235/236)	Rabbit	1:2000	-	CST#4858
p-RPS6 (S240/244)	Rabbit	1:1000	-	CST#5364
p-p70 S6 Kinase	Rabbit	1:1000	-	CST#9234
p70 S6 Kinase	Rabbit	1:1000	-	CST#2708
p44/42 MAPK (ERK1/2)	Rabbit	1:1000	-	CST#9102
p-p44/42 MAPK (ERK1/2) (T202/Y204)	Rabbit	1:2000	-	CST#4370

3. Supplemental References:

1. A. Ianevski, L. He, T. Aittokallio, J. Tang, SynergyFinder: a web application for analyzing drug combination dose-response matrix data. *Bioinformatics* **33**, 2413-2415 (2017).
2. S. Bhatia *et al.*, Control of AC133/CD133 and impact on human hematopoietic progenitor cells through nucleolin. *Leukemia* **29**, 2208-2220 (2015).
3. G. Poschmann *et al.*, High-Fat Diet Induced Isoform Changes of the Parkinson's Disease Protein DJ-1. *Journal of Proteome Research* **13**, 2339-2351 (2014).
4. C. S. Hughes *et al.*, Single-pot, solid-phase-enhanced sample preparation for proteomics experiments. *Nat Protoc* **14**, 68-85 (2019).
5. Y. Perez-Riverol *et al.*, The PRIDE database resources in 2022: a hub for mass spectrometry-based proteomics evidences. *Nucleic Acids Res* **50**, D543-D552 (2022).
6. A. M. Bolger, M. Lohse, B. Usadel, Trimmomatic: a flexible trimmer for Illumina sequence data. *Bioinformatics* **30**, 2114-2120 (2014).
7. H. Li, Aligning sequence reads, clone sequences and assembly contigs with BWA-MEM. *arXiv: Genomics*, (2013).
8. D. C. Koboldt, D. E. Larson, R. K. Wilson, Using VarScan 2 for Germline Variant Calling and Somatic Mutation Detection. *Curr Protoc Bioinformatics* **44**, 15 14 11-17 (2013).
9. Y. Fan *et al.*, MuSE: accounting for tumor heterogeneity using a sample-specific error model improves sensitivity and specificity in mutation calling from sequencing data. *Genome Biology* **17**, 178 (2016).
10. C. T. Saunders *et al.*, Strelka: accurate somatic small-variant calling from sequenced tumor-normal sample pairs. *Bioinformatics* **28**, 1811-1817 (2012).
11. D. Benjamin *et al.*, Calling Somatic SNVs and Indels with Mutect2. *bioRxiv*, 861054 (2019).
12. A. Wilm *et al.*, LoFreq: a sequence-quality aware, ultra-sensitive variant caller for uncovering cell-population heterogeneity from high-throughput sequencing datasets. *Nucleic Acids Res* **40**, 11189-11201 (2012).
13. M. Wang *et al.*, SomaticCombiner: improving the performance of somatic variant calling based on evaluation tests and a consensus approach. *Sci Rep* **10**, 12898 (2020).
14. K. L. Howe *et al.*, Ensembl 2021. *Nucleic Acids Res* **49**, D884-D891 (2021).
15. M. J. Abraham *et al.*, GROMACS: High performance molecular simulations through multi-level parallelism from laptops to supercomputers. *SoftwareX* **1**, 19-25 (2015).
16. H. Bekker *et al.*, in *4th International Conference on Computational Physics (PC 92)*. (World Scientific Publishing, 1993), pp. 252-256.
17. S. Pronk *et al.*, GROMACS 4.5: a high-throughput and highly parallel open source molecular simulation toolkit. *Bioinformatics* **29**, 845-854 (2013).
18. K. Lindorff-Larsen *et al.*, Improved side-chain torsion potentials for the Amber ff99SB protein force field. *Proteins: Structure, Function, and Bioinformatics* **78**, 1950-1958 (2010).
19. J. Wang, R. M. Wolf, J. W. Caldwell, P. A. Kollman, D. A. Case, Development and testing of a general amber force field. *Journal of computational chemistry* **25**, 1157-1174 (2004).
20. C. I. Bayly, P. Cieplak, W. Cornell, P. A. Kollman, A well-behaved electrostatic potential based method using charge restraints for deriving atomic charges: the RESP model. *The Journal of Physical Chemistry* **97**, 10269-10280 (1993).
21. M. J. Frisch *et al.* (Wallingford, CT, 2016).
22. S. Rendine, S. Pieraccini, A. Forni, M. Sironi, Halogen bonding in ligand-receptor systems in the framework of classical force fields. *Phys Chem Chem Phys* **13**, 19508-19516 (2011).
23. W. L. Jorgensen, J. Chandrasekhar, J. D. Madura, R. W. Impey, M. L. Klein, Comparison of simple potential functions for simulating liquid water. *The Journal of Chemical Physics* **79**, 926-935 (1983).
24. H. J. C. Berendsen, J. P. M. Postma, W. F. van Gunsteren, A. DiNola, J. R. Haak, Molecular dynamics with coupling to an external bath. *The Journal of Chemical Physics* **81**, 3684-3690 (1984).

25. M. Parrinello, A. Rahman, Polymorphic transitions in single crystals: A new molecular dynamics method. *Journal of Applied physics* **52**, 7182-7190 (1981).
26. B. Hess, H. Bekker, H. J. Berendsen, J. G. Fraaije, LINCS: a linear constraint solver for molecular simulations. *Journal of computational chemistry* **18**, 1463-1472 (1997).
27. U. Essmann *et al.*, A smooth particle mesh Ewald method. *The Journal of chemical physics* **103**, 8577-8593 (1995).
28. W. F. Van Gunsteren, H. J. Berendsen, A leap-frog algorithm for stochastic dynamics. *Molecular Simulation* **1**, 173-185 (1988).
29. W. Humphrey, A. Dalke, K. Schulten, VMD: visual molecular dynamics. *Journal of molecular graphics* **14**, 33-38 (1996).
