# Supplementary material for: Co-targeting HSP90 alpha and CDK7 overcomes resistance against HSP90 inhibitors in BCR-ABL1+ leukemia cells
Source: Cell Death Dis. 2023 Dec 6;14(12):799. doi: 10.1038/s41419-023-06337-3 (PMC10700369; doi:10.1038/s41419-023-06337-3)

Original Western Blots

(Red box indicate the immunoblots which are shown in main or supplementary file)

Main Figure 1A - left

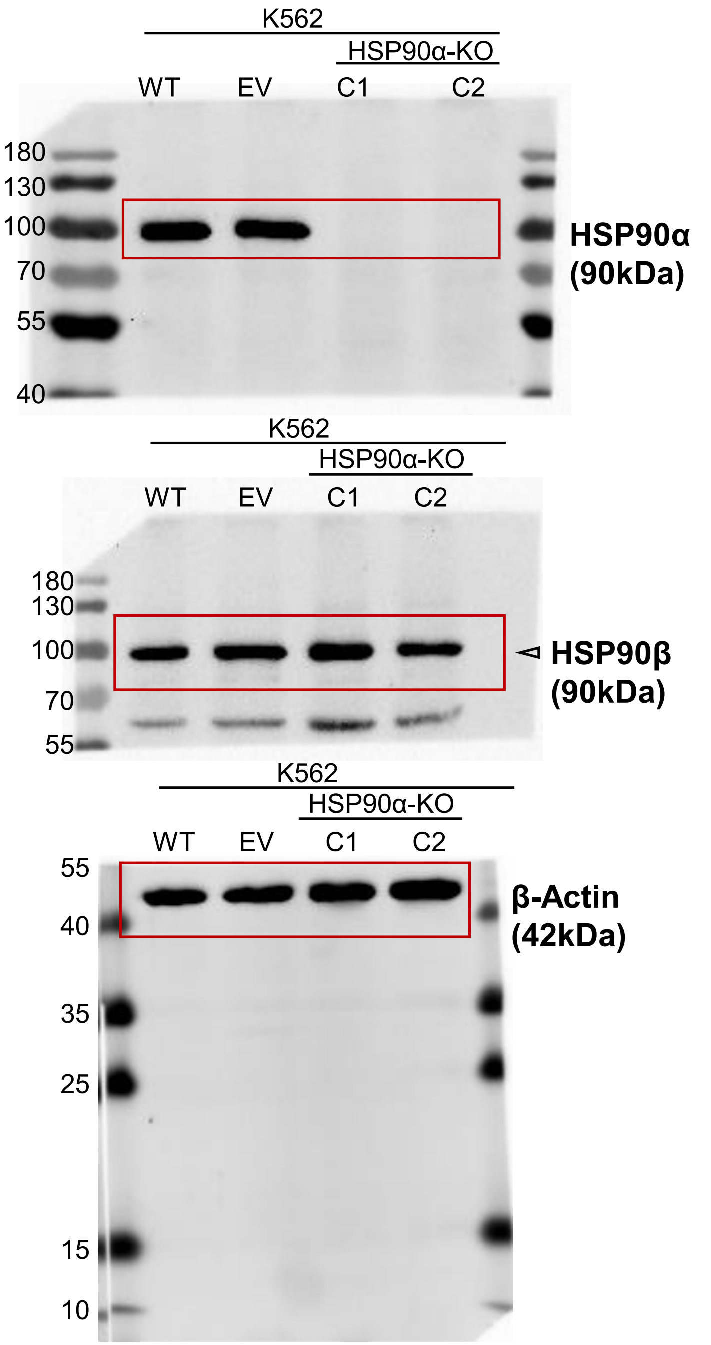

Main Figure 1A - right

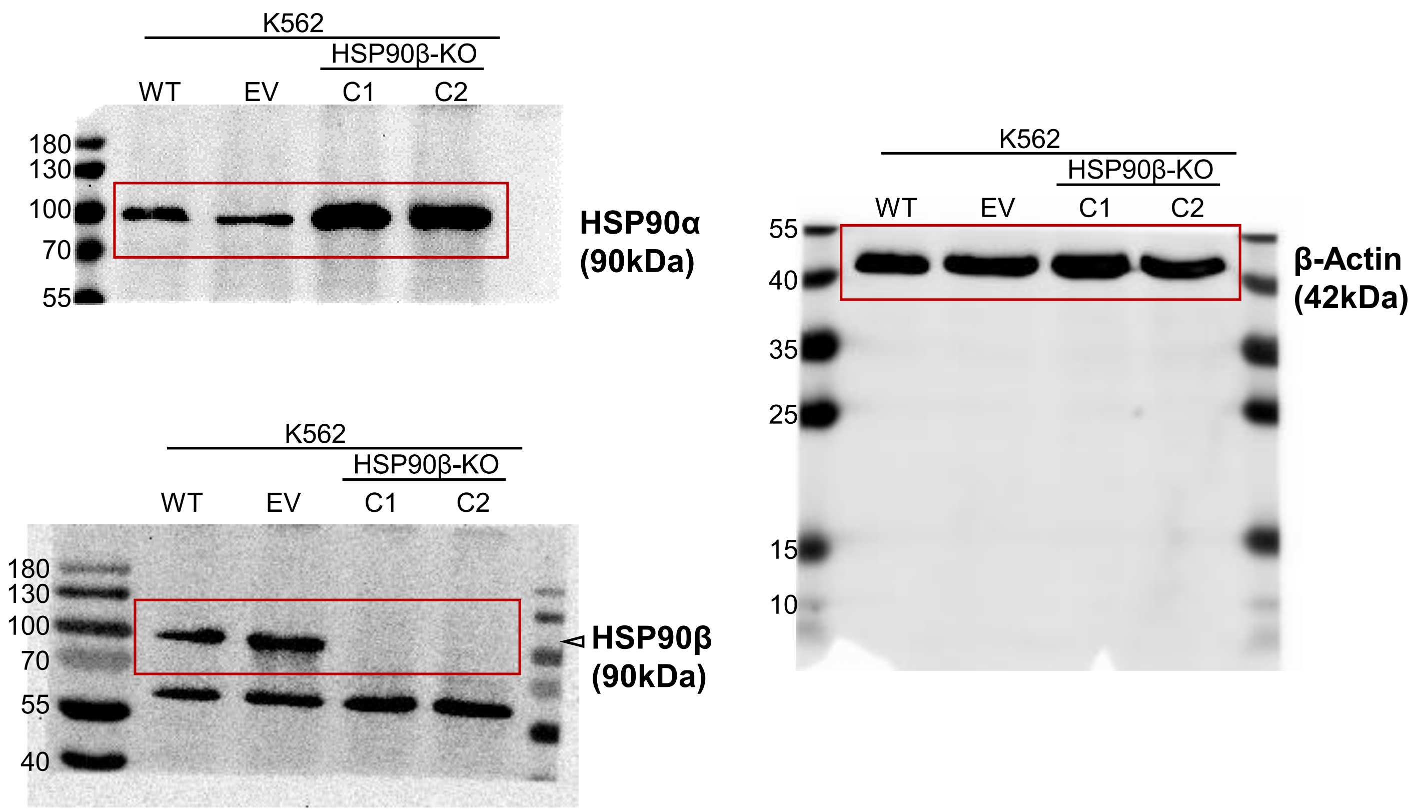

Main Figure 1B - left

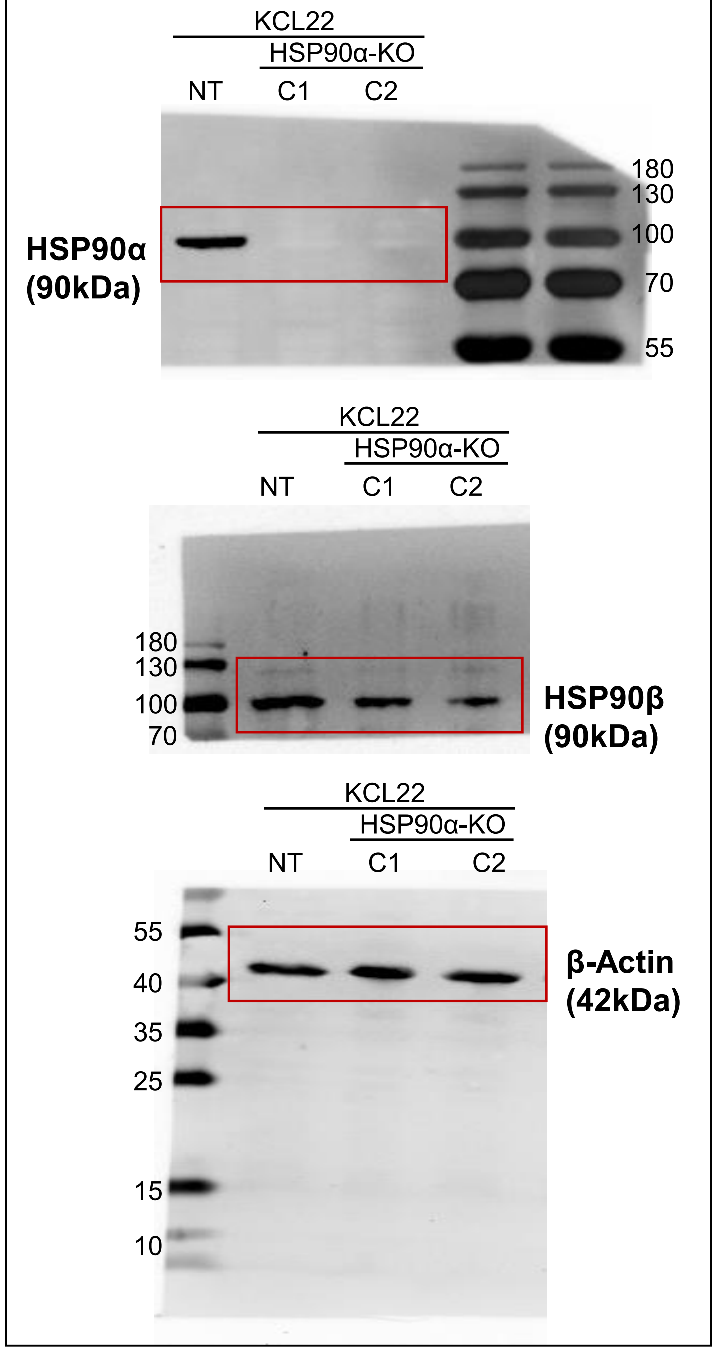

Main Figure 1B - right

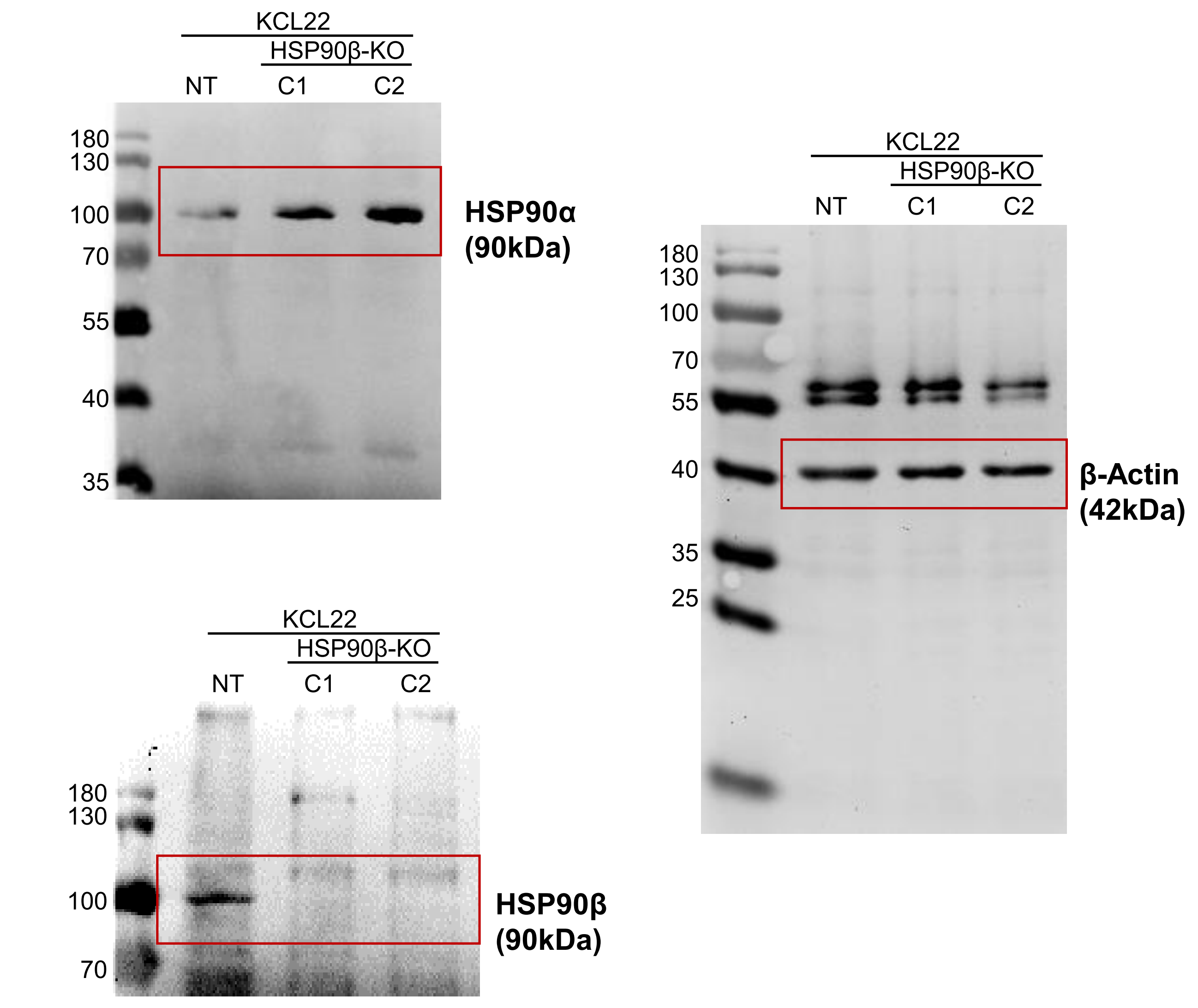

Main Figure 1C.1

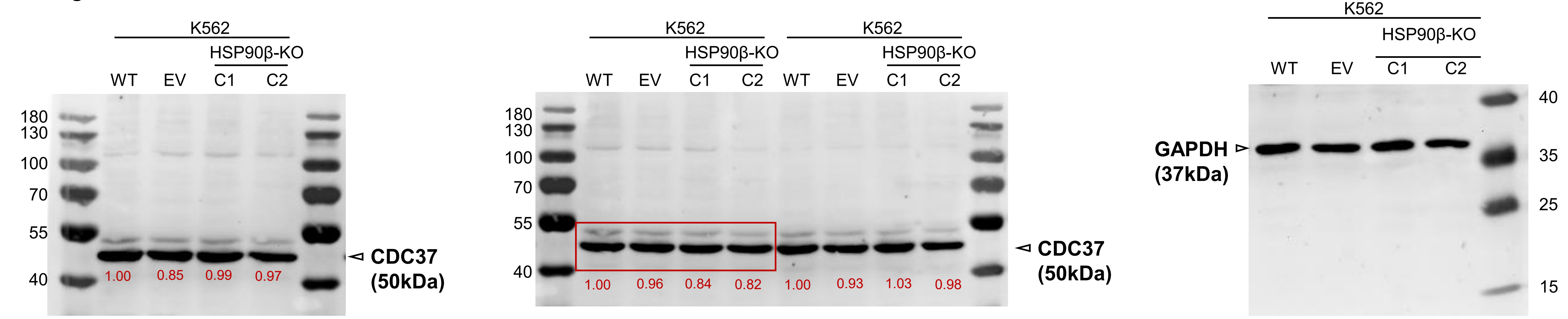

Main Figure 1C.2

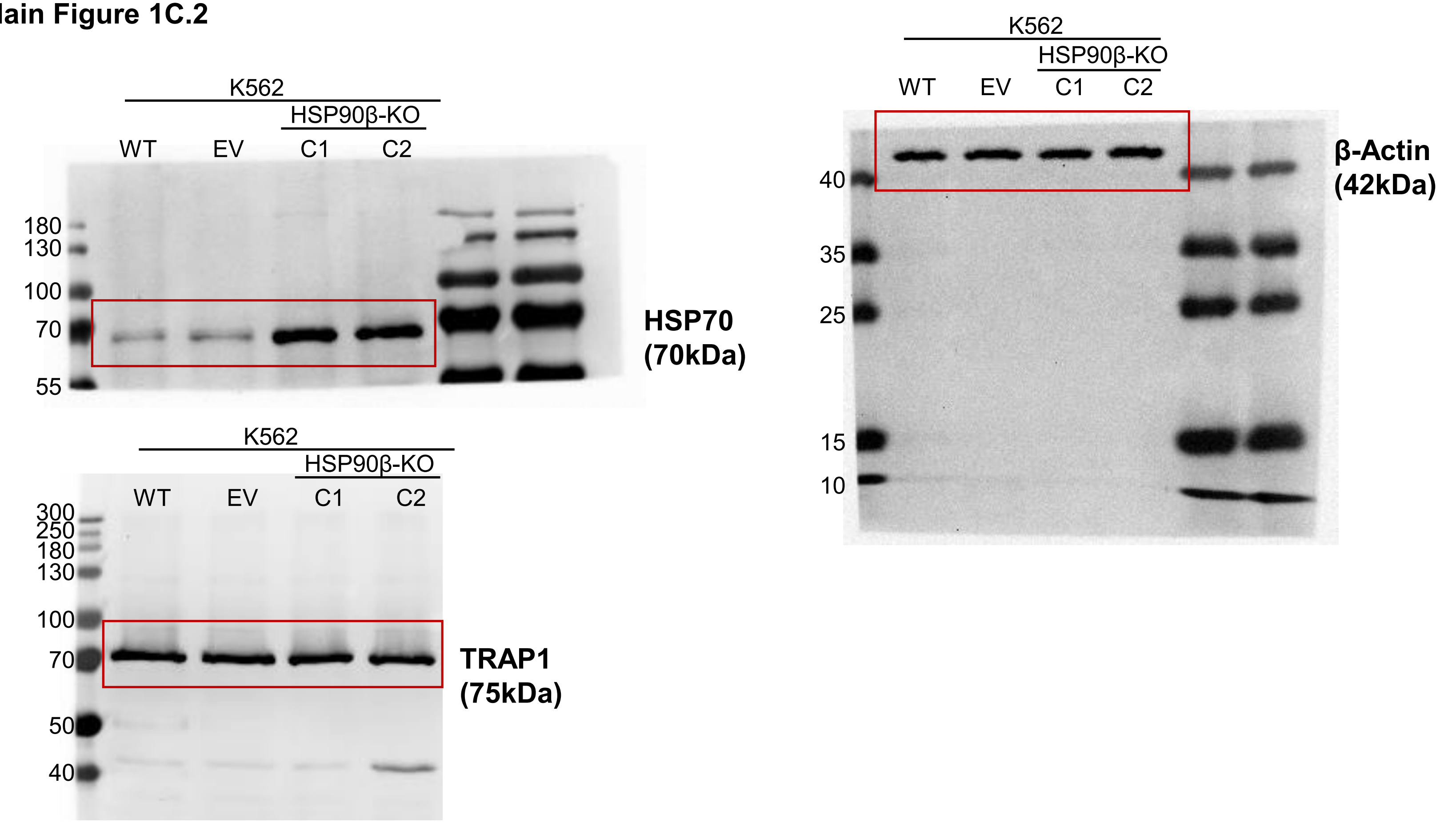

Main Figure 1C.3

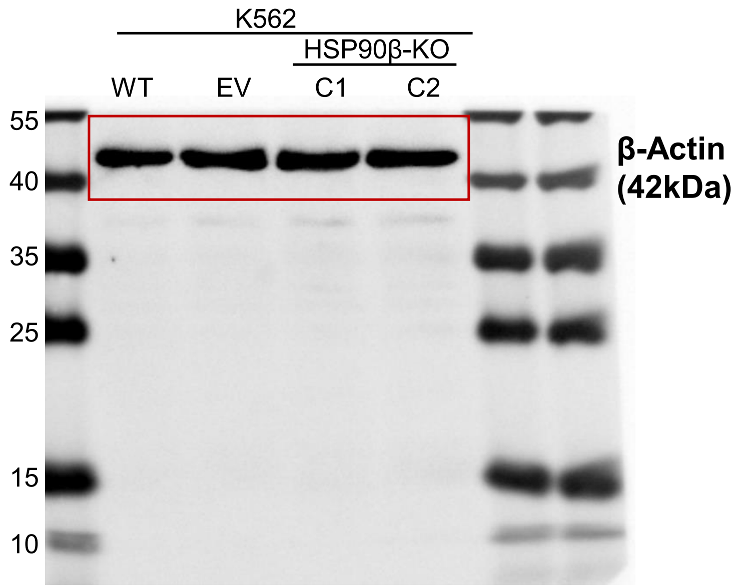

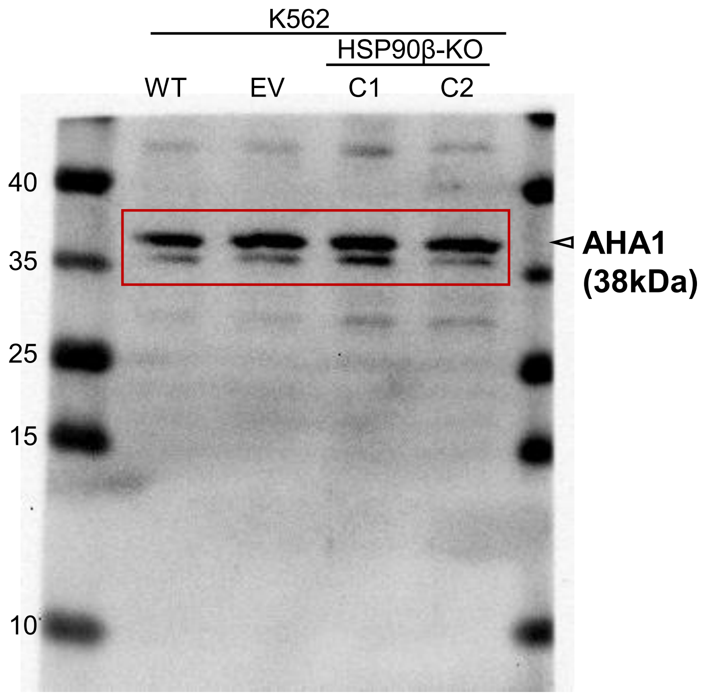

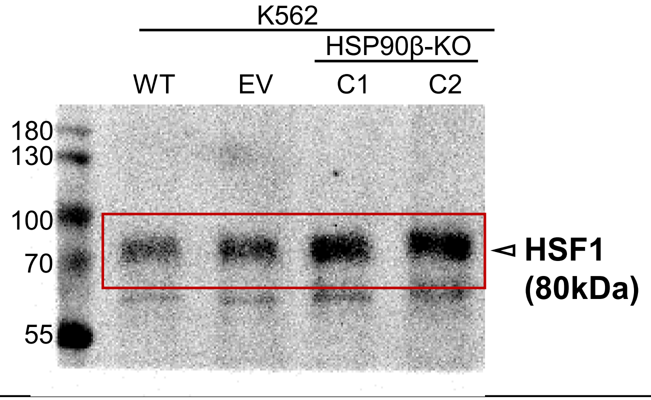

Main Figure 1C.4

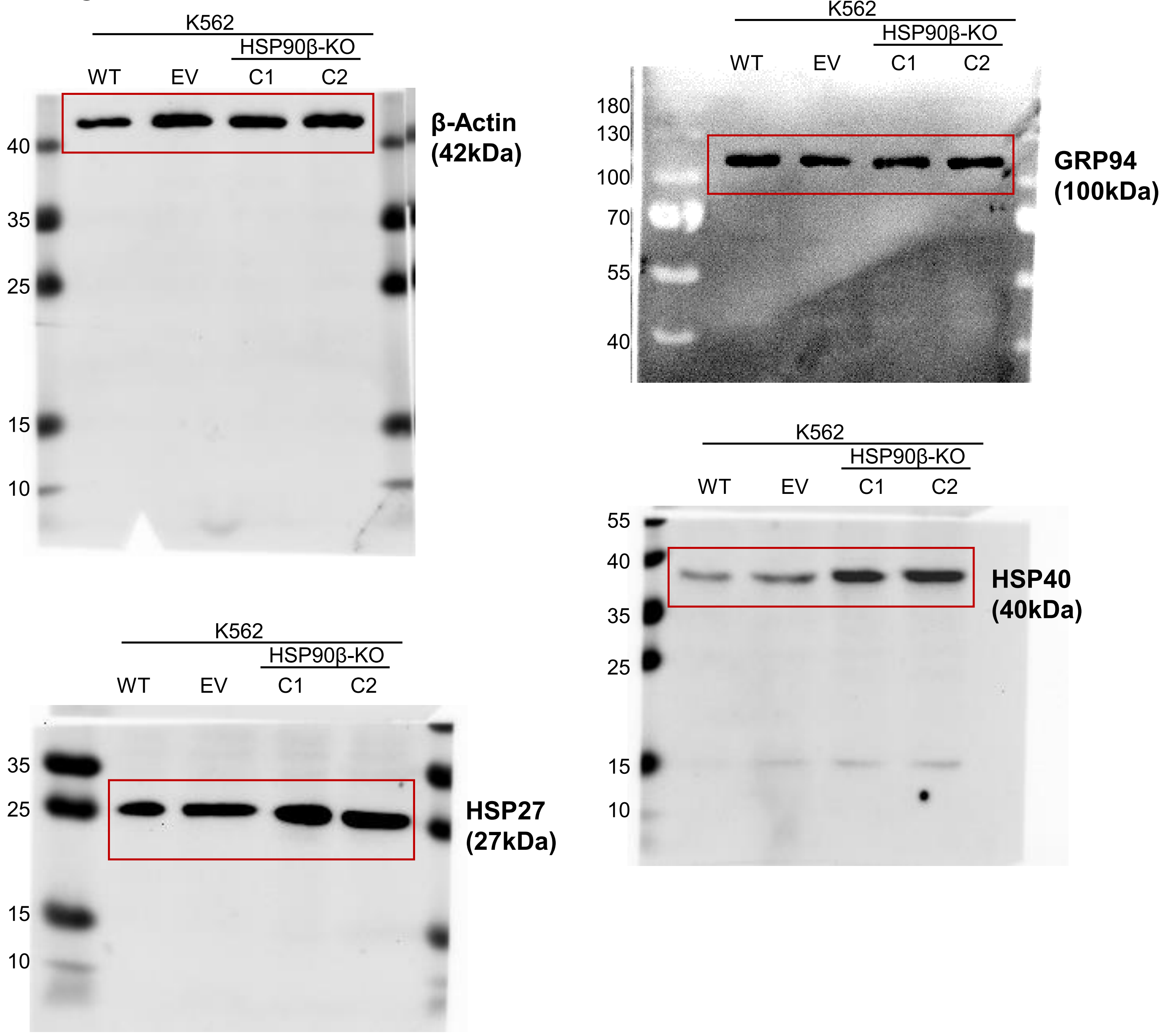

Main Figure 1D

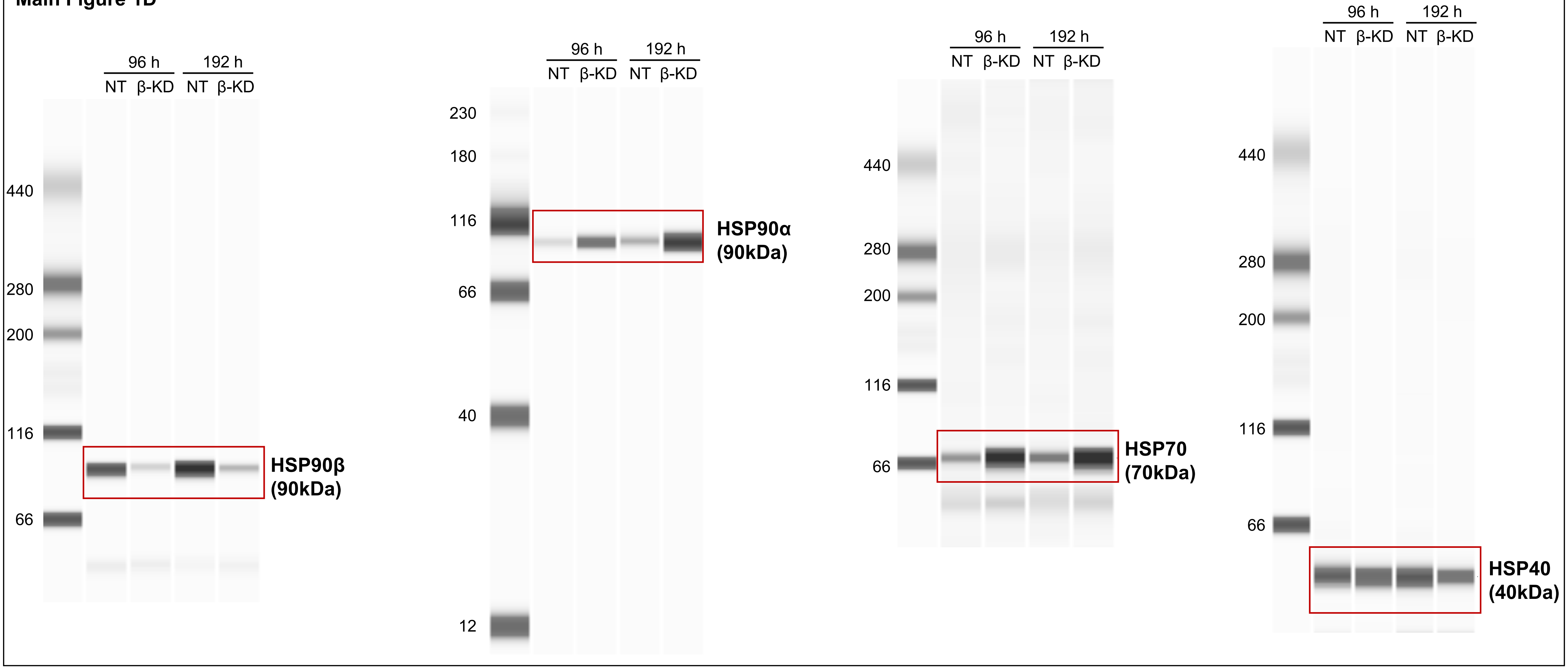

Main Figure 1D

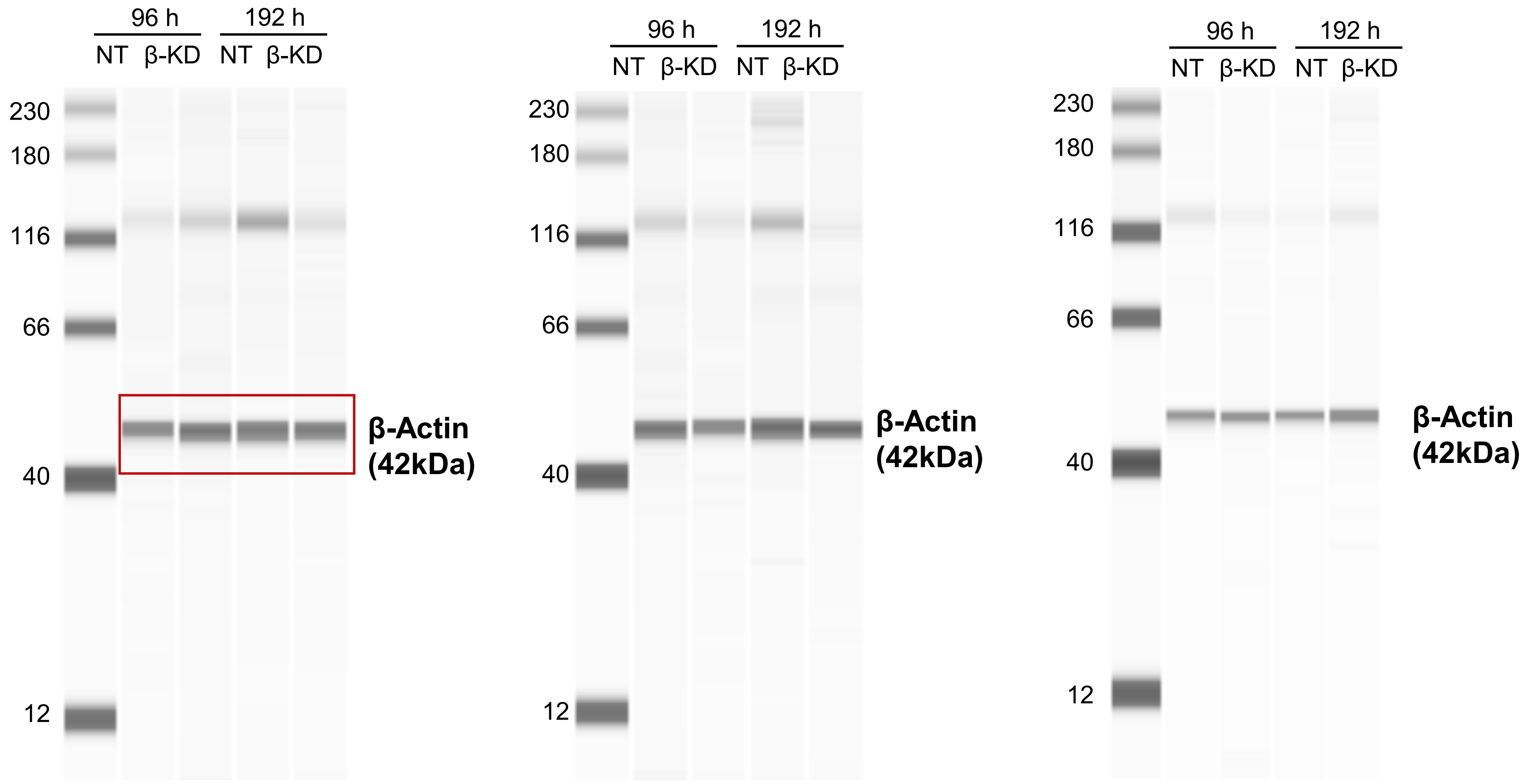

Main Figure 1D

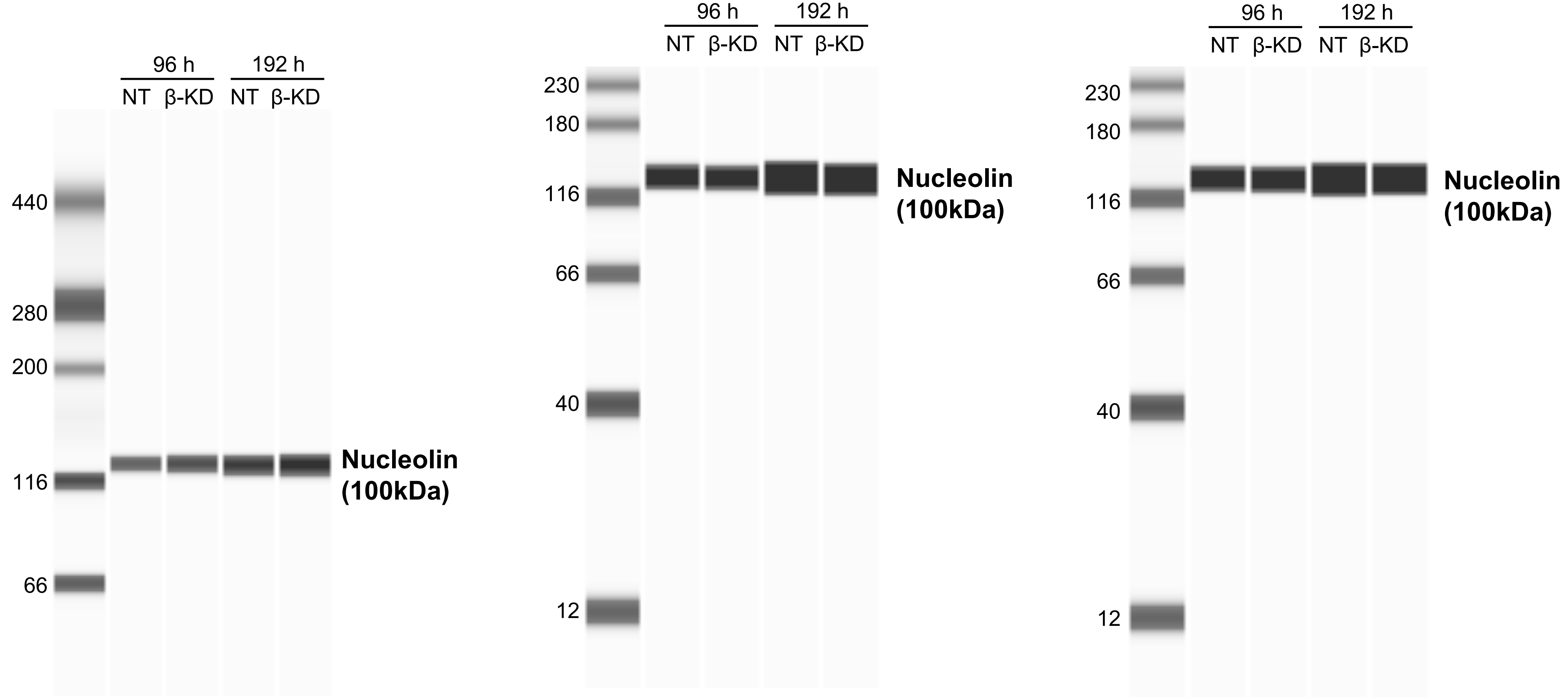

Suppl. Figure 1F.1

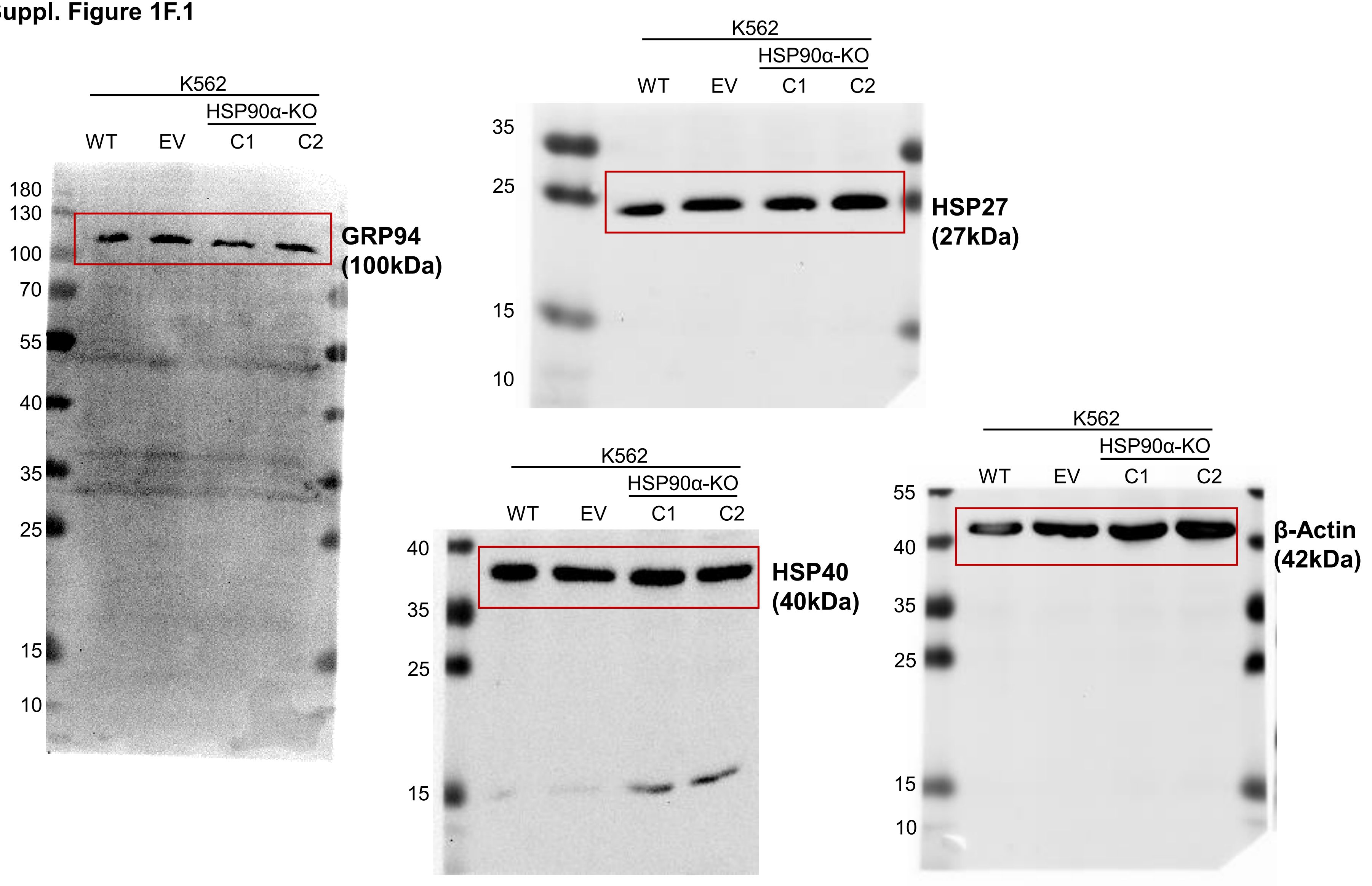

Suppl. Figure 1F.2

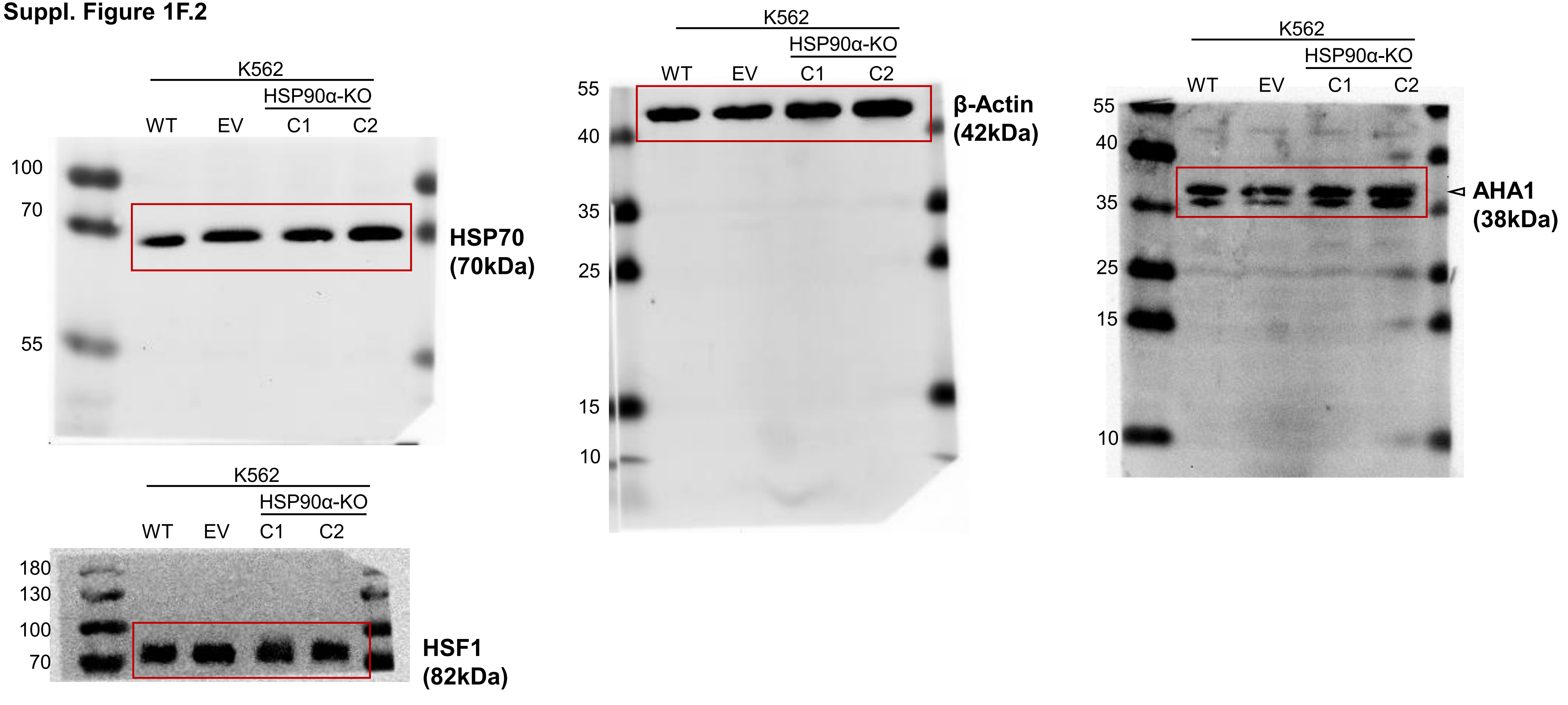

Suppl. Figure 1F.3

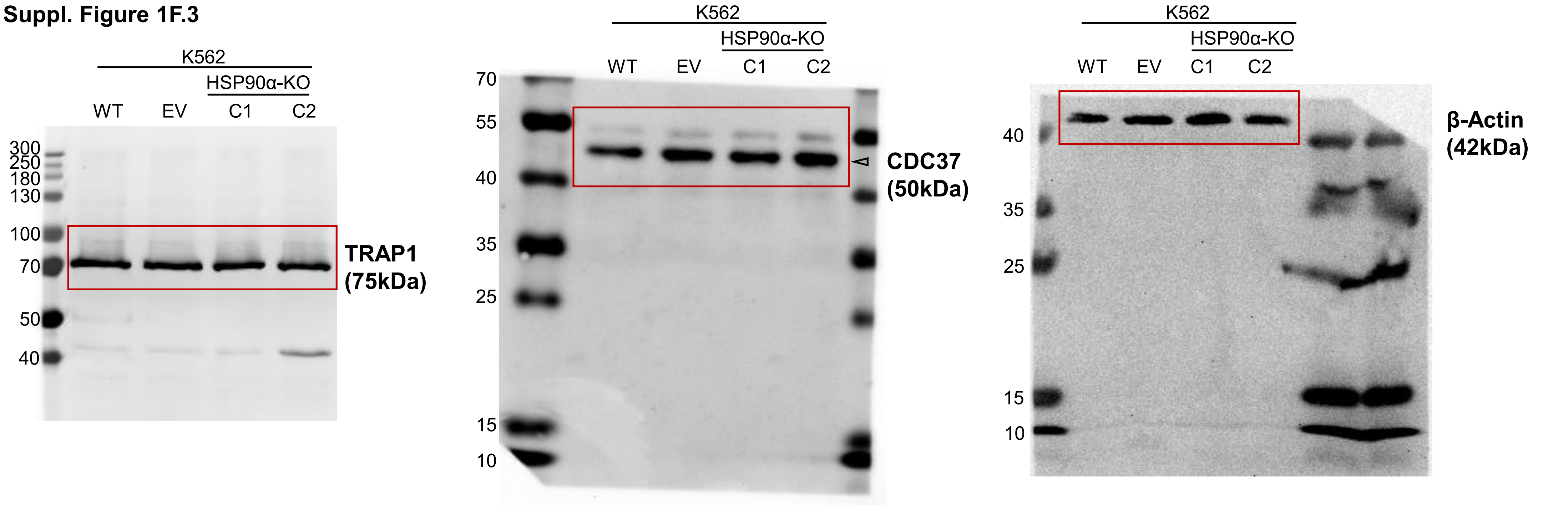

Suppl. Figure 1G

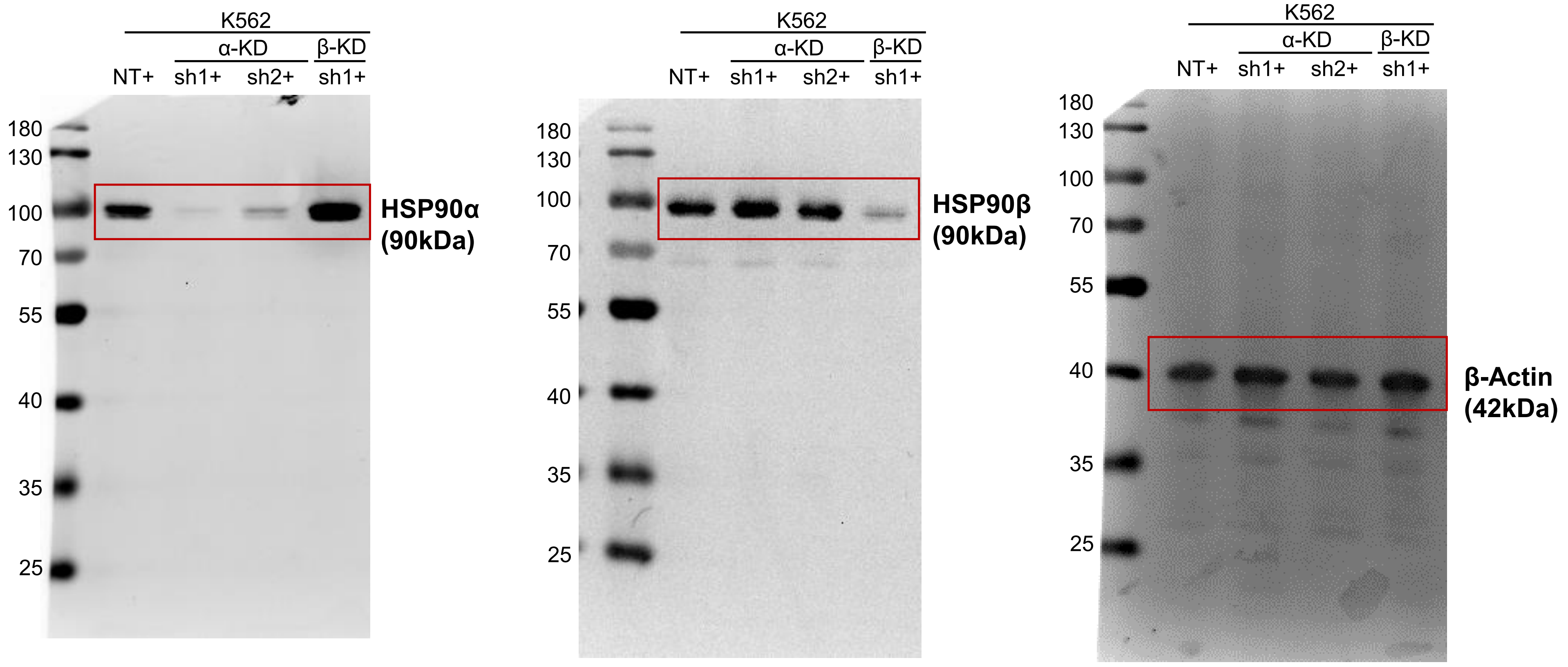

Suppl. Figure 1I

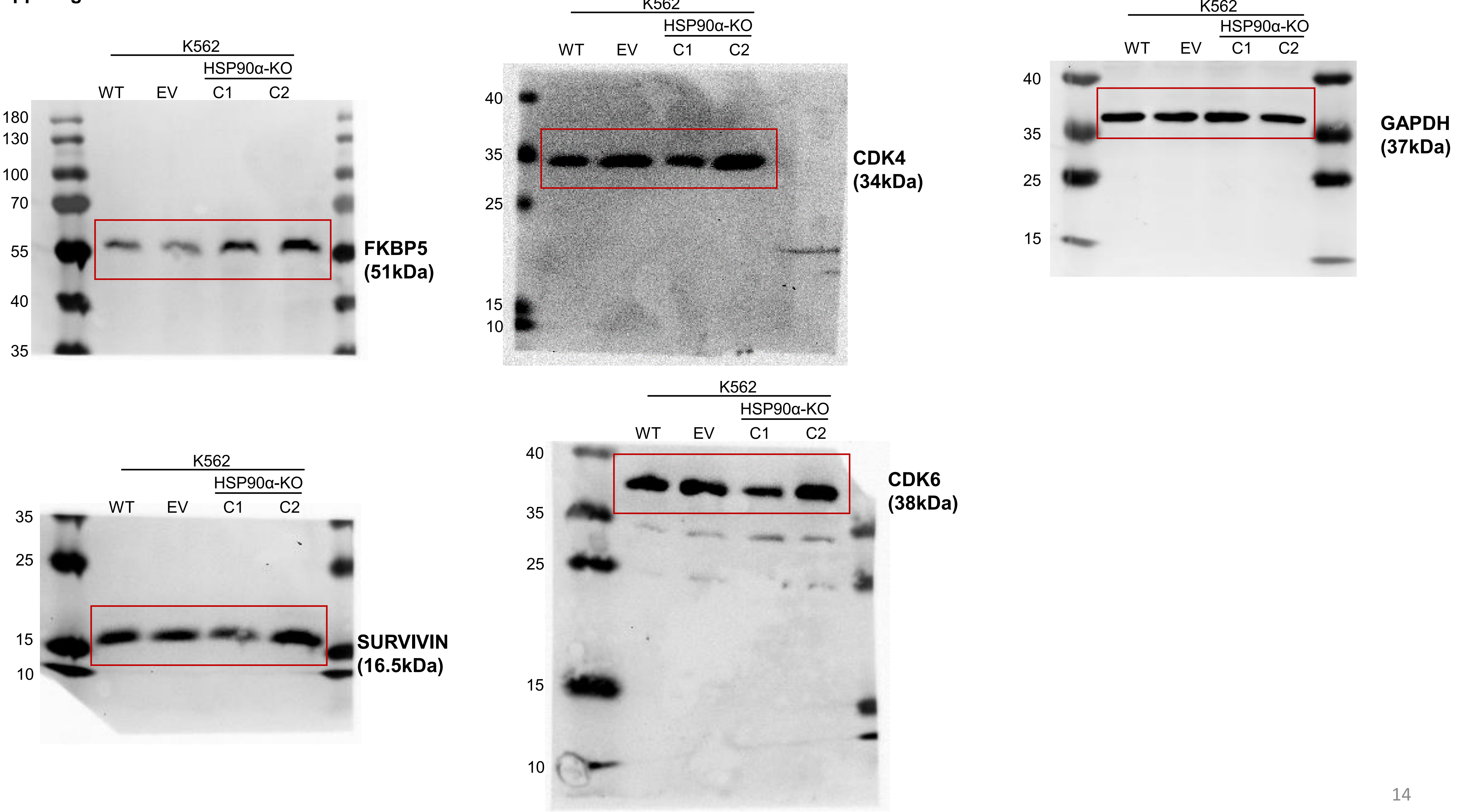

Suppl. Figure 1J

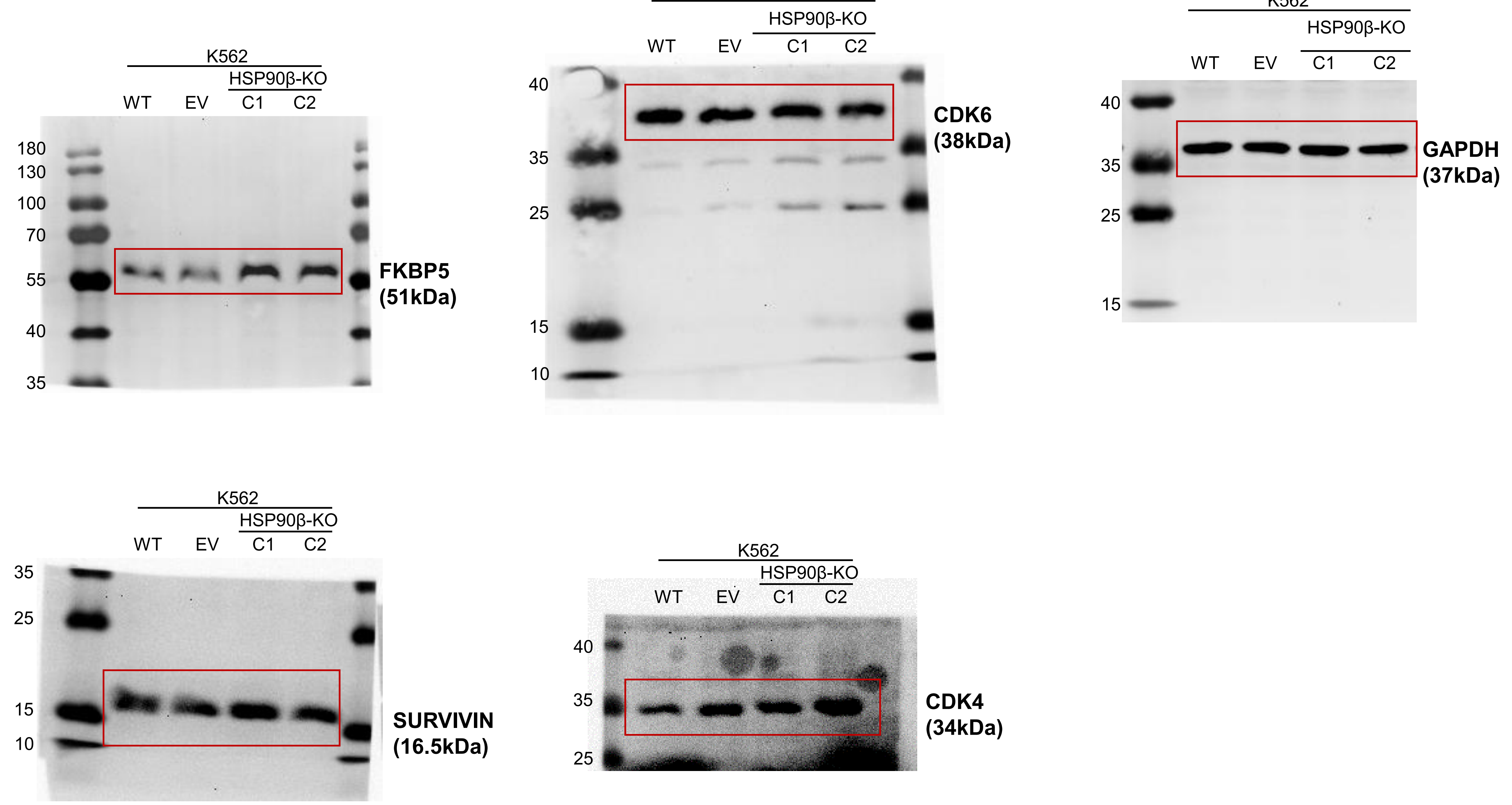

Suppl. Figure 1K

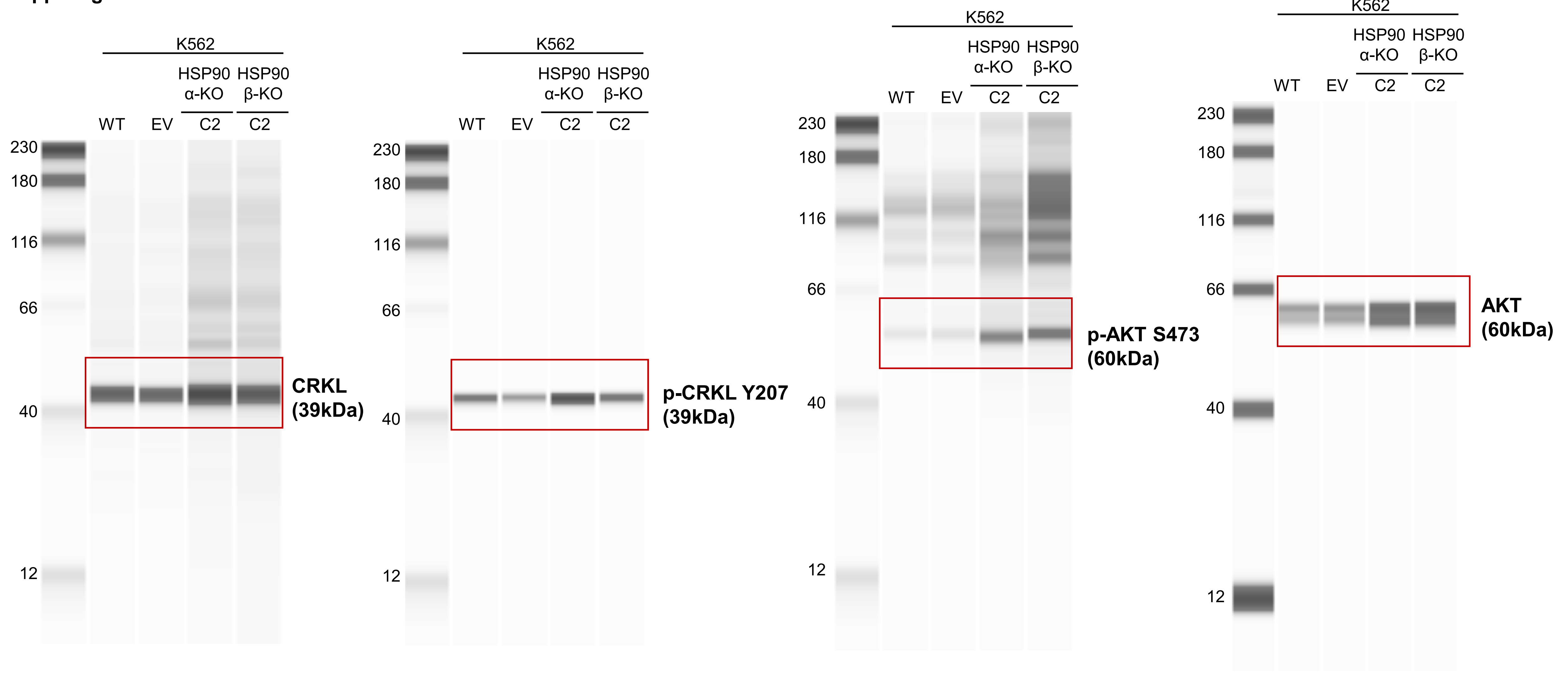

Suppl. Figure 1K

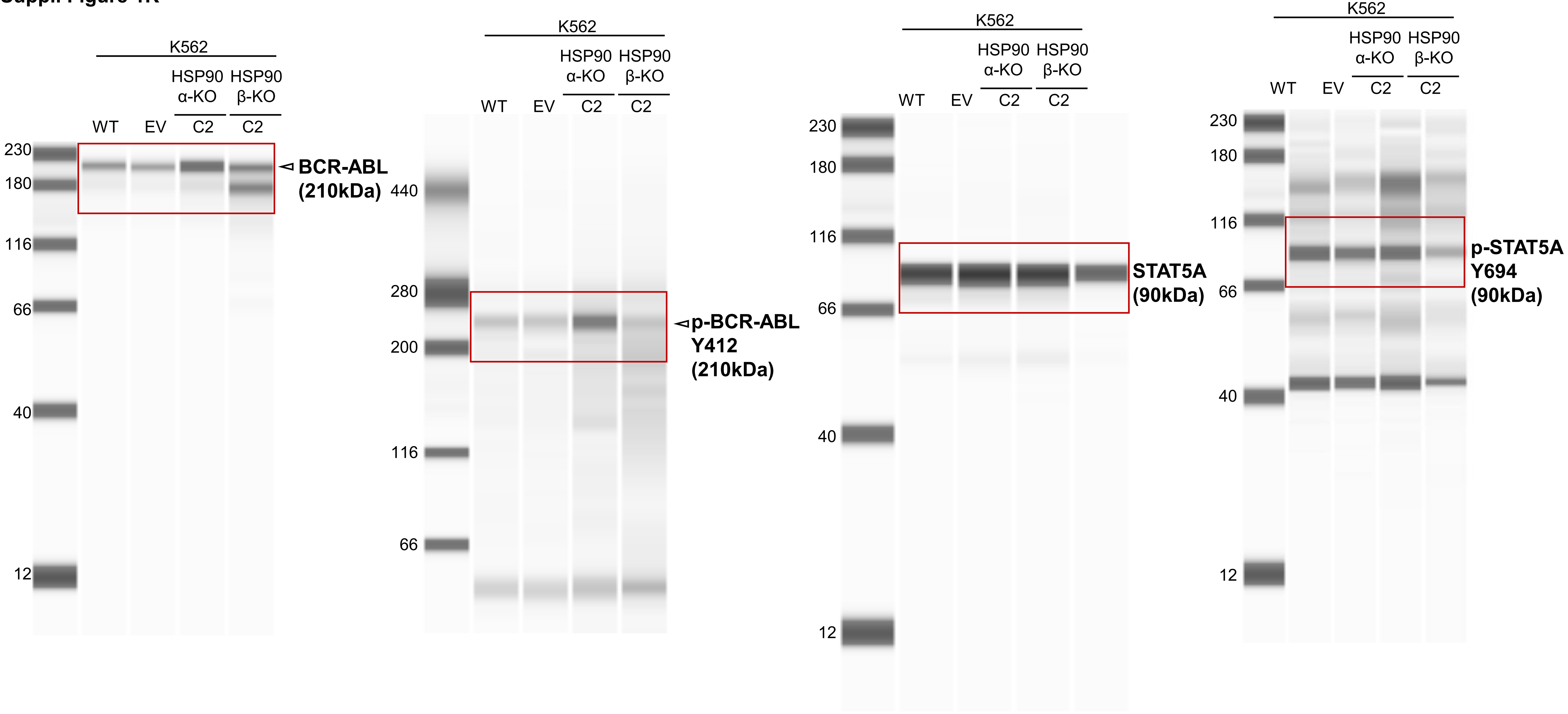

Suppl. Figure 1K.replicates

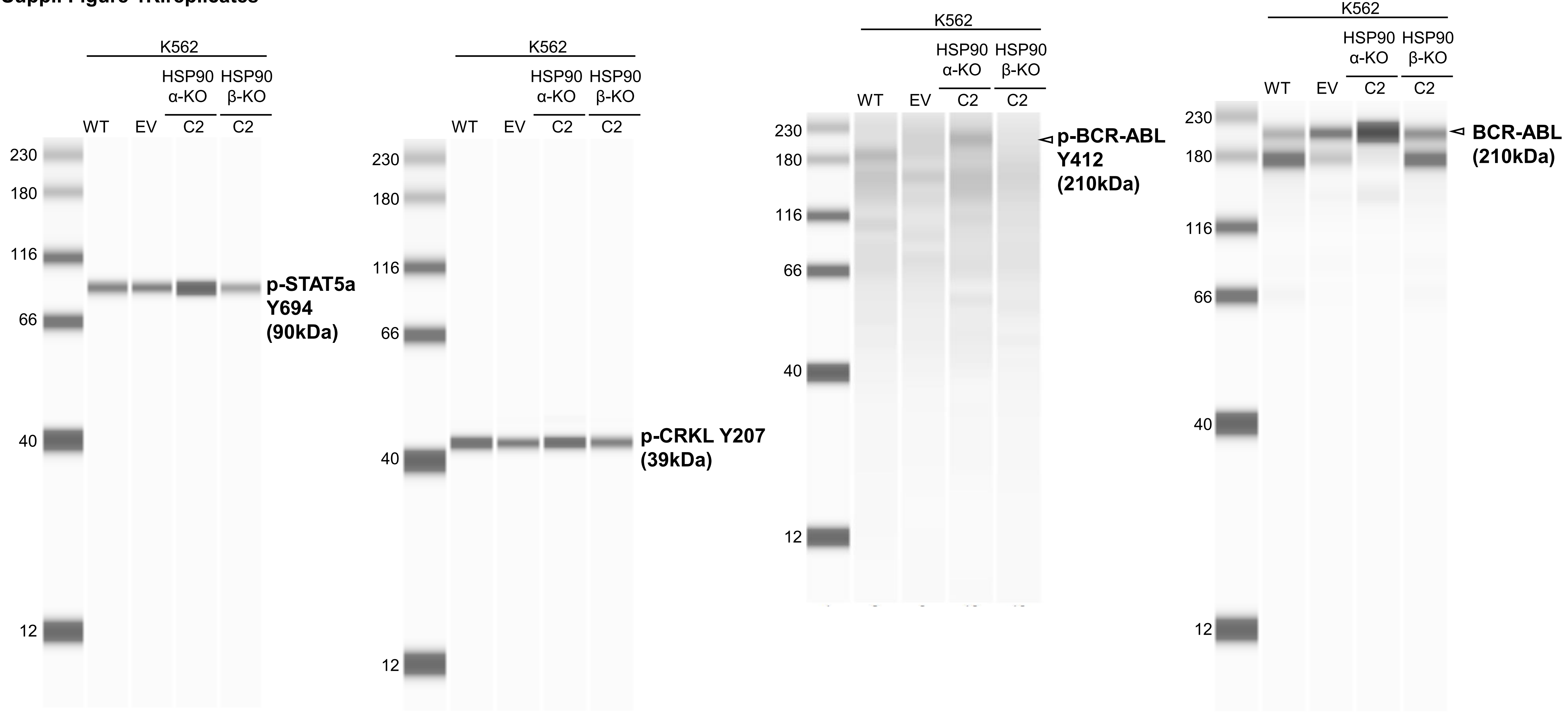

Suppl. Figure 1K.replicates

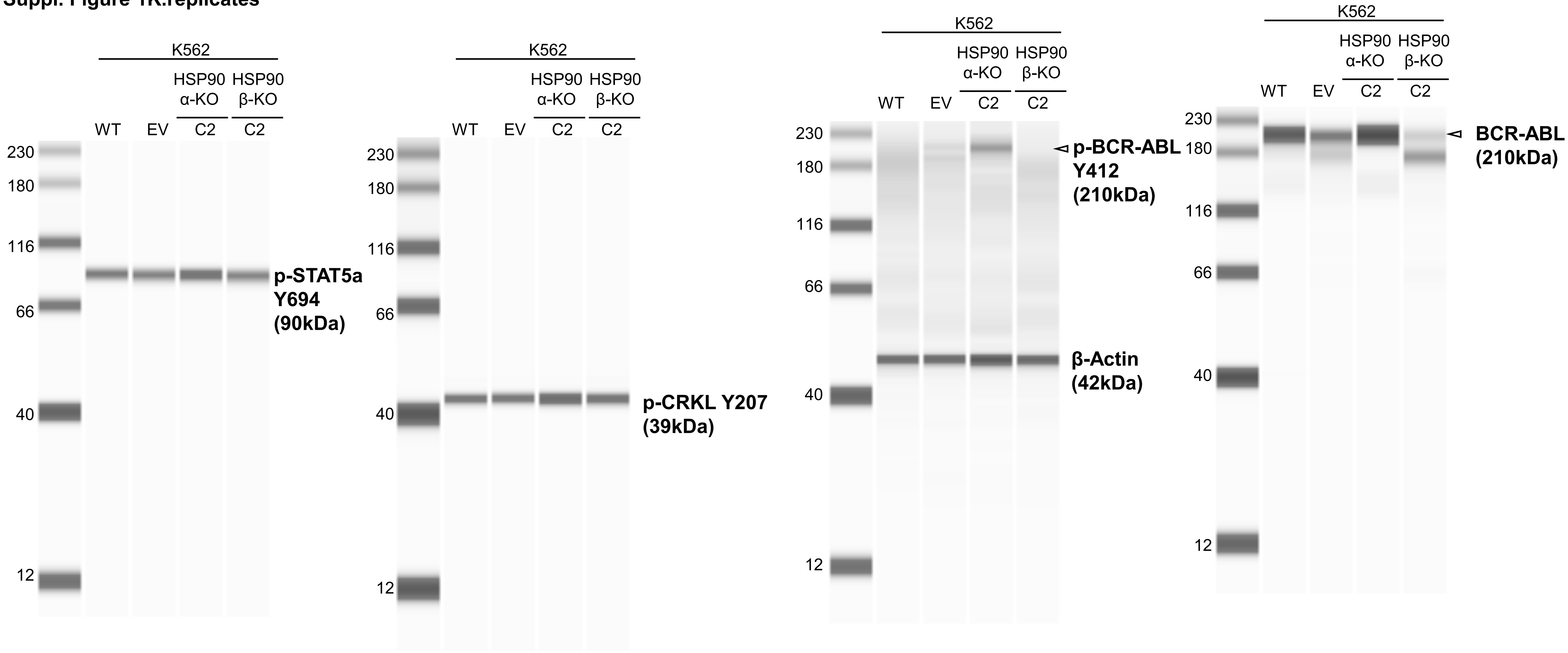

Suppl. Figure 1K

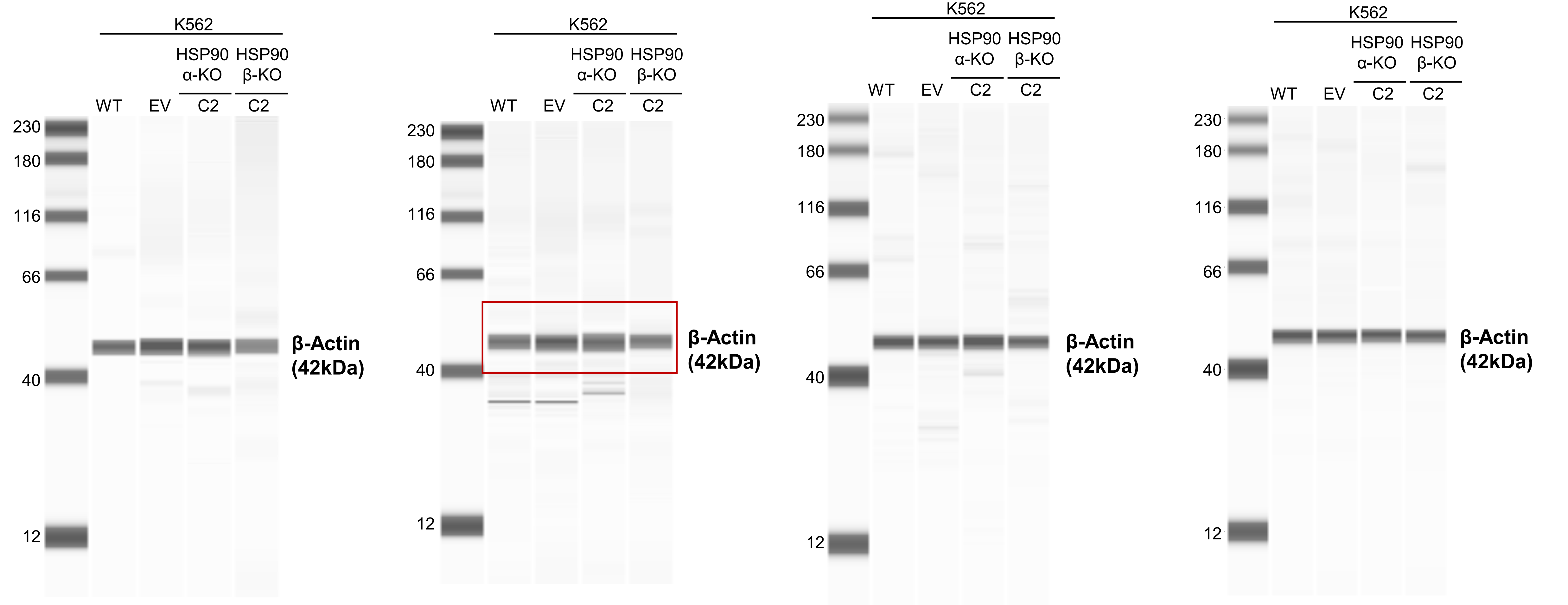

Main Figure 3D

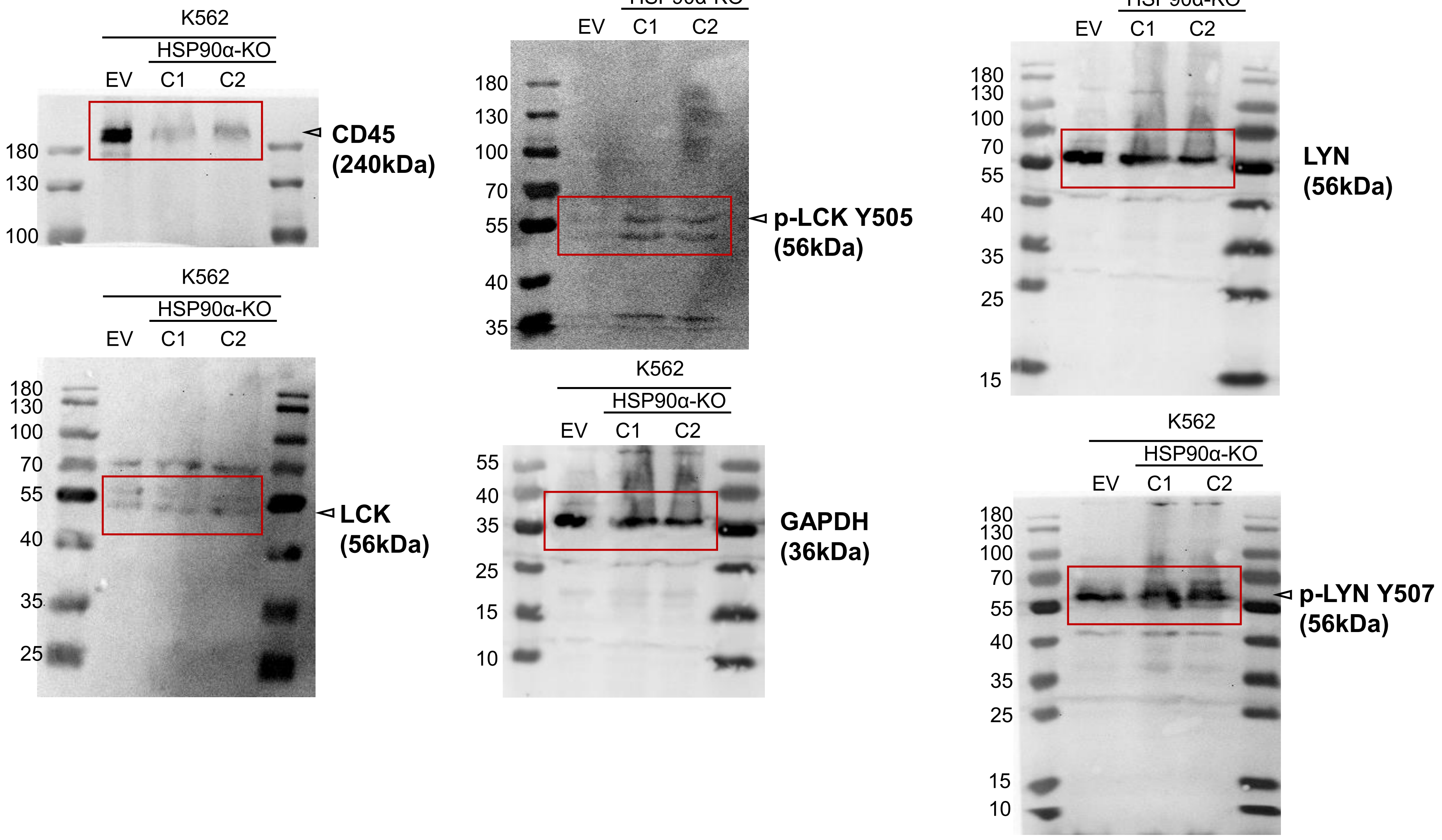

Main Figure 3F

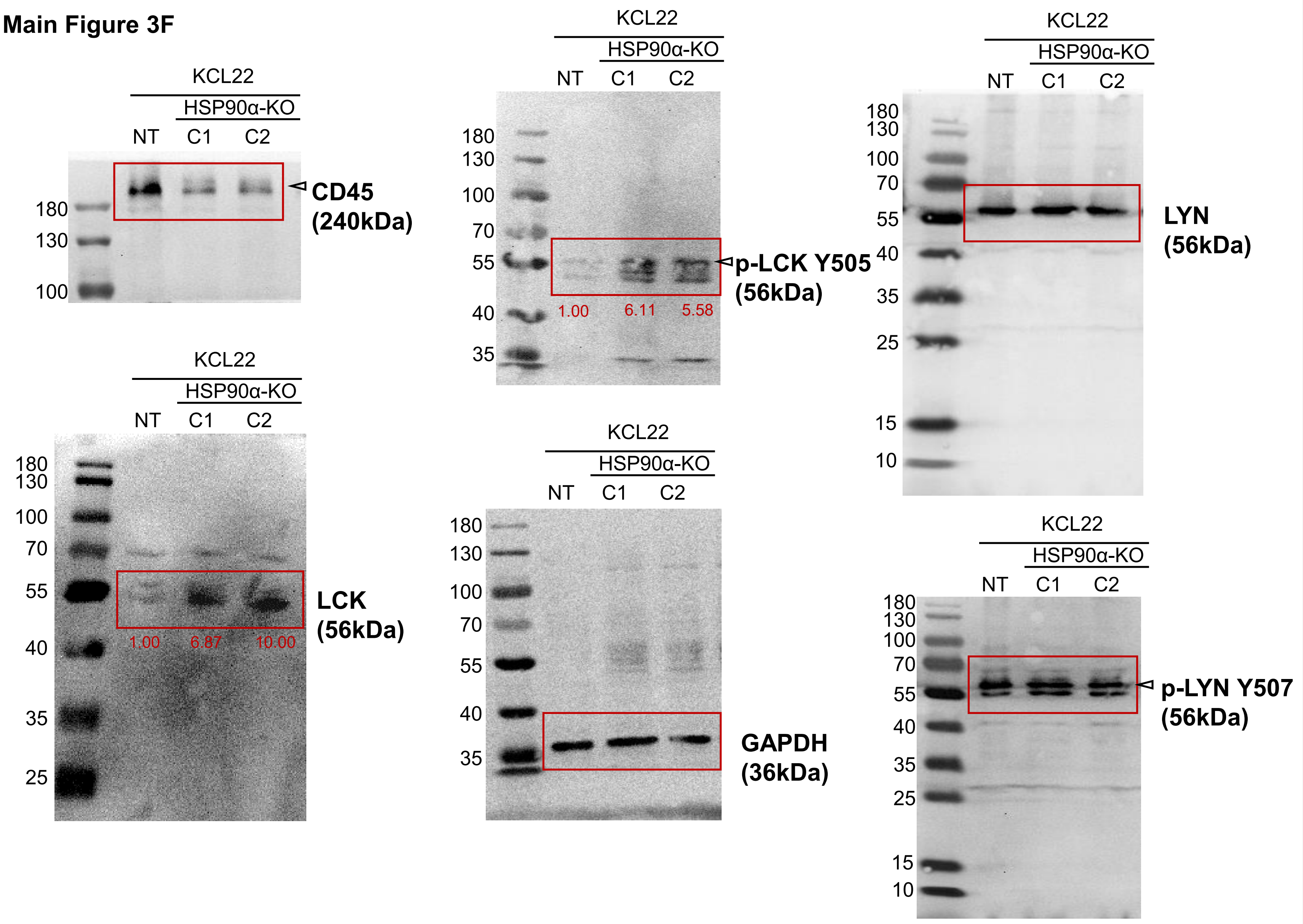

Main Figure 3F.replicates

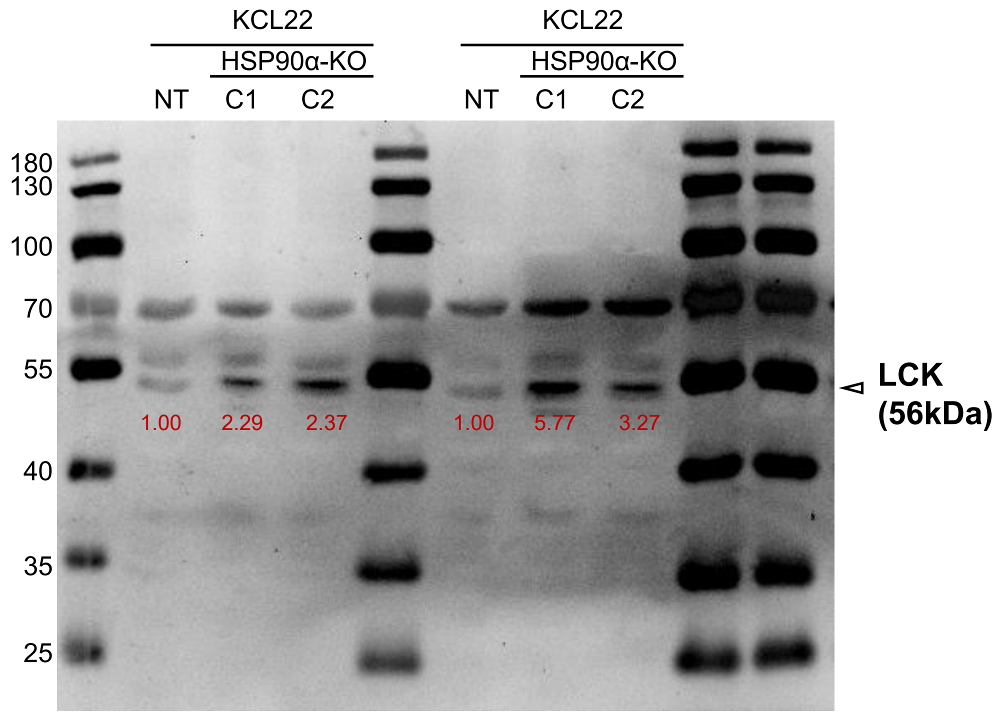

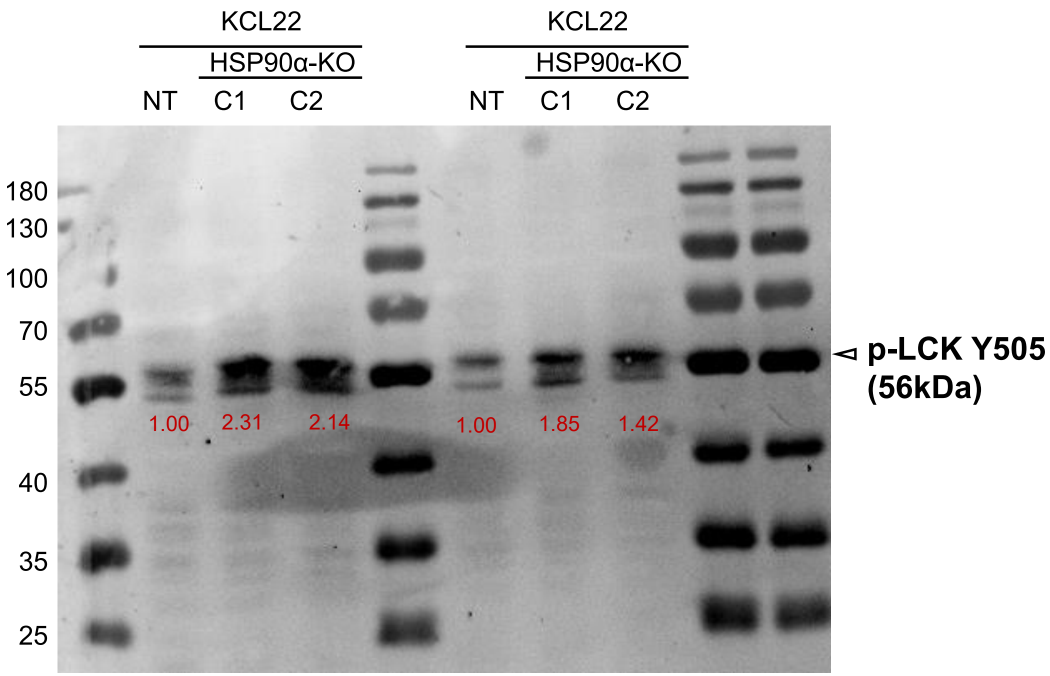

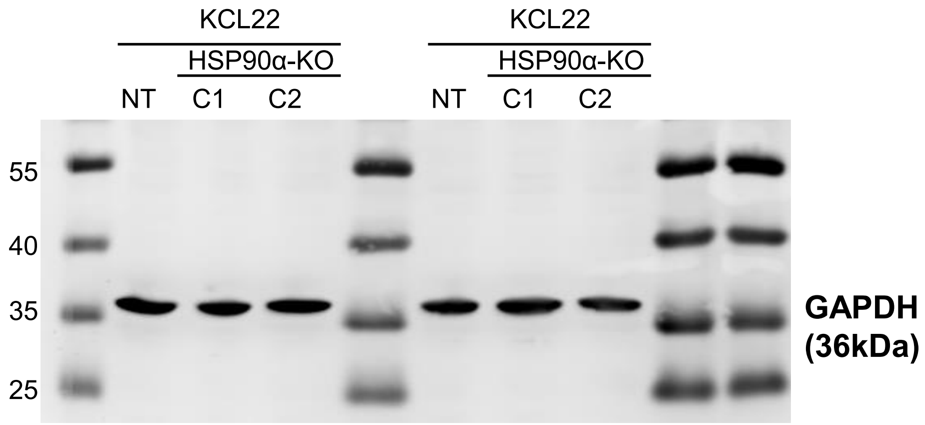

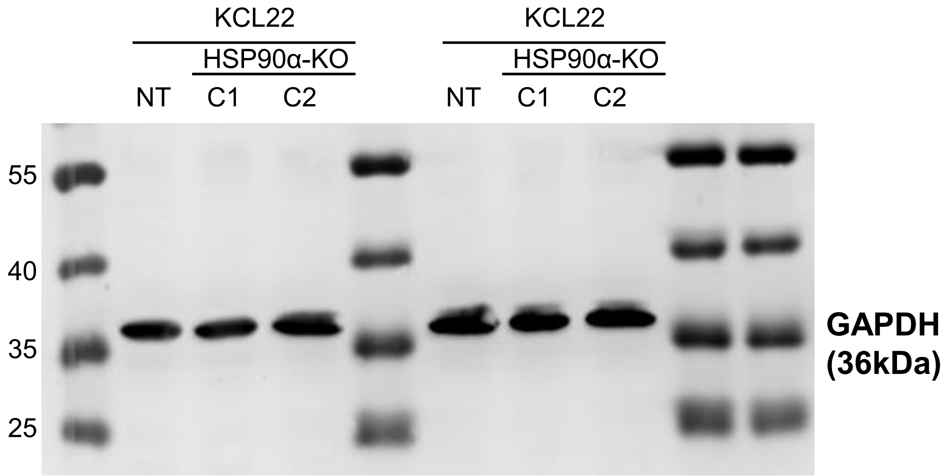

Main Figure 3G

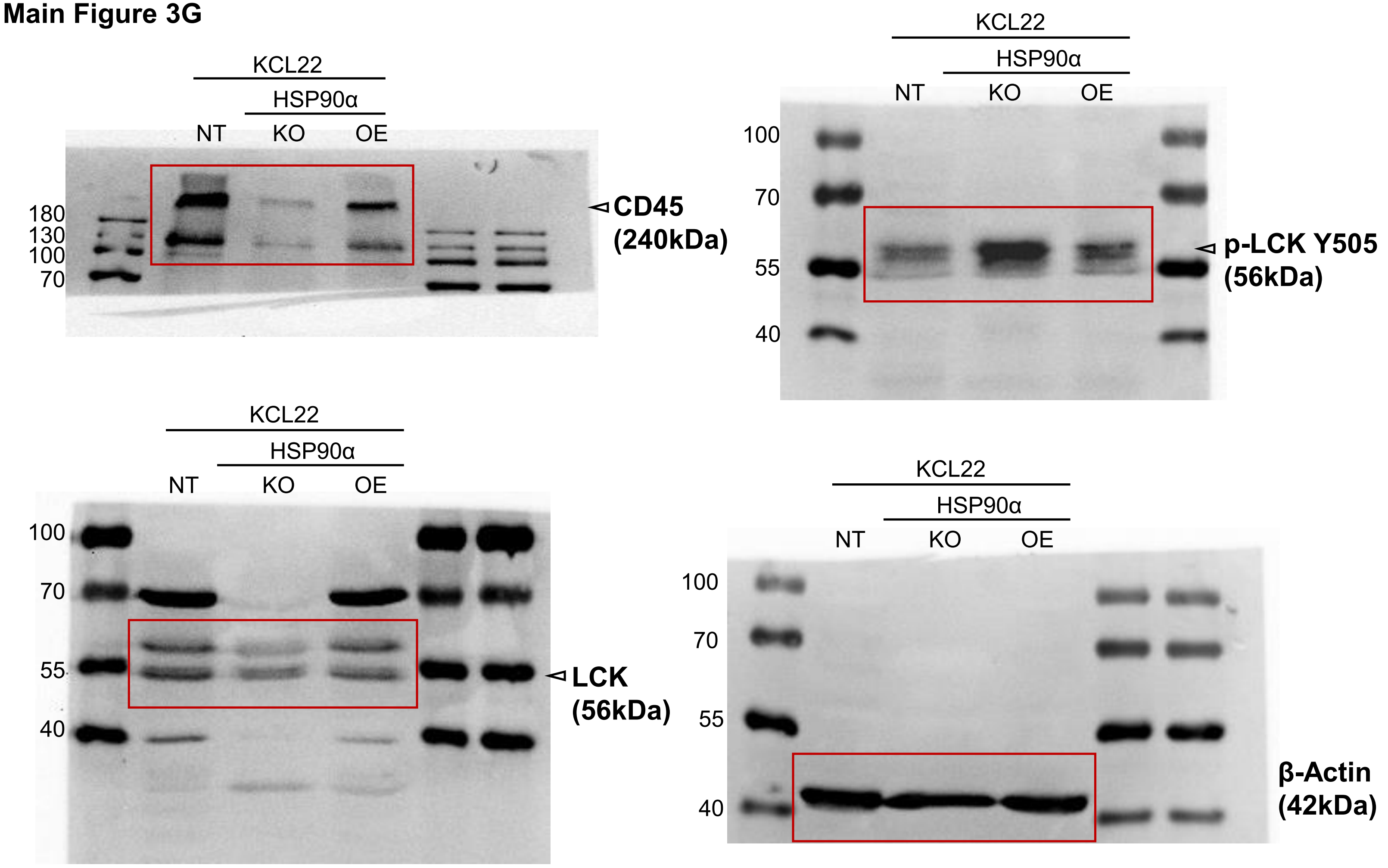

Suppl. Figure 3B.1

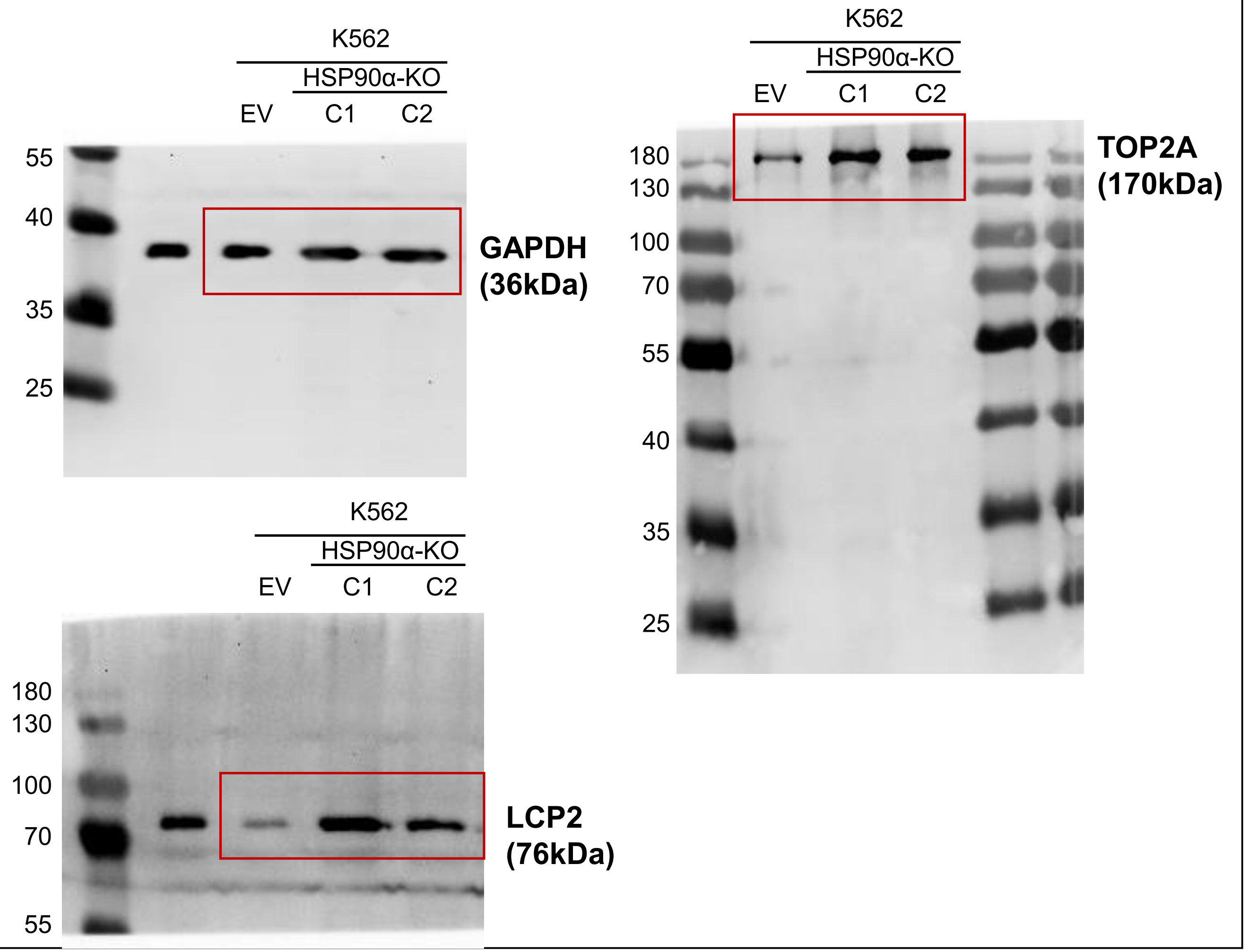

Suppl. Figure 3B.2

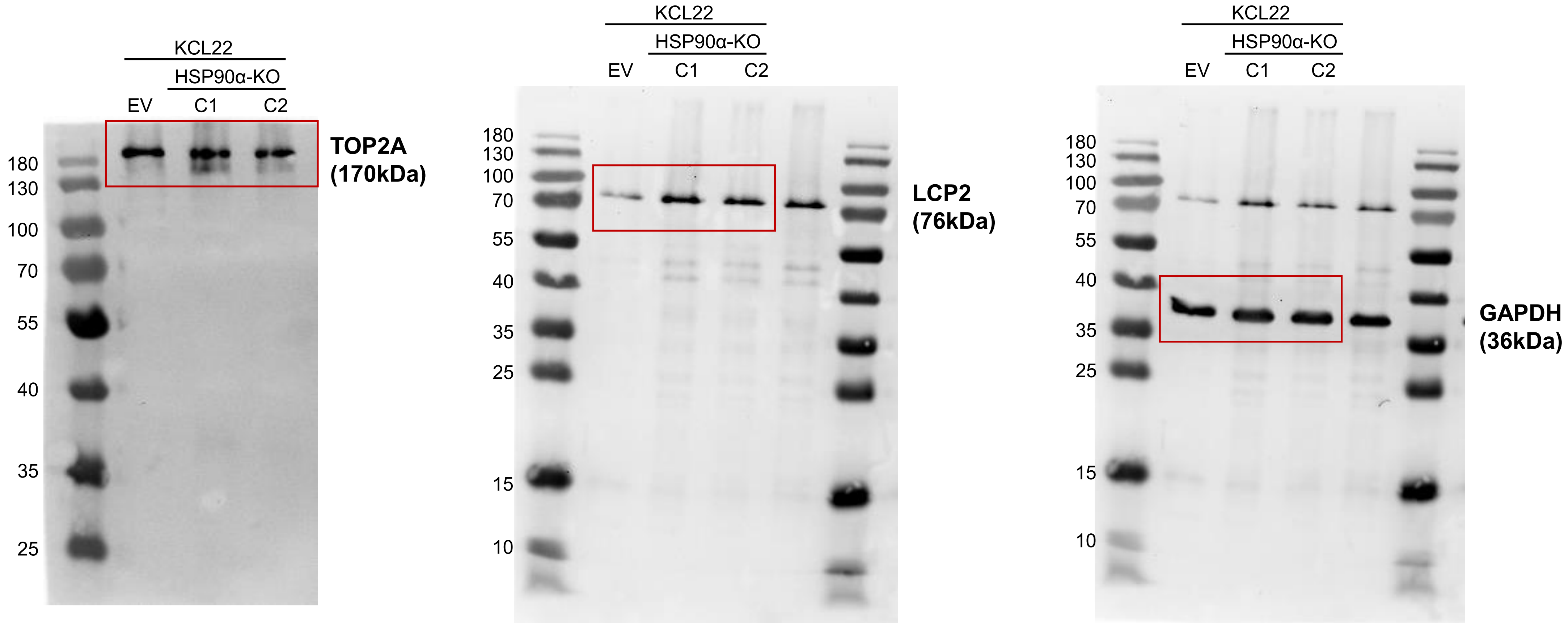

Suppl. Figure 3B.3

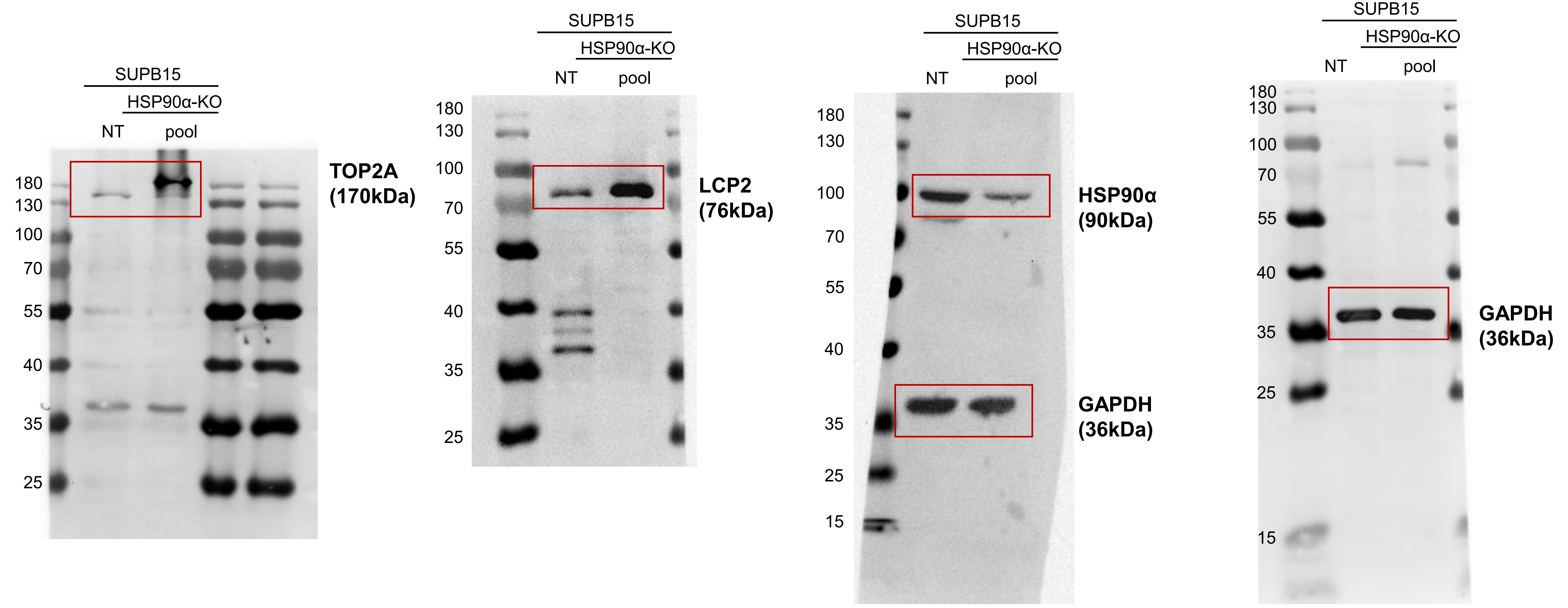

Suppl. Figure 3D

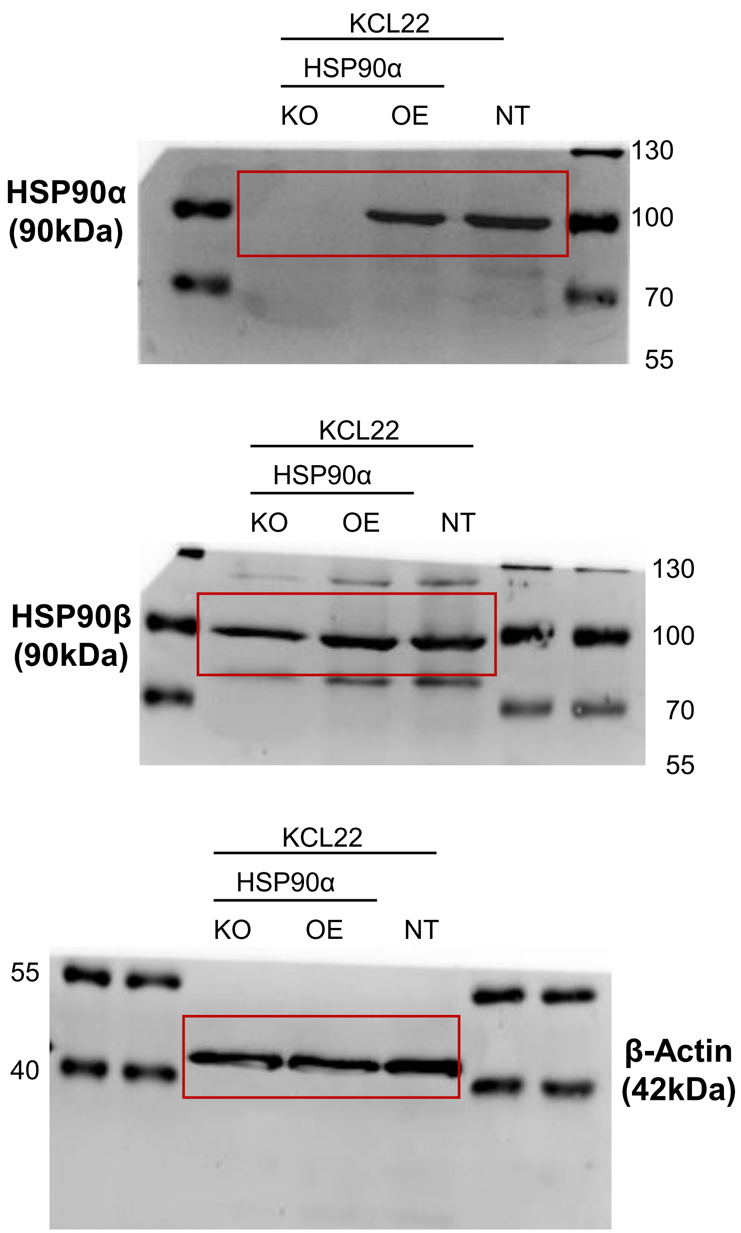

Main Figure 4D.1

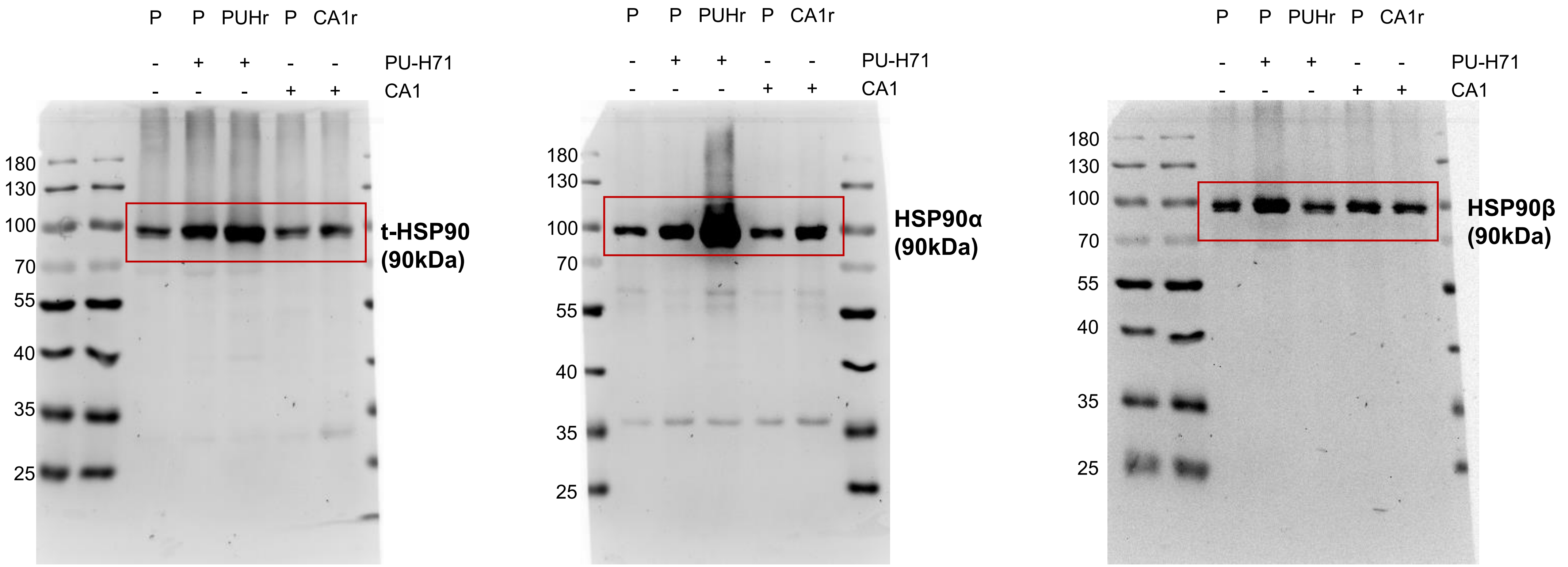

Main Figure 4D.2

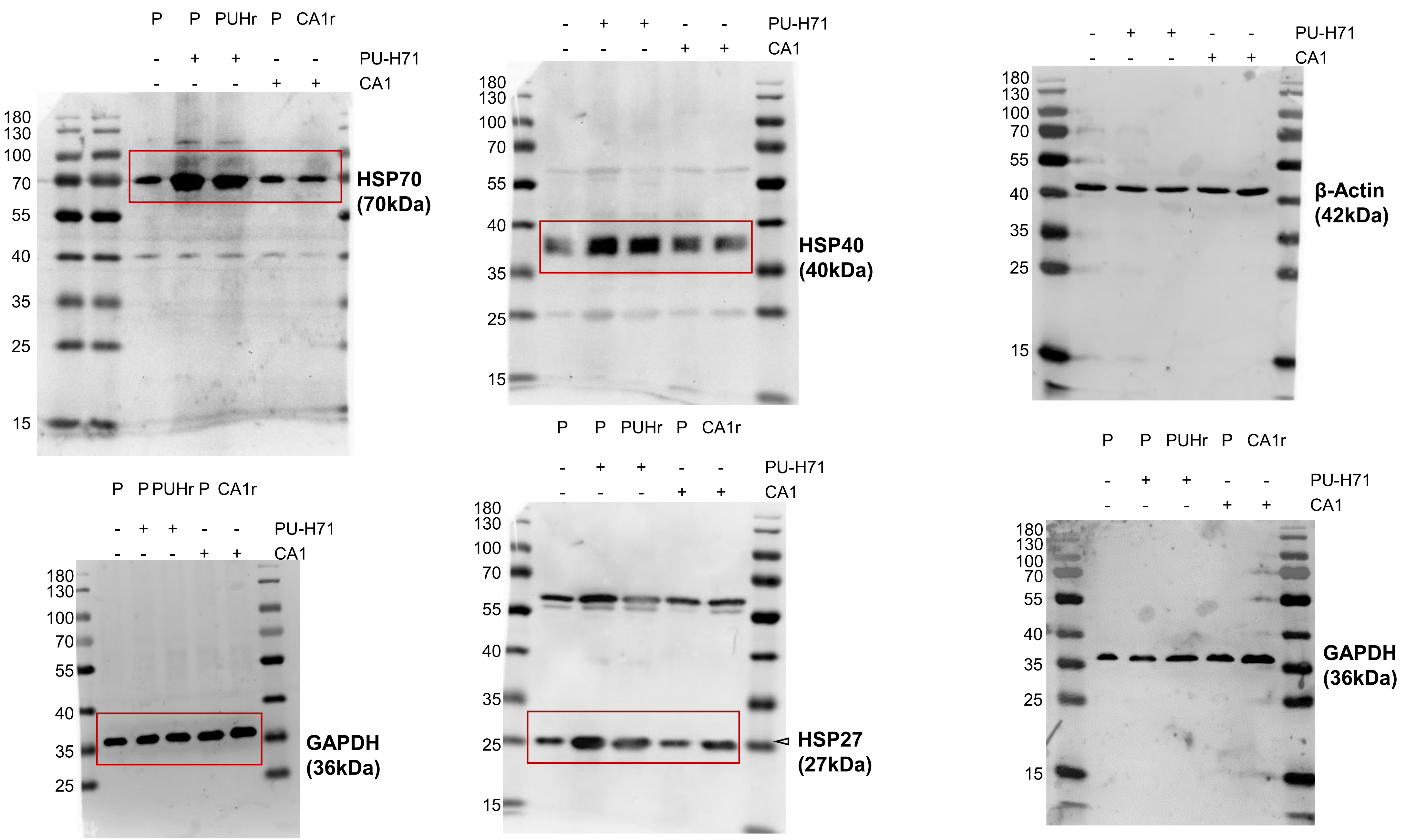

Suppl. Figure 4C

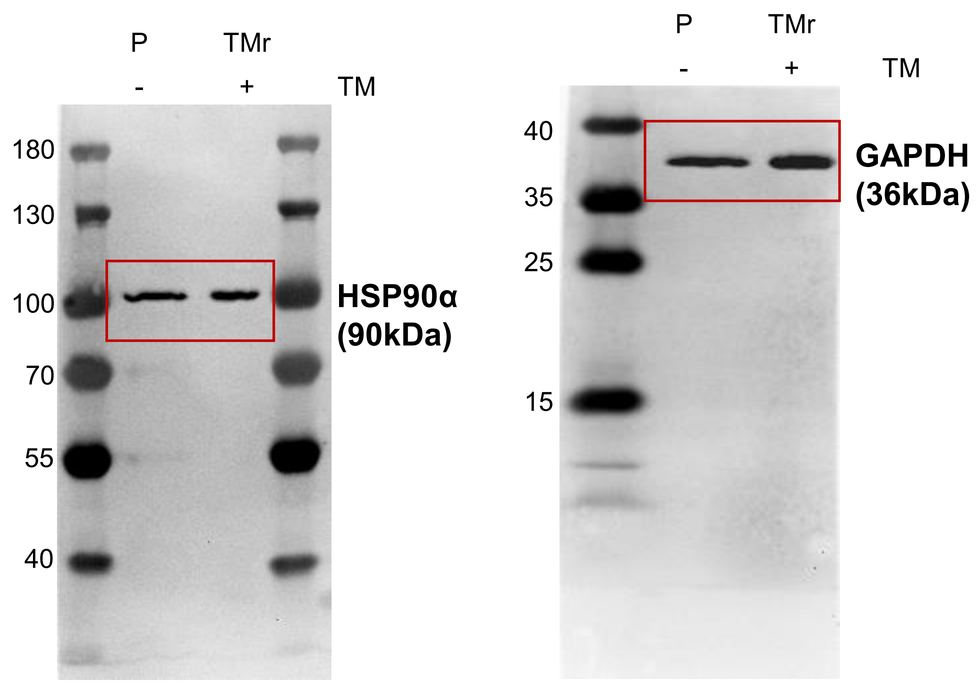

Suppl. Figure 4D.1

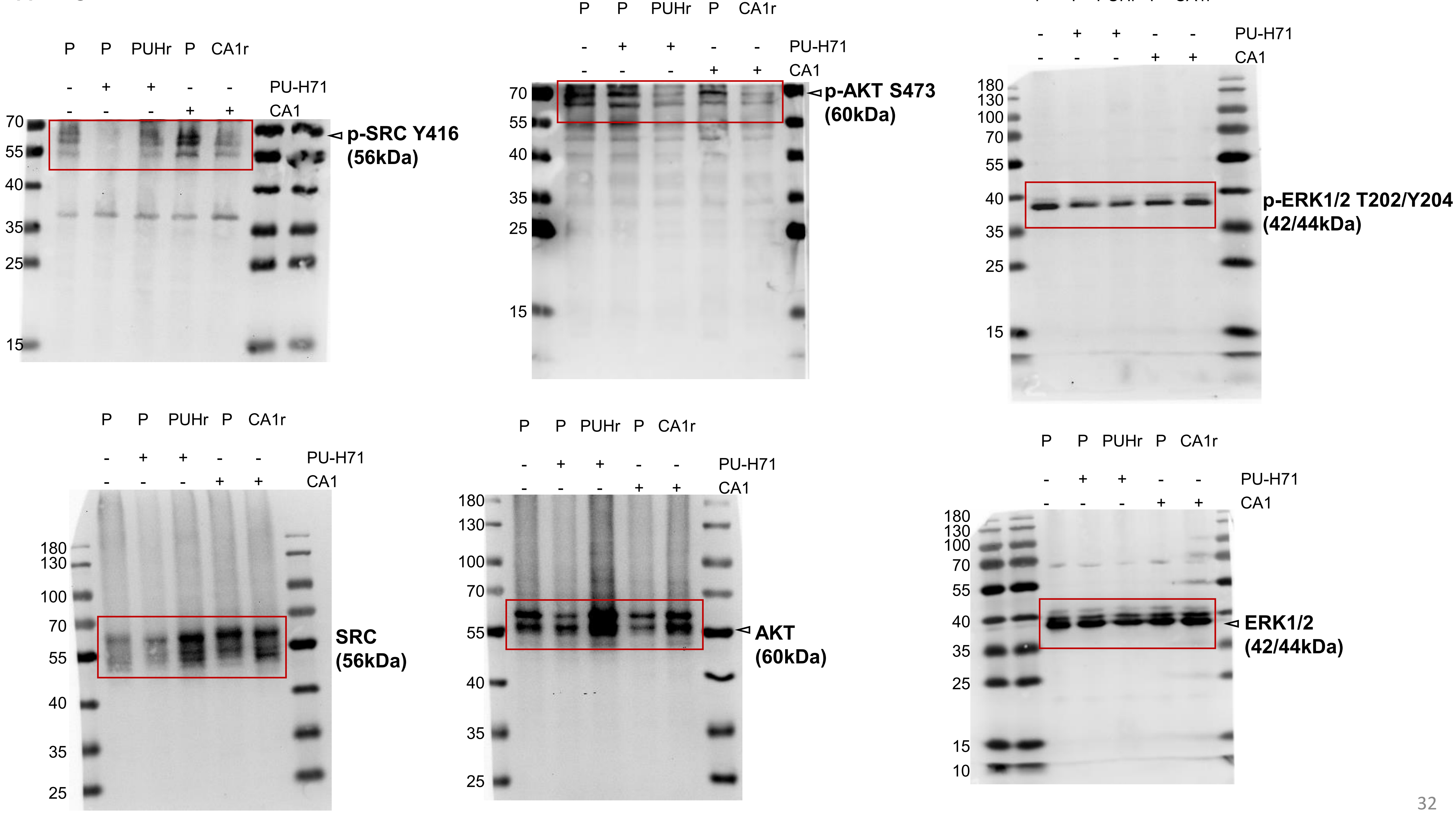

Suppl. Figure 4D.2

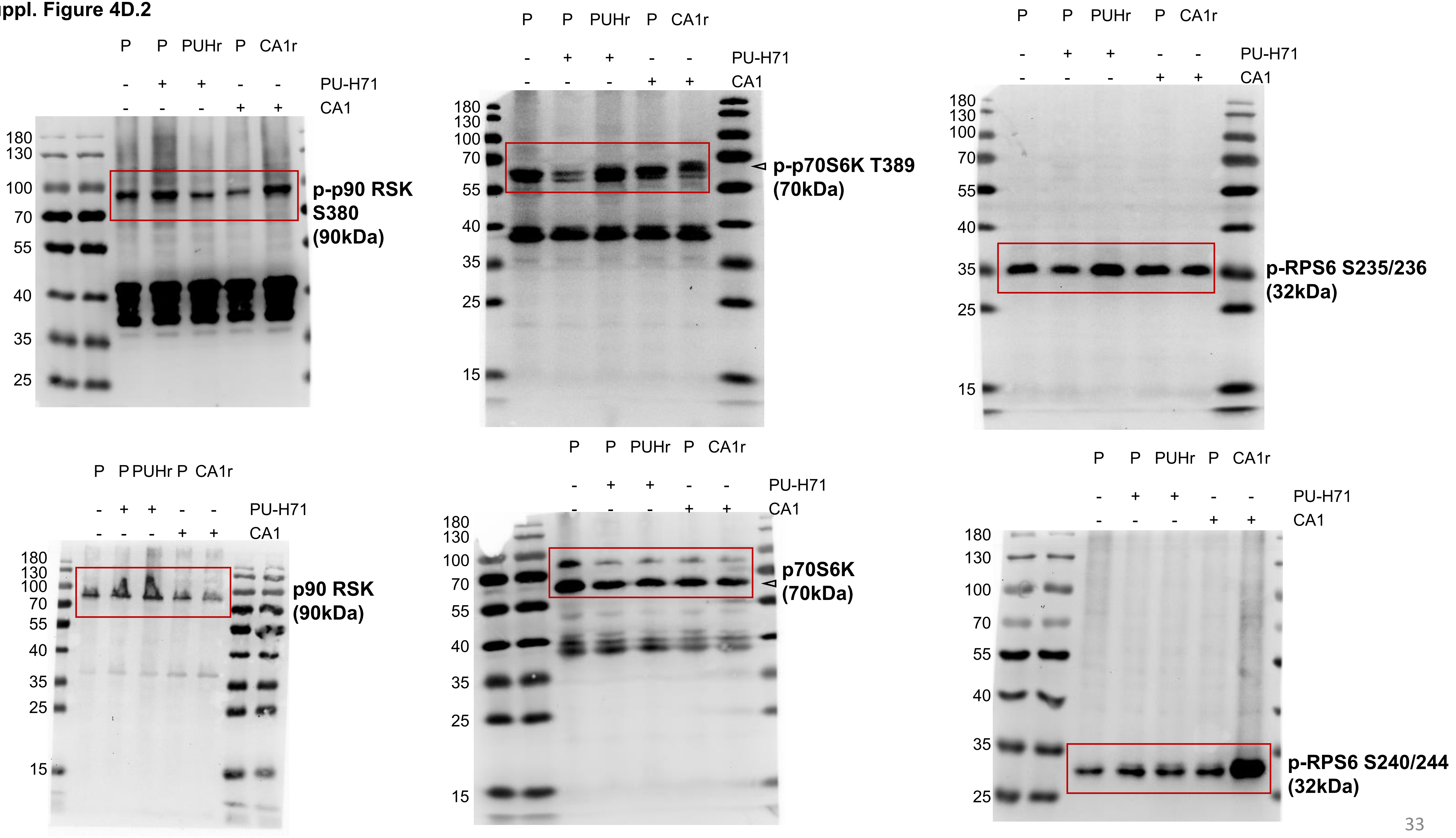

Suppl. Figure 4D.3

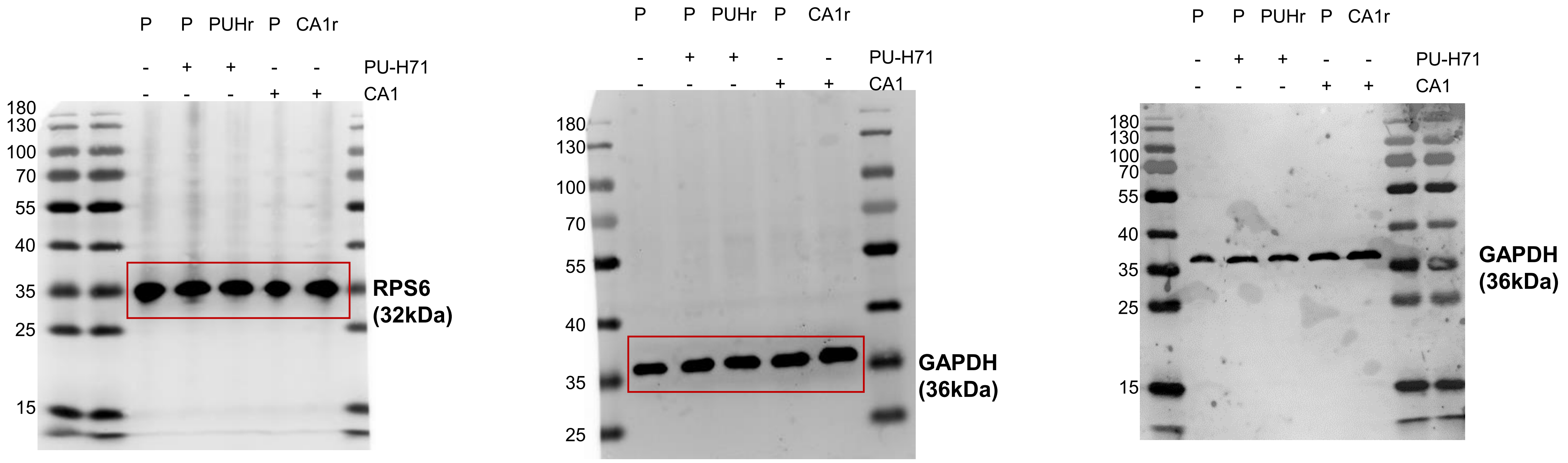

Main Figure 5F - top

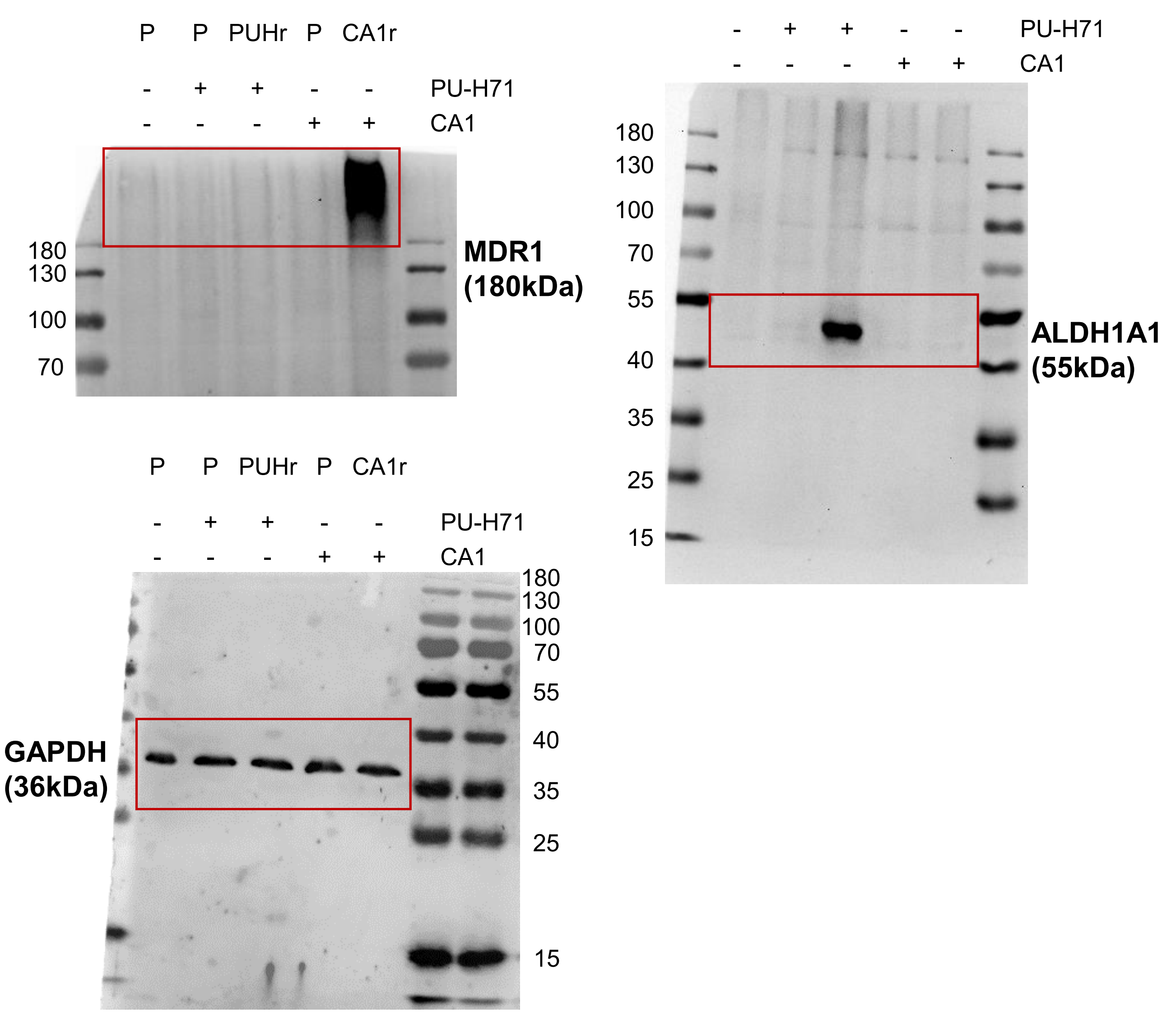

Main Figure 5F - bottom

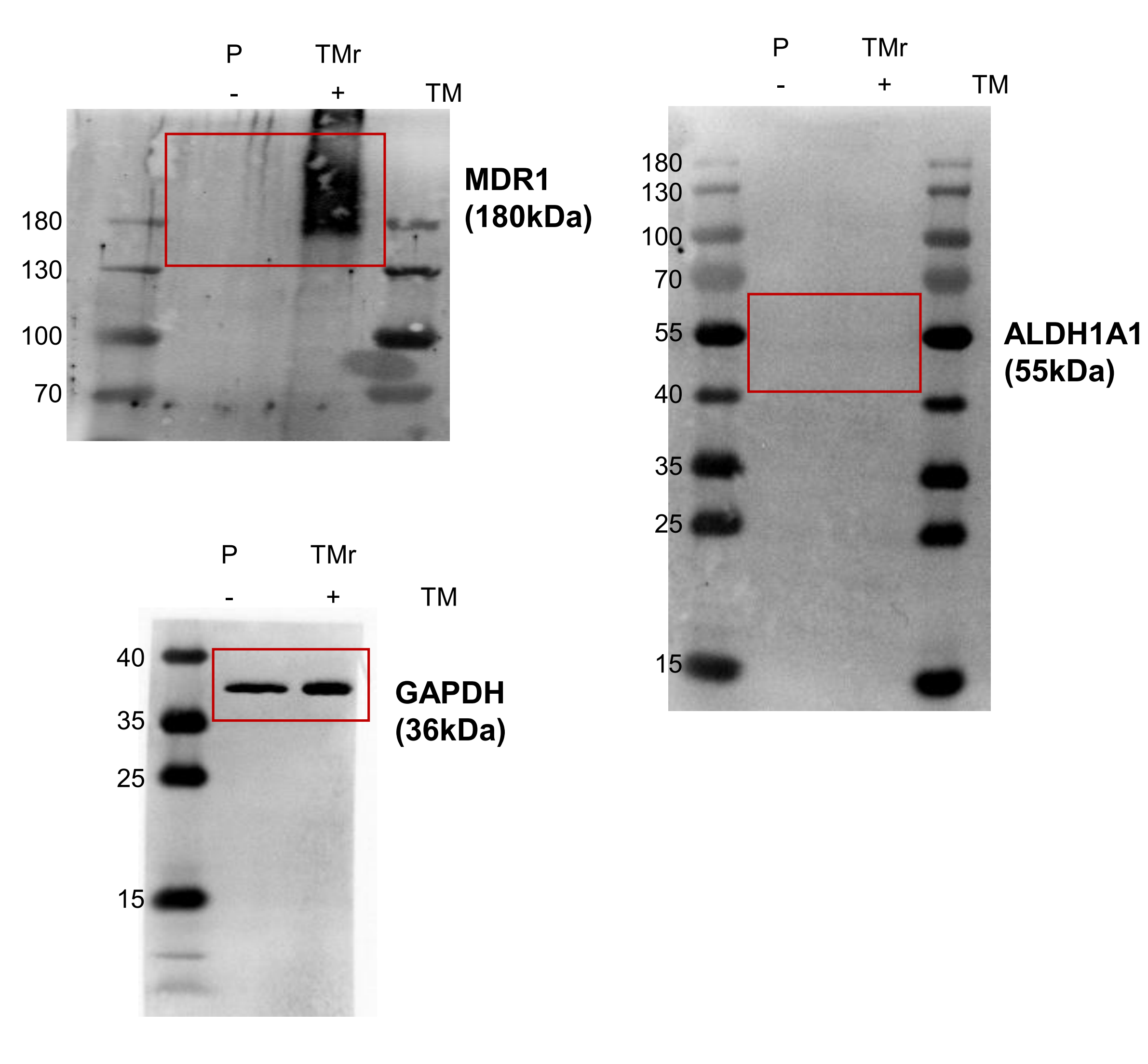

Main Figure 6C

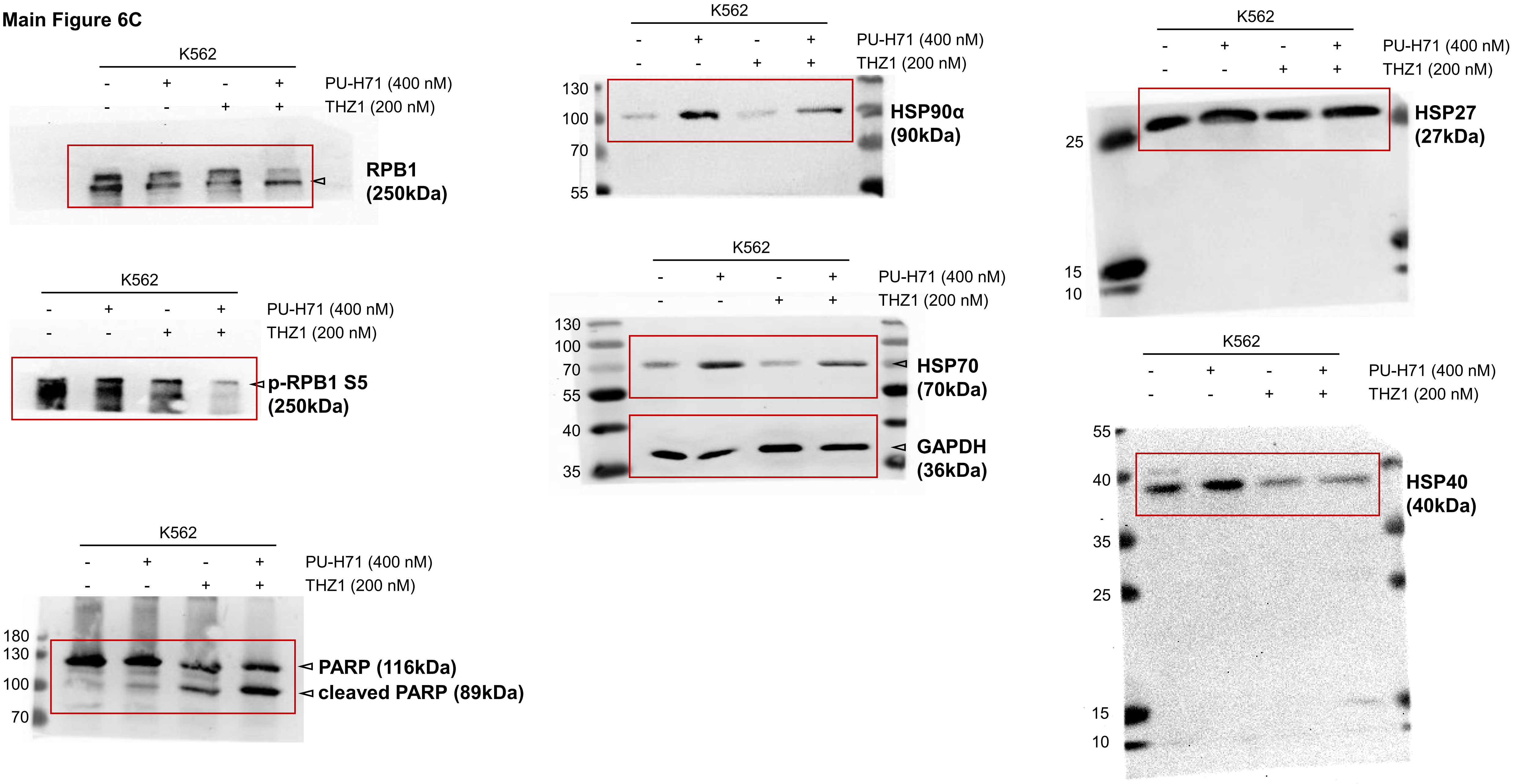

Main Figure 6C.replicates with marker

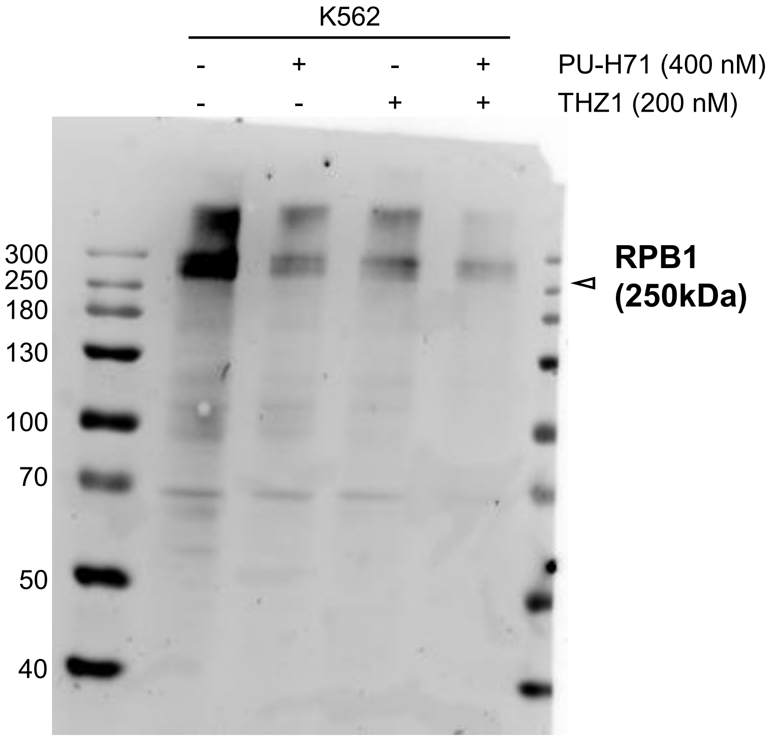

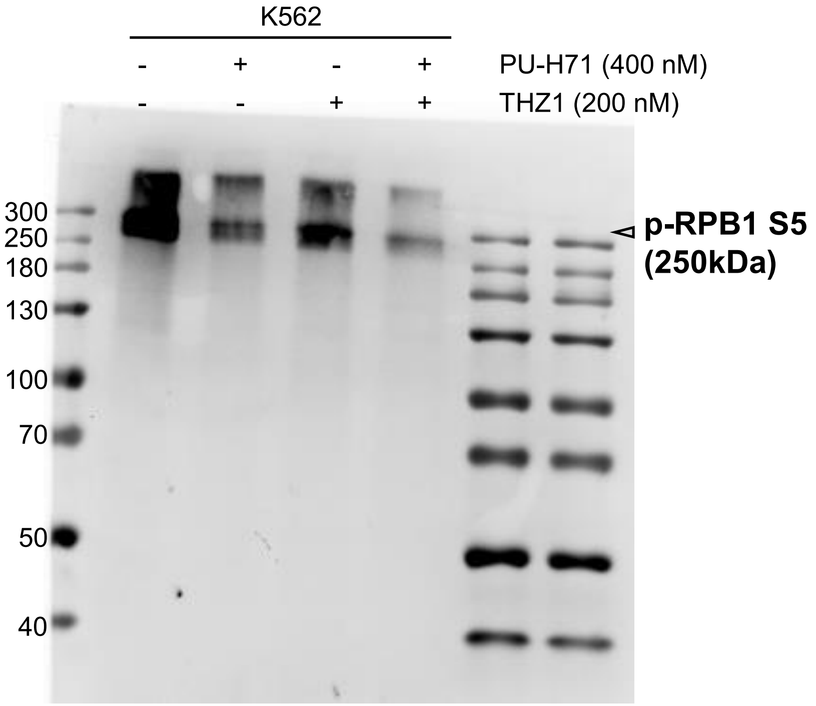

Main Figure 6D

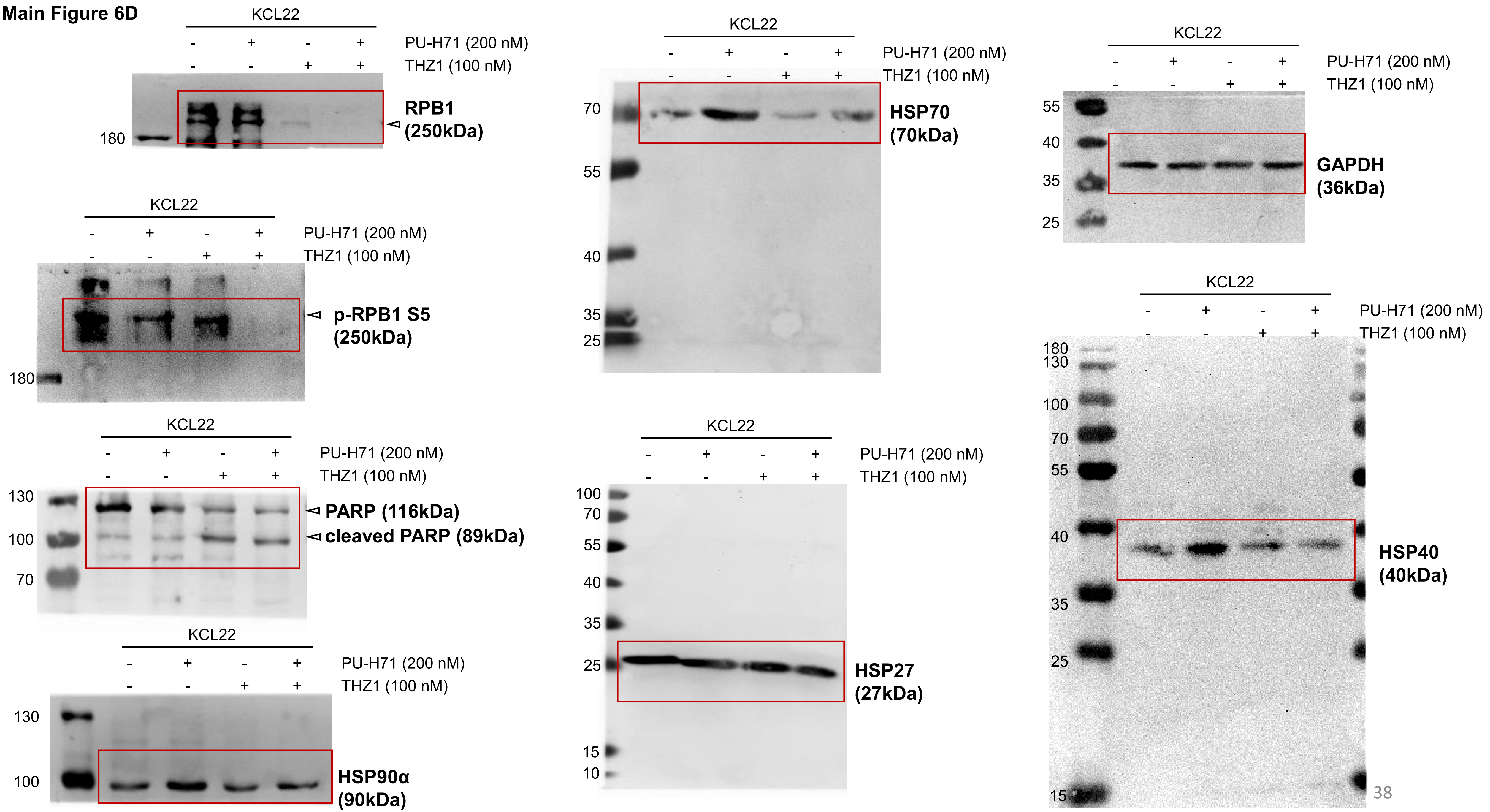

Main Figure 6F

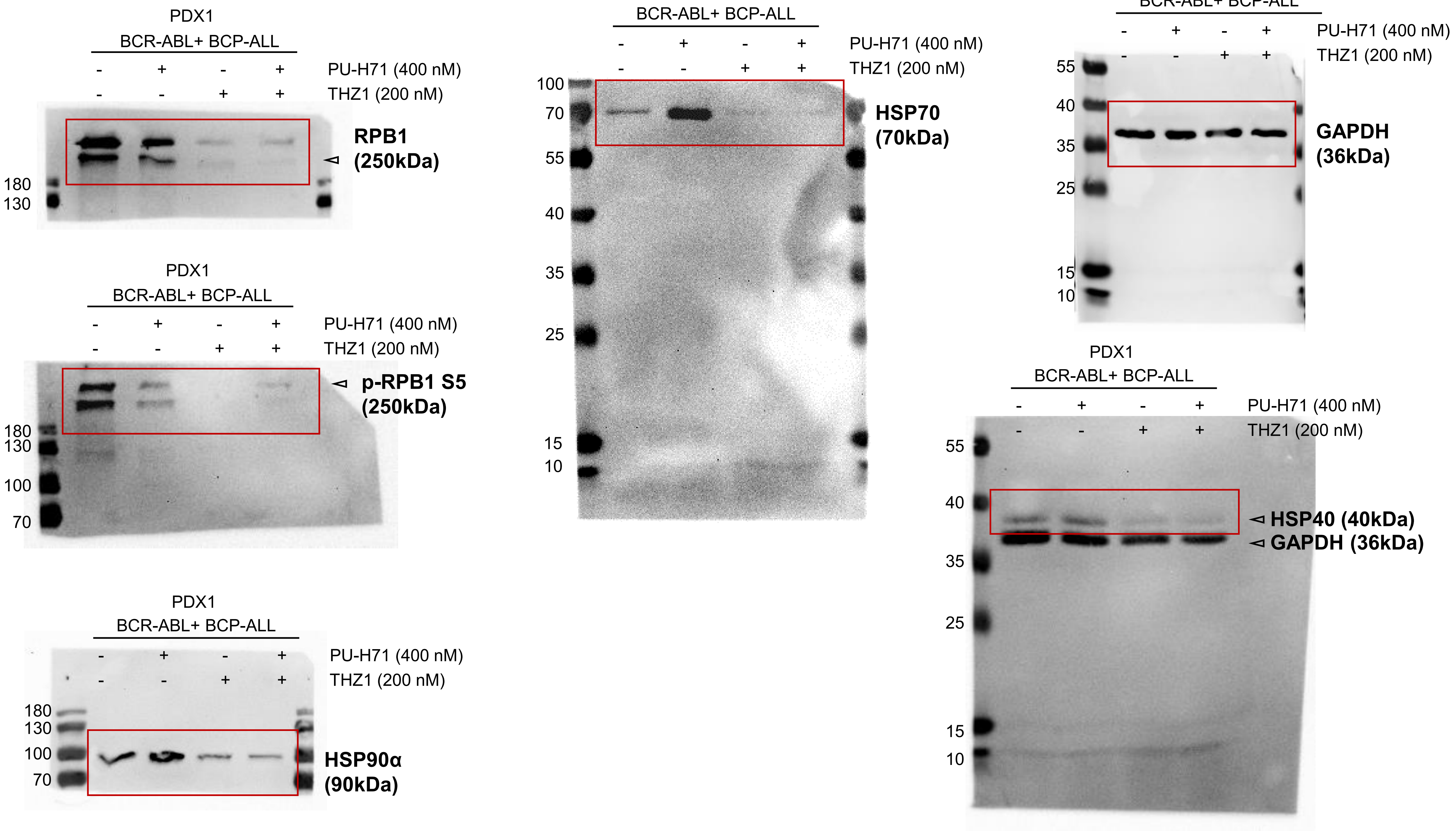

Suppl. Figure 6A

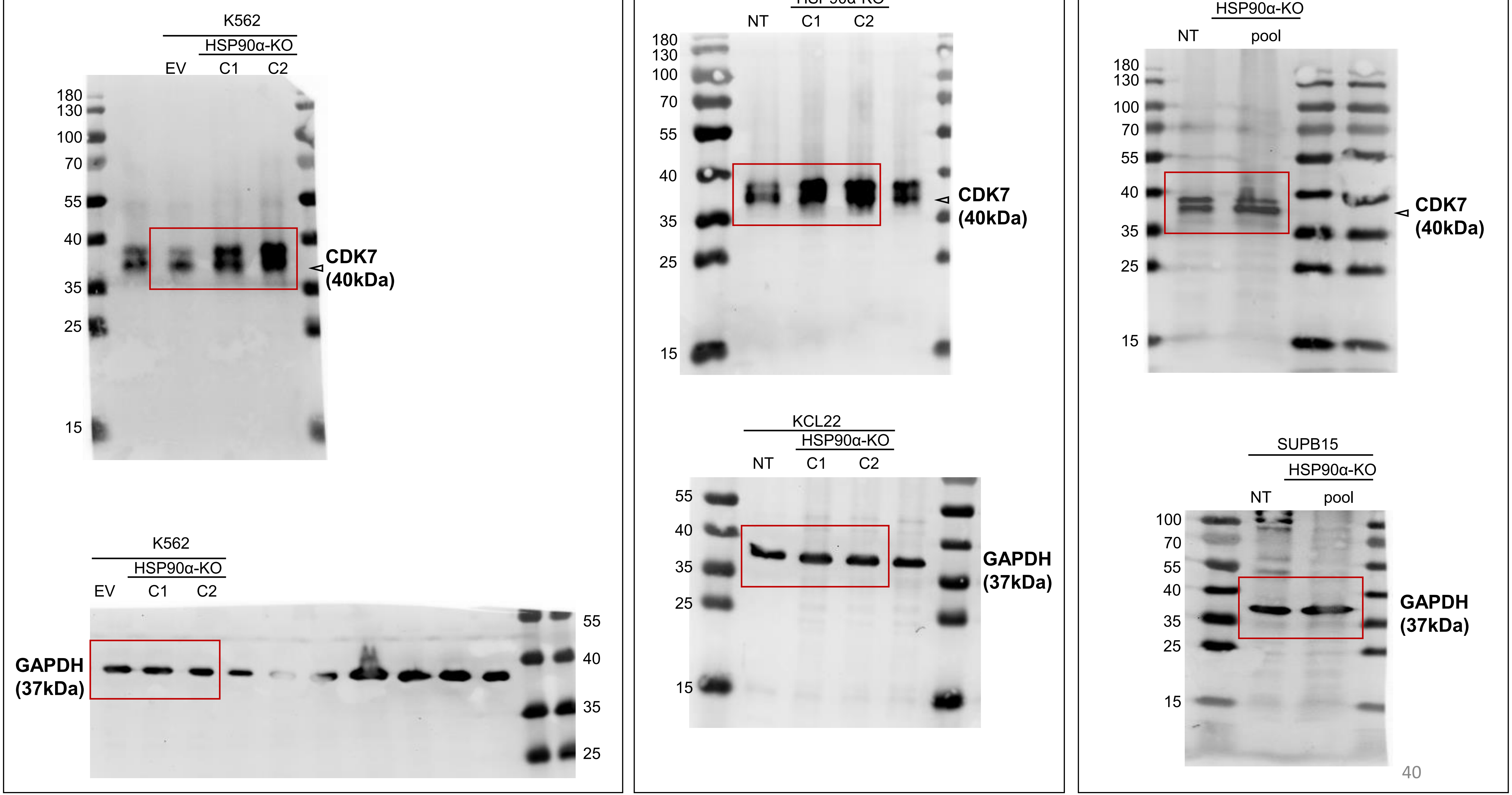

Suppl. Figure 6C

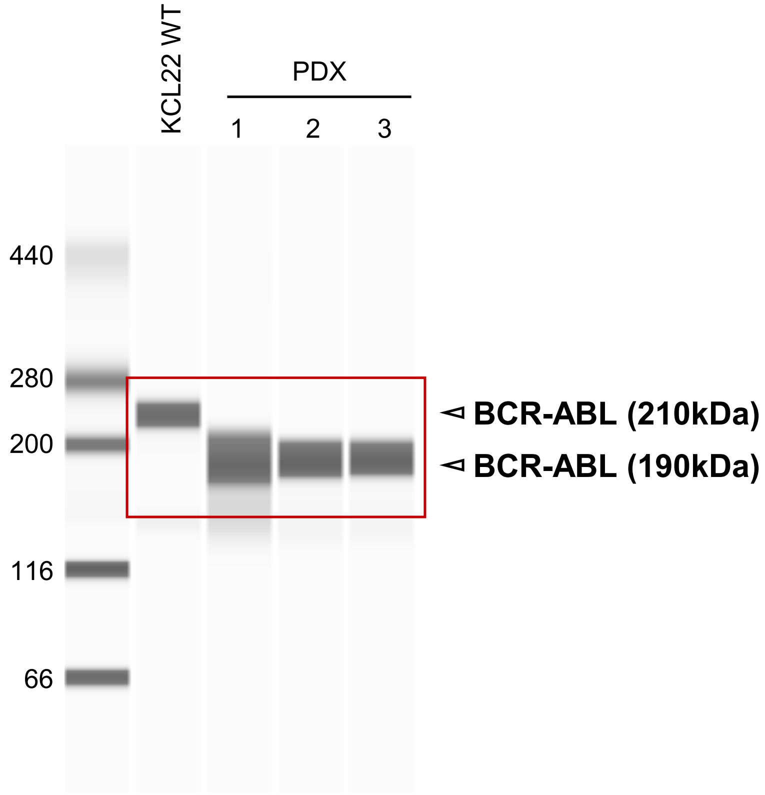

Supplement: Supplementary file 2 — Uncropped western blots [file 41419_2023_6337_MOESM2_ESM.pdf]
